# Sargassopenillines A–G, 6,6-Spiroketals from the Alga-Derived Fungi *Penicillium thomii* and *Penicillium lividum*

**DOI:** 10.3390/md12125930

**Published:** 2014-12-09

**Authors:** Olesya I. Zhuravleva, Maria P. Sobolevskaya, Shamil Sh. Afiyatullov, Natalya N. Kirichuk, Vladimir A. Denisenko, Pavel S. Dmitrenok, Ekaterina A. Yurchenko, Sergey A. Dyshlovoy

**Affiliations:** 1 G.B. Elyakov Pacific Institute of Bioorganic Chemistry, Far-Eastern Branch of the Russian Academy of Sciences, Prospect 100-let Vladivostoku 159, Vladivostok 690022, Russia; E-Mails: zhuravleva.oi@dvfu.ru (O.I.Z.); sobolevskaya_m@mail.ru (M.P.S.); slinkina@piboc.dvo.ru (N.N.K.); vladenis@piboc.dvo.ru (V.A.D.); paveldmt@piboc.dvo.ru (P.S.D.); dminae@mail.ru (E.A.Y.); dyshlovoy@gmail.com (S.A.D.); 2 Far Eastern Federal University, Suhanova 8, Vladivostok 690950, Russia

**Keywords:** marine fungi, *Penicillium thomii*, *Penicillium lividum*, 6,6-spiroketals

## Abstract

Seven new 6,6-spiroketals, sargassopenillines A–G (**1**–**7**) were isolated from the alga-derived fungi *Penicillium thomii* KMM 4645 and *Penicillium lividum* KMM 4663. The structures of these metabolites were determined by HR-MS and 1D and 2D NMR. The absolute configurations of compounds **1**, **5** and **6** were assigned by the modified Mosher’s method and by CD data. Sargassopenilline C (**3**) inhibited the transcriptional activity of the oncogenic nuclear factor AP-1 with an IC_50_ value of 15 µM.

## 1. Introduction

**Figure 1:**
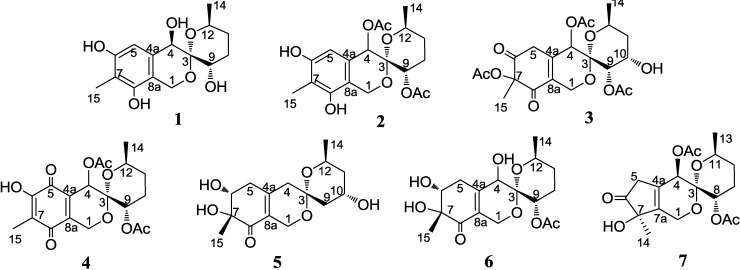
Structures of sargassopenillines A–G (**1**–**7**).

Marine fungi isolated from the surface of marine algae have received great attention as a prolific source of chemically diverse bioactive metabolites [1,2]. As a part of our ongoing search for structurally novel and bioactive metabolites from marine-derived fungi, we have previously isolated ten new austalide meroterpenoids from the strains of *Penicillium thomii* KMM 4645 and *Penicillium lividum* KMM 4663 associated with the marine brown alga *Sargassum miyabei* [3]. Further investigation of metabolites of these fungal strains has now led to the isolation of seven new 6,6-spiroketals, sargassopenillines A–G. We report herein the isolation and structure determination of compounds **1**–**7** (Figure 1) and their biological assay results.

## 2. Results and Discussion

### 2.1. Structure Elucidation

The fungi were cultured for 21 days on specially modified rice medium [4]. The EtOAc extracts of the mycelia were purified by a combination of silica gel column chromatography and reversed-phase HPLC to yield compounds **1** and **5** from the *P. thomii* and **2**–**7** from the *P. lividum* as amorphous solids.

The molecular formula of compound **1** was determined as C_15_H_20_O_6_ by a HRESIMS peak at *m/z* 319.1157 [M + Na]^+^ and by ^13^C NMR analyses. A close inspection of ^1^H and ^13^C NMR data of **1** (Table 1 and Table 2) by DEPT and HSQC revealed the presence of two methyl (δ_H_ 1.08, 2.06, δ_C_ 22.1, 9.2) groups, three methylenes (δ_C_ 37.3, 40.9 and 60.8) including one oxygen-bearing, three oxygenated methines (δ_H_ 3.87, 4.15, 4.19, δ_C_ 71.5, 66.1, 64.1) and one olefinic methine (δ_H_ 6.36, δ_C_ 109.7), five *sp^2^* (δ_C_ 113.0, 114.3, 133.5, 152.3 and 156.3) quaternary carbons including two carbons linked to an oxygen atoms and one double oxygenated quaternary carbon (δ_C_ 100.6).

The HMBC correlations from methyl singlet (δ_H_ 2.06) to oxygenated carbons C-6 (δ_C_ 156.3), C-8 (δ_C_ 152.3) and C-7 (δ_C_ 113.0); from H-5 (δ_H_ 6.36) to C-4 (δ_C_ 71.5), C-4a (δ_C_ 114.3), C-6, C-7, and C-8a (δ_C_ 133.54); from H-4 (δ_H_ 3.87) to C-8a and double oxygenated C-3 (δ_C_ 100.6); and from H_2_-1 (δ_H_ 4.52, 4.74) to C-4a, C-8 and C-3 revealed the connection of C-1 to C-3 through an oxygen atom and indicated the presence of a bicyclic isochroman core in **1** with alcohol functions at C-4, C-6 and C-8 and a methyl group at C-7. The COSY-45 data and HSQC spectra of **1** revealed the connectivity sequences of the protons: (-CH(O)(9)-CH_2_(10)-CH_2_(11)-CH(CH_3_)(12)-). These data and HMBC correlations H-9 (δ_H_ 4.15)/C-3, C-10 (δ_C_ 37.3), C-11 (δ_C_ 40.9); H_3_-14 (δ_H_ 1.08)/C-11, C-12 (δ_C_ 64.1), and H-12 (δ_H_ 4.19)/C-3 indicated the presence of the 6,6-spiroketal moiety in **1**.

**Table 1:** ^1^H NMR spectroscopic data (δ, *J* in Hz) for sargassopenillines A–F (**1**–**6**).

Position	1 ^a^	2 ^b^	3 ^c^	4 ^b^	5 ^b^	6 ^c^
1	a: 4.74, d (14.7)	a: 4.88, d (15.0)	a: 4.70, td (2.8, 16.6)	a: 4.73, d (19.7)	a: 4.53, dd (1.6, 15.5)	a: 4.55, td (2.7, 16.6)
	b: 4.52, d (14.7)	b: 4.57, d (15.0)	b: 4.25, d (16.8)	b: 4.35, dd (1.7, 19.7)	b: 4.11, m	b: 4.05, dd (4.2, 16.6)
4	3.87, s	5.75, s	5.31, s	5.92, d (1.7)	a: 2.40, brd (19.3)	3.72, brs
					b: 2.22, brd (19.3)	
5	6.36, s	6.54, s	a: 3.40, td (2.7, 13.9)		a: 2.53, dd (5.5, 18.3)	a: 3.03, dd (5.6, 18.2)
			b: 3.33, d (8.6)		b: 2.38, brd (19.3)	b: 2.37, m
6					4.00, dd (5.8, 10.5)	4.00, dd (5.7, 10.3)
9	4.15, t (3.0)	5.07, t (2.8)	4.99, d (3.0)	4.95, t (2.8)	a: 1.96, dd (2.1, 14.3)	5.02, t (2.9)
					b: 1.75, dd (3.6, 14.3)	
10	a: 2.24, td (2.2, 14.8)	a: 2.16, m	3.90, brs	a: 2.07, m	4.10, m	a: 2.08, m
	b: 1.72, dd (3.8, 14.8)	b: 1.84, m		b: 1.81, dd (3.2, 14.5)		b: 1.89, dd (3.5, 14.6)
11	a: 1.74, m	a: 1.55, m	a: 1.71, dd (2.9, 11.2)	a: 1.54, m	a: 1.83, dd (2.6, 13.7)	a: 1.55, m
	b: 1.45, m	b: 1.45, m	b: 1.74, td (2.9, 14.2)	b: 1.44, m	b: 1.43, ddd (2.6, 11.9, 13.7)	b: 1.46, m
12	4.19, m	3.98, m	4.11, m	3.78, m	4.12, m	3.78, m
14	1.08, d (6.3)	1.13, d (6.3)	1.23, d (6.3)	1.15, d (6.3)	1.17, d (6.3)	1.14, d (6.3)
15	2.01, s	2.11, s	1.57, s	1.95, s	1.28, s	1.27, s
4-OAc		1.98, s	2.08, s	1.97, s		
7-OAc			2.16, s			
9-OAc		2.08, s	2.05, s	2.06, s		2.16, s

^a^ Chemical shifts referenced to CD_3_OD at 500 MHz; ^b^ Chemical shifts referenced to CDCl_3_ at 700 MHz; ^c^ Chemical shifts referenced to CDCl_3_ at 500 MHz.

**Table 2:** ^13^C NMR spectroscopic data (δ in ppm) for sargassopenillines A–F (**1**–**6**).

Position	1 ^a^	2 ^b^	3 ^c^	4 ^b^	5 ^b^	6 ^c^
1	60.8	58.7	59.0	58.1	57.4	58.3
3	100.6	96.5	98.4	97.1	96.8	97.3
4	71.5	66.1	64.6	59.4	40.6	65.9
4a	114.3	128.7	130.2	144.2	149.5	149.2
5	109.7	108.9	40.4	180.6	35.9	33.9
6	156.3	153.3	198.0	151.2	72.3	72.5
7	113.0	110.1	84.7	117.4	77.3	77.4
8	152.3	149.7	192.2	186.0	198.9	200.2
8a	133.5	113.1	139.8	130.7	126.6	127.9
9	66.1	66.2	65.5	65.0	39.1	66.5
10	37.3	24.2	65.8	24.0	64.7	24.0
11	40.9	26.8	34.7	26.5	39.2	26.7
12	64.1	68.4	63.5	69.1	61.6	68.9
14	22.1	21.3	20.8	21.2	21.2	21.3
15	9.2	7.8	21.3	7.7	17.7	17.4
4-OAc		171.1, 21.2	170.5, 20.7	168.3, 20.7		
7-OAc			169.5, 19.9			
9-OAc		170.3, 21.1	169.3, 20.9	170.5, 21.4		170.7, 21.3

^a^ Chemical shifts referenced to CD_3_OD at 125 MHz; ^b^ Chemical shifts referenced to CDCl_3_ at 176 MHz; ^c^ Chemical shifts referenced to CDCl_3_ at 125 MHz.

**Figure 2:**
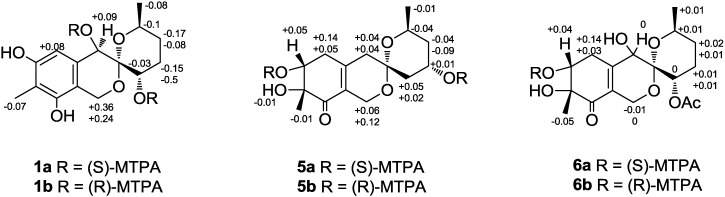
The ∆δ (δ_S_−δ_R_) values (in ppm) for the (*S*)- and (*R*)-MTPA esters of **1**, **5** and **6**.

**Figure 3:**
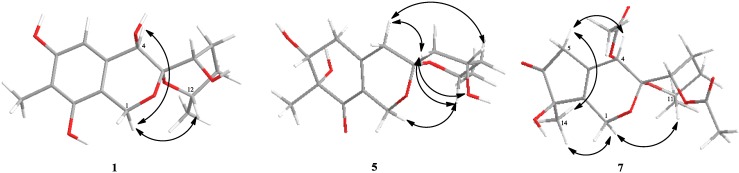
Chem3D representation of the minimum conformation of **1**, **5** and **7** showed observed NOE correlations.

Esterification of **1** with (*R*)- and (*S*)-MTPA chloride [5] occurred both at the C-4 and C-9 hydroxy groups to give the (*S*)-and (*R*) MTPA esters **1a** and **1b**, respectively. The observed chemical shift differences Δδ(δ*_S_*-δ*_R_*) (Figure 2) indicated the 4*R* and 9*S* configuration. The revealed configuration of the C-9 chiral center in **1** and analysis coupling constants for H-9 (δ_H_ 4.15, t, 3.0) and H_2_-10 (Ha: δ_H_ 2.24, td, 2.2, 14.8; Hb: δ_H_ 1.72, dd, 3.8, 14.8), that were also calculated using the empirical generalization of the classical Karplus equation [6], showed that the right ring is in the pseudo boat conformation (Figure 3). These data and NOE correlations H-12/H-1b (δ_H_ 4.52) (2.957 Å) [7] and H-4/H-1b (4.435 Å) determined the absolute stereostructure of **1** with 3*S*, 4*R*, 9*S*, 12*S* configurations. Compound **1** was named sargassopenilline A.

The molecular formula of **2** was determined to be C_19_H_24_O_8_ by a HRESIMS peak at *m/z* 403.1366 [M + Na]^+^ and was in accordance with ^13^C NMR data (Table 2). The structure of the substituted bicyclic isochroman moiety of **2** was found by extensive NMR spectroscopy (^1^H and ^13^C NMR, COSY, HSQC and HMBC) as for sargassopenilline A (**1**). The HMBC correlations from H-4 (δ_H_ 5.75) to 4-Ac (δ_C_ 171.1), from 4-Ac (CH_3_, δ_H_ 1.98) to C-4 (δ_C_ 66.1), 4-Ac (δ_C_ 171.1) and downfield chemical shift of H-4 indicated replacement the alcohol group at C-4 by the acetoxy group. The COSY-45 data and HMBC correlations H-9 (δ_H_ 5.07)/C-3 (δ_C_ 96.5), 9-Ac (δ_C_ 170.3), C-10 (δ_C_ 24.2), C-11 (δ_C_ 26.8); H_3_-14 (δ_H_ 1.13)/C-11, C-12 (δ_C_ 68.4) and H-12 (δ_H_ 3.98)/C-3 established the structure of the 9-acetoxy-12-methylpyran ring and indicated the presence of spirocyclic system in **2**. The NOE correlation H-12/ H-1b (δ_H_ 4.57) indicated a β-orientation for the 14-methyl group and suggested the configuration of C-3 to be *S*. The small coupling constants of the H-9 signal at δ 5.07 (1H, t, 2.8) and biogenetic relationship between sargassopenilline A and **2** suggested an α-orientation for the 9-acetoxy group. Unfortunately, the correlations observed in the NOESY spectrum did not allow us to unequivocally establish the relative configuration at C-4. Compound **2** was named sargassopenilline B.

The molecular formula of **3** was determined to be C_21_H_26_O_11_ by a HRESIMS peak at *m/z* 477.1365 [M + Na]^+^ and was in accordance with ^13^C NMR data. The ^1^H and ^13^C NMR spectra of **3** (Table 1 and Table 2) by DEPT and HSQC indicated the presence of two methyl (δ_H_ 1.23, 1.57, δ_C_ 20.83, 21.3) and three acetoxy (δ_H_ 2.05, δ_C_ 20.9, 169.3, δ_H_ 2.08, δ_C_ 20.7, 170.5, δ_H_ 2.16, δ_C_ 19.9, 169.5) groups, three methylenes (δ_C_ 34.7, 40.4, and 59.0) including one oxygen-bearing, four oxygenated methines (δ_H_ 3.90, 4.11, 4.99, 5.31 δ_C_ 65.8, 63.5, 65.5, 64.6), two carbonyl functions (δ_C_ 192.2 and 198.0), one tetrasubstituted double bond (δ_C_ 130.2 and 139.8) and one double oxygenated quaternary carbon (δ_C_ 98.4). The HMBC correlations H_3_-15 (δ_H_ 1.57)/C-6 (δ_C_ 198.0), C-7 (δ_C_ 84.7), C-8 (δ_C_ 192.2); H-5a (δ_H_ 3.40)/C-4 (δ_C_ 64.6), C-4a (δ_C_ 130.2), C-6, C-7 and H-1a (δ_H_ 4.70)/C-3 (δ_C_ 98.4), C-4, C-4a, C-8 and C-8a (δ_C_ 139.8) indicated the presence of the bicyclic isochromene core in **3** with carbonyl functions at C-6 and C-8 and a methyl group at C-7. The locations of the acetoxy groups at C-4 and C-7 were evident from the HMBC correlations from 4-Ac (δ_H_ 2.08) to C-4 (δ_C_ 64.6), 4-Ac (δ_C_ 170.5) and from 7-Ac (δ_H_ 2.16) to C-7 (δ_C_ 84.7), 7-Ac (δ_C_ 169.5). The interpretation of the COSY and HSQC data revealed one isolated spin system: (-CH(O)(9)-CH(O)(10)-CH_2_(11)-CH(CH_3_)(12)-). These data and the HMBC correlations H-9 (δ_H_ 4.99)/C-3 (δ_C_ 98.4), 9-Ac (δ_C_ 169.3), C-10 (δ_C_ 65.8), C-11 (δ_C_ 34.7); H-11a (δ_H_ 1.74)/C-10; H-11b (δ_H_ 1.71)/C-12 (δ_C_ 63.5); H_3_-14 (δ_H_ 1.23)/C-3, C-11 and C-12 established the presence of the 9-acetoxy-10-hydroxy-12-methylpyran ring in **3**.

The small coupling of the H-10 signal at δ 3.90 (1H, brs) and downfield chemical shift of C-12 (δ_C_ 63.5) in comparison with the spectra of **2** (δ_C_ 68.4) (γ-effect) indicated that **3** contained a secondary alcohol function with an α-orientation. Furthermore, NOE correlations H-1b (δ_H_ 4.25)/H-12 indicated a β-orientation for the 14-methyl group and suggested the configuration of C-3 to be *S*. We observed a strong NOE correlation between H-9 and H-10 and small coupling of the H-9 signal at δ 4.99 (1H, d, 3.0) proposed to be an α-orientation for the 9-acetoxy group. The configurations at C-4 and C-7 have not been determined. Compound **3** was named sargassopenilline C.

The molecular formula of **4** was determined to be C_19_H_22_O_9_ by a HRESIMS peak at *m/z* 417.1157 [M + Na]^+^ and was in accordance with ^13^C NMR data. The ^1^H and ^13^C NMR (Table 1 and Table 2), DEPT and HSQC spectra showed two methyls, three methylenes (one of them oxygenated), three oxymethines, two ketone carbons, four olefinic quaternary carbons, one doubled oxygenated quaternary carbon, one hydroxyl and two acetoxy groups. The HMBC correlations H_3_-15 (δ_H_ 1.95)/C-6 (δ_C_ 151.2), C-7 (δ_C_ 117.4), C-8 (δ_C_ 186.0); 6-OH (δ_H_ 6.95)/C-5 (δ_C_ 180.6), C-6, C-7, H-1a (δ_H_ 4.73)/C-3 (δ_C_ 97.1), C-4a (δ_C_ 144.2), C-8, C-8a (δ_C_ 130.7) and H-4 (δ_H_ 5.92)/C-3, C-4a, C-5 and C-8a indicated the presence of the bicyclic isochromene core in **4** with carbonyl functions at C-5 and C-8 and hydroxyl and methyl groups at C-6 and C-7, respectively. The location of the acetoxy group at C-4 was evident from the HMBC correlations from 4-Ac (δ_H_ 1.97) to C-4 (δ_C_ 59.4) and 4-Ac (δ_C_ 168.3). The structure of the 9-acetoxy-12-methyl pyran ring and the presence 6,6-spiroring system in **4** was found by extensive NMR spectroscopy (^1^H and ^13^C NMR, COSY, HSQC and HMBC) as for sargassopenilline B (**2**). The NOE correlation H-12/ H-1b (δ_H_ 4.35) indicated a β-orientation for the 14-methyl group and suggested the configuration of C-3 to be *S*. The small coupling constants of the H-9 signal at δ 4.95 (1H, t, 2.8) and biogenetic relationship between sargassopenilline A and **4** suggested an α-orientation for the 9-acetoxy group. The configuration at C-4 has not been determined. Compound **4** was named sargassopenilline D.

The molecular formula of **5** was determined to be C_15_H_22_O_6_ by a HRESIMS peak at *m/z* 299.1483 [M + H]^+^ and was in accordance with ^13^C NMR data. The ^1^H and ^13^C NMR spectra (Table 1 and Table 2) of **5** indicated the presence of two methyls, five methylenes including one oxygen-bearing, three oxygenated methines and one double oxygenated quaternary carbon. The remaining functionalities, corresponding to the carbon signals at δ_C_ 198.9 (C), 149.5 (C) and 126.6 (C), suggested the presence of a carbonyl carbon and one tetrasubstituted double bond. The ^1^H and ^13^C data observed for the 3,4,5,6,7,8-hexahydroisochromene core resemble those reported for pestafolide A [8]. The interpretation of the COSY and HSQC data revealed one isolated spin system: (-CH_2_(9)-CH(O)(10)-CH_2_(11)-CH(CH_3_)(12)-). This information and the HMBC correlations H-9a (δ_H_ 1.96)/C-3 (δ_C_ 96.8), C-11 (δ_C_ 39.2); H-9b (δ_H_ 1.75)/C-3, C-4 (δ_C_ 40.6), C-10 (δ_C_ 64.7); H-10 (δ_H_ 4.10)/C-3; H_3_-14 (δ_H_ 1.08)/C-11, C-12 (δ_C_ 61.6) indicated the presence of the 10-hydroxy-12-methylpyran ring in **5**. Thus, the planar structure of **5** was established.

Compound **5** showed the characteristic Cotton effects (CEs) at λ_310_ +0.29, λ_248_ −5.70 and λ_216_ +7.57 in the CD spectra in methanol solution. The two CEs of the high-energy region were in agreement with those for pestafolide A [8] and peneciraistin C [7], supporting the 7*R* configuration of **5**. Esterification of **5** with (*R*)- and (*S*)-MTPA chloride occurred both at the C-6 and C-10 hydroxy groups to give the (*S*)- and (*R*)-MTPA esters **5a** and **5b**, respectively. The observed chemical shift differences Δδ(δ*_S _* − δ*_R_*) (Figure 2) revealed the 6*R* and 10*S* configurations. These data and the NOE correlations, recorded in DMSO-d_6_ solvent, (Supplementary Figure S44, Figure 3) H-1b (δ_H_ 3.93)/7-OH (δ_H_ 5.04), H-12 (δ_H_ 4.07); H-1a (δ_H_ 4.20)/H-4a (δ_H_ 2.35); H-4b (δ_H_ 2.08)/H-9b (δ_H_ 1.63), H_3_-14 (δ_H_ 1.05) and H-9a (δ_H_ 1.77)/10-OH (δ_H_ 4.15), H-12 determined the absolute stereostructure of **5** with 3*S*, 6*R*, 10*S*, and 12*S* configurations. Compound **5** was named sargassopenilline E.

The molecular formula of **6** was determined to be C_17_H_24_O_8_ by a HRESIMS peak at *m/z* 357.1548 [M + H]^+^ and was in accordance with ^13^C NMR data. The general features of the ^1^H and ^13^C NMR spectra (Table 1 and Table 2) of the isochromene core in **6** resembled those of **5** with the exception of the C-4 and C-5 proton and carbon signals. The HMBC correlations H-1a (δ_H_ 4.55)/C-3 (δ_C_ 97.3), C-4 (δ_C_ 65.9), C-4a (δ_C_ 149.2), C-8 (δ_C_ 200.2) and C-8a (δ_C_ 127.9); H-4 (δ_H_ 3.72)/C-4a, C-5 (δ_C_ 33.9), C-8a; H-5a (δ_H_ 3.03)/C-6 (δ_C_ 72.5) and C-8a; H-6 (δ_H_ 4.00)/C-5, C-7 (δ_C_ 77.4), C-8 and C-15 (δ_C_ 17.4) indicated the location of the hydroxy group at C-4 and established the structure of a bicyclic isochromene core in **6**. The structure of the 9-acetoxy-12-methylpyran ring and the presence 6,6-spiroring system in **6** was found by extensive NMR spectroscopy (^1^H and ^13^C NMR, COSY, HSQC and HMBC) as for sargassopenilline B (**2**).

Compound **6** exhibited a nearly identical CD spectrum in the high-energy region to that of sargassopenilline E (**5**), which allowed us to determine the 7*R* configuration of **6**. Esterification of **6** with (*R*)- and (*S*)-MTPA chloride occurred at the C-6 hydroxy group to give the (*S*)-and (*R*) MTPA esters **6a** and **6b**, respectively. The observed chemical shift differences Δδ(δ*_S_* − δ*_R_*) (Figure 2) revealed the 6*R* configuration. The NOE correlation H-12/H-1b (δ_H_ 4.05) indicated a β-orientation for the 14-methyl group and suggested the configuration of C-3 to be *S*. The small coupling constants of the H-9 signal at δ 5.02 (1H, t, 2.9) and biogenetic relationship between sargassopenilline A and **6** suggested an α-orientation for the 9-acetoxy group. Unfortunately, the correlations observed in the NOESY spectrum could not unequivocally establish the relative configuration at C-4. Compound **6** was named sargassopenilline F.

The molecular formula of compound **7** was determined as C_18_H_24_O_8_ by a HRESIMS peak at *m/z* 391.1367 [M + Na]^+^ and by ^13^C NMR analyses. The ^1^H and ^13^C NMR spectra of **7** (Table 3) by DEPT and HSQC indicated the presence of two methyl (δ_H_ 1.19, 1.36, δ_C_ 21.3, 22.5) and two acetoxy (δ_H_ 2.01, 2.03, δ_C_ 20.7, 21.0, 170.4, 170.0) groups, four methylenes (δ_C_ 24.1, 26.7, 40.1 and 57.5) including one oxygen-bearing, three oxygenated methines (δ_H_ 3.85, 5.02, 5.25, δ_C_ 68.7, 66.2, 64.3) and one double oxygenated quaternary carbon (δ_C_ 97.0). The remaining functionalities, corresponding to the carbon signals at δ_C_ 214.4 (C), 141.8 (C) and 127.3 (C), suggested the presence of a ketone function and one tetrasubstituted double bond.

The HMBC correlations H-5b (δ_H_ 2.92)/C-4a (δ_C_ 127.3), C-6 (δ_C_ 214.4), C-7 (δ_C_ 77.9) and C-7a (δ_C_ 141.8); H_3_-14 (δ_H_ 1.36)/C-6, C-7 and C-7a revealed the presence of 2-hydroxy-2-methylcyclopent-3-enone moiety in **7**. Furthermore, HMBC correlations H-1a (δ_H_ 4.43)/C-3 (δ_C_ 97.0), C-4 (δ_C_ 64.3), C-4a, C-7a and H-4 (δ_H_ 5.25)/C-3, C-4a, C-5 (δ_C_ 40.1), C-7a and 4-Ac (δ_C_ 170.4) show that **7** contains an unusual tetrahydrocyclopentapyranone ring system in the molecular structure. The structure of the 9-acetoxy-12-methylpyran ring and the presence 6,6-spiroring system in **7** was found by extensive NMR spectroscopy (^1^H and ^13^C NMR, COSY, HSQC and HMBC) as for sargassopenilline B (**2**). Thus compound **7** is a new spiroketal-containing natural compound and it was named sargassopenilline G.

**Table 3:** ^1^H (CDCl_3_, 700 MHz) and ^13^C (CDCl_3_, 176 MHz) NMR spectroscopic data for sargassopenilline G (**7**).

Position	δ_C_	δ_H_ (*J* in Hz)	HMBC
1	57.5	a: 4.43, td (3.0, 16.4),	3, 4, 4a, 7, 4, 4a, 7a
		b: 4.23, d (16.4)	
3	97.0		
4	64.3	5.25, s	4a, 5, 7a, 4-Ac (170.4)
4a	127.3		
5	40.1	a: 3.02, td (3.3, 22.2),	4a, 6, 7a, 4a, 6, 7, 7a
		b: 2.92, td (3.1, 22.2)	
6	214.4		
7	77.9		
7a	141.8		
8	66.2	5.02 t (2.8)	3, 8-Ac (170.0), 9, 10
9	24.1	a: 2.10, m, b: 1.84, m	10, 11, 3, 7a, 10, 11
10	26.7	a: 1.52, m, b: 1.46, m	8, 9, 11, 13, 8, 9, 11
11	68.7	3.85, m	3, 9, 13
13	21.3	1.19, d (6.3)	3, 10, 11
14	22.5	1.36, s	6, 7, 7a
4-Ac	170.4, 20.7	2.01, s	4, 4-Ac (170.4)
8-Ac	170.0, 21.0	2.03, s	8, 8-Ac (170.0)

The NOE correlation H-11/H-1b (δ_H_ 4.23) (2.854 Å) indicated a β-orientation for the 13-methyl group and suggested the configuration of C-3 to be *S*. The small coupling constants of the H-8 signal at δ 5.02 (1H, t, 2.8) and biogenetic relationship between sargassopenilline A and **7** suggested an α-orientation for the 8-acetoxy group. These data and the observed NOE correlations H_3_-14/H-1b (δ_H_ 4.23), H-5a (δ_H_ 3.02) and H-5a/H-4 indicated the relative configuration of **7** (Figure 3).

### 2.2. Bioassay Results

Sargassopenillines **1**–**3** and **7** were assayed for their cytotoxic activity against MDA-MB-231 and JB6 Cl41 cell lines. None of the compounds exhibited cytotoxicity (IC_50_ < 100 μM).

The effect of compounds **1**–**3** and **7** on the basal AP-1-dependent transcriptional activity was also studied using JB6 Cl41 cells stably expressing a luciferase reporter gene controlled by an AP-1-DNA binding sequence [9,10,11,12]. We found that compound **3** is able to inhibit the transcriptional activity of the oncogenic nuclear factor AP-1 with IC_50_ value of 15 µM after 12 h of treatment.

The sargassopenillines **1**, **2**, **4**–**7** were assayed for their cytotoxic activity against CD-1 mouse splenocytes and membranolytic activity to erythrocytes up to 100 µM. Sargassopenilline E (**5**) exhibited cytotoxicity against splenocytes with a IC_50_ value 38 µM. The effects of the compounds **1**, **2** and **4**–**7** on the functional activity of CD-1 murine peritoneal macrophages were also studied. It was shown that sargassopenillines D and F at a non-toxic concentration (10 µM) inhibit the adhesion of macrophages (30%–40% of inhibition).

In addition, compounds **1** and **5** showed radical scavenging activity against DPPH with IC_50_ values of 100 and 50 µM, respectively, while others were inactive.

## 3. Experimental Section

### 3.1. General Experimental

Optical rotations were measured on a Perkin-Elmer 343 polarimeter. UV spectra were recorded on a Shimadzu UV-1601PC spectrometer in MeOH. CD spectra were measured with a Chirascan-Plus CD Spectrometer (Leatherhead, UK). IR spectra were determined on a Bruker OPUS Vector-22 infrared spectrophotometer in CHCl_3_. ^1^H and ^13^C NMR spectra were recorded in CDCl_3_, MeOH-d_4_ and pyridine-d_5_ on a Bruker Avance-500 and Avance III-700 spectrometers operating at 500.13 MHz and 125.77 MHz and 700.13 and 176.04 MHz, respectively, using TMS as an internal standard. HRESIMS spectra were measured on an Agilent 6510 Q-TOF LC mass spectrometer.

Low-pressure liquid column chromatography was performed using Si gel L (40/100 μm, Sorbpolimer, Russia). Glass plates (4.5 × 6.0 cm) precoated with Si gel (5–17 μm, Sorbfil) were used for thin layer chromatography. Preparative HPLC was carried out on a Beckman-Altex chromatograph, using a Supelco Discovery C-18 (5 μm, 4.6 × 250 mm) column with an RIDK–122 refractometer.

The energy-minimized conformations for **1**, **5** and **7** have been determined using crystallographic data (CCDC 940798) for the structure of peniciketal A [13] by the MM2 force field calculation method using ChemBioDraw Ultra 12.0, CambridgeSoft Corporation (Cambridge, MA, USA).

### 3.2. Fungal Material and Fermentation

The strains of the fungi *Penicillium lividum* and *Penicillium thomii* were isolated from superficial mycobiota of the brown alga *Sargassum miyabei* (Lazurnaya Bay, the Sea of Japan) and were identified on the basis of morphological evaluation by Natalya N. Kirichuk from the G.B. Elyakov Pacific Institute of Bioorganic Chemistry (PIBOC). Strains are stored at the Collection of Marine Microorganisms, PIBOC, Vladivostok, Russia with the codes KMM 4663 and KMM 4645, respectively. The fungi were grown stationary at 22 °C for 21 days in 20 Erlenmeyer flasks (500 mL) (for each strain), each flask containing 20 g of rice, 20 mg of yeast extract, 10 mg of KH_2_PO_4_, and 40 mL of natural sea water (Marine Experimental Station of G.B. Elyakov Pacific Institute of Bioorganic Chemistry, Troitsa (Trinity) Bay, Sea of Japan).

### 3.3. Extraction

At the end of the incubation period, the mycelia and medium were homogenized and extracted with EtOAc (2 L). The extract of each fungus was concentrated to dryness. The residue was dissolved in 20% MeOH–H_2_O (1 L) and was extracted with *n*-hexane (0.2 L × 3) and EtOAc (0.2 L × 3). After evaporation of the EtOAc layer, the residual materials (1.5 g, *P. thomii* and 1.3 g, *P. lividum*) were passed over silica columns (4 × 20 cm), which were eluted first with *n*-hexane (1 L) followed by a step gradient from 5% to 100% EtOAc in *n*-hexane (total volume 7 L). Fractions of 200 mL were collected and combined on the basis of TLC (Si gel, toluene–isopropanol 6:1, v/v).

### 3.4. Isolation Metabolites from P. thomii

The *n*-hexane–EtOAc (3:2, 1.5 L) eluate (100 mg) was purified by RP HPLC on a Supelco Discovery C-18 column eluting with MeOH–H_2_O (40:60) to yield **1** (10 mg). The EtOAc (1.0 L) eluate (60 mg) gave **5** (5 mg) eluting with MeOH–H_2_O (40:60).

### 3.5. Isolation Metabolites from P. lividum

The *n*-hexane–EtOAc (5:1, 1.4 L) eluate (250 mg) was purified by RP HPLC on a Supelco Discovery C-18 column eluting with MeOH–H_2_O (65:35) to yield **3** (2.1 mg), **4** (5.8 mg) and MeOH–H_2_O (50:50) to yield **2** (3.4 mg) and **7** (3.5 mg). The EtOAc (1.0 L) eluate (64 mg) gave **5** (3.4 mg) and **6** (4.5 mg) after purification by HPLC (MeOH–H_2_O, 40:60).

### 3.6. Physicochemical and Spectroscopic Data of **1**–**7**

Sargassopenilline A (**1**): Amorphous solid; [α]^20^_D_ −45 (*c* 0.10, MeOH); UV (MeOH) λ_max_ (log ε) 235 (3.22), 282 (3.07) nm; CD (*c* 0.6 mg/mL, MeOH) λ_max_ (Δε) 233(+0.06), 247 (−0.04), 282 (+0.04), 350 (+0.01) nm; ^1^H and ^13^C NMR data, see Table 1 and Table 2; HRESIMS *m/z* 319.1157 [M + Na]^+^ (calcd for C_15_H_20_O_6_Na, 319.1152).

Sargassopenilline B (**2**): Amorphous solid; [α]^20^_D_ −137 (*c* 0.09, MeOH); UV (MeOH) λ_max_ (log ε) 219 (3.77), 284 (3.25) nm; CD (*c* 0.2 mg/mL, MeOH) λ_max_ (Δε) 244 (−0.86), 274 (−0.35), 308 (+0.20), nm; ^1^H and ^13^C NMR data, see Table 1 and Table 2; HRESIMS *m/z* 403.1366 [M+Na]^+^ (calcd for C_19_H_24_O_8_Na, 403.1363).

Sargassopenilline C (**3**): Amorphous solid; [α]^20^_D_ −84 (*c* 0.17, MeOH); UV (MeOH) λ_max_ (log ε) 208 (4.25), 250 (3.76), 283 (3.82) nm; CD (*c* 0.025 mg/mL, MeOH) λ_max_ (Δε) 240 (−2.46), 318 (+0.38), 380 (−0.27), nm; IR (CHCl_3_) ν_max_ 3610, 2928, 2855, 1742, 1690, 1648, 1603, 1456, 1373, 1253, 1164, 1092, 1067 cm^−1^; ^1^H and ^13^C NMR data, see Table 1 and Table 2; HRESIMS *m/z* 477.1365 [M + Na]^+^ (calcd for C_21_H_26_O_11_Na, 477.1367).

Sargassopenilline D (**4**): Amorphous solid; [α]^20^_D_ −37 (*c* 0.07, MeOH); UV (MeOH) λ_max_ (log ε) 196 (3.29), 268 (2.91) nm; CD (*c* 0.17 mg/mL, MeOH) λ_max_ (Δε) 198 (−2.51), 210 (−2.15), 254 (+1.51), 282 (−8.43), 340 (−0.35) nm; ^1^H and ^13^C NMR data, see Table 1 and Table 2; HRESIMS *m/z* 417.1157 [M + Na]^+^ (calcd for C_19_H_22_O_9_Na, 417.1156), 393.1207 [M − H]^+^ (calcd for C_19_H_21_O_9_, 393.1191).

Sargassopenilline E (**5**): Amorphous solid; [α]^20^_D_ −107 (*c* 0.16, MeOH); UV (MeOH) λ_max_ (log ε) 242 (3.01) nm; CD (*c* 0.33 mg/mL, MeOH) λ_max_ (Δε) 216 (+7.57), 248 (−5.07), 310 (+0.29), 350 (−0.05) nm; ^1^H and ^13^C NMR data, see Table 1 and Table 2; HRESIMS *m/z* 321.1296 [M + Na]^+^ (calcd for C_15_H_22_O_6_Na, 321.1309), 299.1483 [M + H]^+^ (calcd for C_15_H_23_O_6_, 299.1489).

Sargassopenilline F (**6**): Amorphous solid; [α]^20^_D_ −45 (*c* 0.01, MeOH); UV (MeOH) λ_max_ (log ε) 206 (3.05), 221 (3.02), 247 (2.70), 268 (2.63) nm; CD (*c* 0.18 mg/mL, MeOH) λ_max_ (Δε) 220 (+4.60), 248 (−6.50), 328 (+0.39) nm; ^1^H and ^13^C NMR data, see Table 1 and Table 2; HRESIMS *m/z* 379.1379 [M + Na]^+^ (calcd for C_17_H_24_O_8_Na, 379.1363), 357.1548 [M + H]^+^ (calcd for C_17_H_25_O_8_, 357.1544).

Sargassopenilline G (**7**): Amorphous solid; [α]^20^_D_ −158 (*c* 0.09, MeOH); UV (MeOH) λ_max_ (log ε) 215 (3.69) nm; CD (*c* 0.2 mg/mL, MeOH) λ_max_ (Δε) 242 (−0.64), 306 (−0.39), 347 (+0.14), nm; ^1^H and ^13^C NMR data, see Table 3; HRESIMS *m/z* 391.1367 [M + Na]^+^ (calcd for C_18_H_24_O_8_Na, 391.1363).

### 3.7. Preparation of (S)-MTPA and (R)-MTPA Esters of **1**

4-Dimethylaminopyridine (a few crystals) and (*R*)-MTPACl (20 μL) were added to a solution of the **1** (4.0 mg) in pyridine and stirred at room temperature (25 °C) for 24 h. After evaporation of the solvent, the residue was passed through a silica gel column (7% EtOAc–hexane) to generate the (*S*)-MTPA ester (**1a**). The (*R*)-MTPA ester (**1b**) was prepared in a similar manner using (*S*)-MTPACl. ^1^H and COSY data, Supplementary Information S7–S10; ESIMS of **1a** *m/z* 729.39 [M + H]^+^ and of **1b** *m/z* 729.35 [M + H]^+^.

### 3.8. Preparation of (S)-MTPA and (R)-MTPA Esters of **5**

4-Dimethylaminopyridine (a few crystals) and (*R*)-MTPACl (20 μL) were added to a solution of the **5** (4.0 mg) in pyridine and stirred at room temperature (25 °C) for 24 h. After evaporation of the solvent, the residue was passed through a silica gel column (7% EtOAc–hexane) to generate the (*S*)-MTPA ester (**5a**). The (*R*)-MTPA ester (**5b**) was prepared in a similar manner using (*S*)-MTPACl. ^1^H and COSY data, Supplementary Information S35–S38; ESIMS of **5a** *m/z* 731.42 [M + H]^+^ and of **5b** *m/z* 7315.44 [M + H]^+^.

### 3.9. Preparation of (S)-MTPA and (R)-MTPA Esters of **6**

4-Dimethylaminopyridine (a few crystals) and (*R*)-MTPACl (20 μL) were added to a solution of the **6** (3.0 mg) in pyridine and stirred at room temperature (25 °C) for 24 h. After evaporation of the solvent, the residue was passed through a silica gel column (15% EtOAc–hexane) to generate the (*S*)-MTPA ester (**6a**). The (*R*)-MTPA ester (**6b**) was prepared in a similar manner using (*S*)-MTPACl. ^1^H and COSY data, Supplementary Information S51–S54; ESIMS of **6a** *m/z* 573.21 [M + H]^+^ and of **6b** *m/z* 573.30 [M + H]^+^.

The spectra of compounds **1**–**7** are all given in the Supplementary Information.

### 3.10. Cytotoxicity Assay

The effect of the compounds on the cells viability was evaluated using the MTS test, which is based on the reduction of MTS into its formazan product by alive cells [14,15,16]. Cytotoxicity towards CD-I mouse splenocytes was determined according to Freshney [17]. Hemolytic activity towards CD-I mouse erythrocytes was determined as previously described [18].

### 3.11. Determination of the Effects of Compounds on the Basal Transcriptional Activity of AP-1

The effects of the compounds on the basal transcriptional activities of AP-1 were evaluated using the JB6 Cl41 cell line stably expressing a luciferase reporter gene controlled by an AP-1-DNA binding sequence [9]. The experiments were performed as previously reported [16] with slight modifications.

### 3.12. Macrophage Adhesion Test

Mice were sacrificed by cervical dislocation. Peritoneal macrophages were isolated using standard procedures. For this purpose, 3 mL of PBS (pH 7.4) was immediately injected into the peritoneal cavity and the body intensively palpated for 1–2 min. Then, the peritoneal fluid was aspirated with a syringe and transferred to Petri dishes. Petri dishes with the fluid were incubated at 37 °C for 1–2 h to facilitate attachment of peritoneal macrophages to the dish. Then, a cell monolayer was triply flushed with PBS (pH 7.4) to delete attendant lymphocytes, fibroblasts and erythrocytes. Subsequently, macrophages were removed from the surface of the dishes with a scraper and flow of a saline solution, and then placed on an ice bath until use. The working concentration of cells was usually 1–2 × 10^6^ cells/mL. The number of adhered cells was estimated according to standard method [19] with some modifications.

Twenty milliliters of test compound solutions (at non-toxic concentration 10 μM) and 200 mL of macrophage suspension were added to 96-well flat-bottom plates. After incubation for 2 h at 37 °C, cells were fixed with 70% ethanol solution (100 mL per well) for 15 min at RT. Then, cells were washed with 200 mL of PBS, and a 0.05% solution of Trypan blue (100 mL per well) was added to each well and further incubated at 37 °C for 15 min. Next, media was removed and the cells were gently washed with cold PBS (3 × 200 mL). The cells were then lysed with 50 mL of 1% SDS for at least 4 h and gently triturated. Finally, the SDS/trypan blue solution absorbance was detected spectrophotometrically at 590 nm using a plate reader. The percentage of adhered cells compared to the control level of Trypan blue absorbance was calculated. All samples were assayed in triplicates.

### 3.13. Radical Scavenging Activity against DPPH

The experiments were performed as previously reported [20]. Ascorbic acid was used as the positive control and showed an IC_50_ value 21.3 μM.

## 4. Conclusions

Seven new polyketides, named sargassopenillines A–G (**1**–**7**) have been isolated from the lipophilic extracts of the marine-derived fungi *Penicillium thomii* and *Penicillium lividum*. Sargassopenillines A (**1**) and B (**2**) are new members of the rare class of natural products that contain an aryl ring fused to the 6,6-spiroketal part [7,21,22]. Notable features of sargassopenillines D (**4**) and G (**7**) are the presence of *p*-benzoquinone and cyclopentenone moieties as their core skeletons. Sargassopenilline C (**3**) inhibited the transcriptional activity of the oncogenic nuclear factor AP-1 with an IC_50_ value of 15 µM.

## Supplementary Files

Supplementary File 1
