# Supplementary material for: Sargassopenillines A–G, 6,6-Spiroketals from the Alga-Derived Fungi Penicillium thomii and Penicillium lividum
Source: Mar Drugs. 2014 Dec 9;12(12):5930–43. doi: 10.3390/md12125930 (PMC4278210; doi:10.3390/md12125930)
Supplement: Supplementary File 1 [file marinedrugs-12-05930-s001.pdf]

Supplementary Information

Figure S1.^{1}H NMR (500 MHz, CD_3OD) spectrum of **1**
Figure S2. ^{1}H-^{1}H COSY (500 MHz, CD_3OD) spectrum of **1**
Figure S3. ^{13}C NMR (125 MHz, CD_3OD) spectrum of **1**
Figure S4. HSQC (500 MHz, CD_3OD) spectrum of **1**
Figure S5. HMBC (500 MHz, CD_3OD) spectrum of **1**
Figure S6. NOESY (500 MHz, CD_3OD) spectrum of **1**
Figure S7. ^{1}H NMR (700 MHz, CD_3OD) spectrum of (*S*)-MTPA ester of **1a**
Figure S8. ^{1}H-^{1}H COSY (700 MHz, CD_3OD) spectrum of (*S*)-MTPA ester of **1a**
Figure S9. ^{1}H NMR (700 MHz, CD_3OD) spectrum of (*R*)-MTPA ester of **1b**
Figure S10. ^{1}H-^{1}H COSY (700 MHz, CD_3OD) spectrum of (*S*)-MTPA ester of **1b**
Figure S11. ^{1}H NMR (700 MHz, $CDCl_3$) spectrum of **2**
Figure S12. ^{1}H-^{1}H COSY (700 MHz, $CDCl_3$) spectrum of **2**
Figure S13. ^{13}C NMR (176 MHz, $CDCl_3$) spectrum of **2**
Figure S14. HSQC (700 MHz, $CDCl_3$) spectrum of **2**
Figure S15. HMBC (700 MHz, $CDCl_3$) spectrum of **2**
Figure S16. NOESY (700 MHz, $CDCl_3$) spectrum of **2**
Figure S17. ^{1}H NMR (500 MHz, $CDCl_3$) spectrum of **3**
Figure S18. ^{1}H-^{1}H COSY (500 MHz, $CDCl_3$) spectrum of **3**
Figure S19. ^{13}C NMR (125 MHz, $CDCl_3$) spectrum of **3**
Figure S20. HSQC (500 MHz, $CDCl_3$) spectrum of **3**
Figure S21. HMBC (500 MHz, $CDCl_3$) spectrum of **3**
Figure S22. NOESY (500 MHz, $CDCl_3$) spectrum of **3**
Figure S23. ^{1}H NMR (700 MHz, $CDCl_3$) spectrum of **4**
Figure S24. ^{1}H-^{1}H COSY (700 MHz, $CDCl_3$ spectrum of **4**
Figure S25. ^{13}C NMR (176 MHz, $CDCl_3$) spectra of **4**
Figure S26. HSQC (700 MHz, $CDCl_3$) spectrum of **4**
Figure S27. HMBC (700 MHz, $CDCl_3$) spectrum of **4**
Figure S28. NOESY (700 MHz, $CDCl_3$) spectrum of **4**
Figure S29. ^{1}H NMR (700 MHz, $CDCl_3$) spectrum of **5**
Figure S30. ^{1}H-^{1}H COSY (700 MHz, $CDCl_3$ spectrum of **5**
Figure S31. ^{13}C NMR (176 MHz, $CDCl_3$) spectra of **5**
Figure S32. HSQC (700 MHz, $CDCl_3$) spectrum of **5**
Figure S33. HMBC (700 MHz, $CDCl_3$) spectrum of **5**
Figure S34. NOESY (700 MHz, $CDCl_3$) spectrum of **5**
Figure S35. ^{1}H NMR (500 MHz, $CDCl_3$) spectrum of (*S*)-MTPA ester of **5a**
Figure S36. ^{1}H-^{1}H COSY (500 MHz, $CDCl_3$) spectrum of (*S*)-MTPA ester of **5a**
Figure S37. ^{1}H NMR (500 MHz, $CDCl_3$) spectrum of (*R*)-MTPA ester of **5b**
Figure S38. ^{1}H-^{1}H COSY (500 MHz, $CDCl_3$) spectrum of (*S*)-MTPA ester of **5b**
Figure S39. ^{1}H NMR (700 MHz, DMSO) spectrum of **5**
Figure S40. ^{1}H-^{1}H COSY (700 MHz, DMSO) spectrum of **5**
Figure S41. ^{13}C NMR (176 MHz, DMSO) spectra of **5**

Figure S42. HSQC (700 MHz, DMSO) spectrum of **5**
Figure S43. HMBC (700 MHz, DMSO) spectrum of **5**
Figure S44. NOESY (700 MHz, DMSO) spectrum of **5**
Figure S45. ^{1}H NMR (500 MHz, $CDCl_3$) spectrum of **6**
Figure S46. ^{1}H-^{1}H COSY (500 MHz, $CDCl_3$) spectrum of **6**
Figure S47. ^{13}C NMR (125 MHz, $CDCl_3$) spectrum of **6**
Figure S48. HSQC (500 MHz, $CDCl_3$) spectrum of **6**
Figure S49. HMBC (500 MHz, $CDCl_3$) spectrum of **6**
Figure S50. NOESY (500 MHz, $CDCl_3$) spectrum of **6**
Figure S51. ^{1}H NMR (500 MHz, $CDCl_3$) spectrum of (*S*)-MTPA ester of **6a**
Figure S52. ^{1}H-^{1}H COSY (500 MHz, $CDCl_3$) spectrum of (*S*)-MTPA ester of **6a**
Figure S53. ^{1}H NMR (500 MHz, $CDCl_3$) spectrum of (*R*)-MTPA ester of **6b**
Figure S54. ^{1}H-^{1}H COSY (500 MHz, $CDCl_3$) spectrum of (*S*)-MTPA ester of **6b**
Figure S55. ^{1}H NMR (700 MHz, $CDCl_3$) spectrum of **7**
Figure S56. ^{1}H-^{1}H COSY (700 MHz, $CDCl_3$) spectrum of **7**
Figure S57. ^{13}C NMR (176 MHz, $CDCl_3$) spectra of **7**
Figure S58. HSQC (700 MHz, $CDCl_3$) spectrum of **7**
Figure S59. HMBC (700 MHz, $CDCl_3$) spectrum of **7**
Figure S60. NOESY (700 MHz, $CDCl_3$) spectrum of **7**

Figure S1. ^{1}H NMR (500 MHz, CD_3OD) spectrum of **1**.

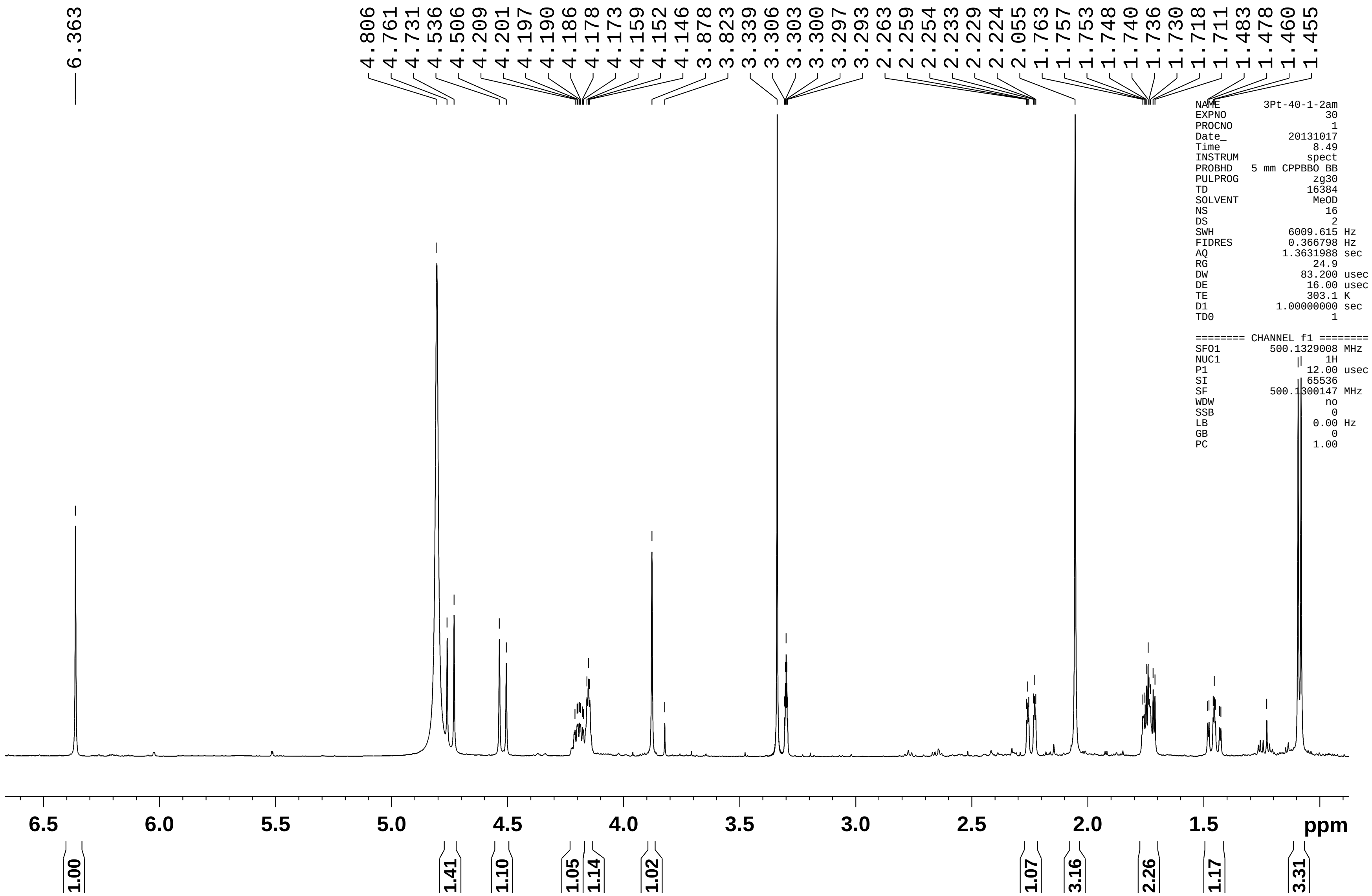

Figure S2. ^{1}H-^{1}H COSY (500 MHz, CD_3OD) spectrum of **1**.

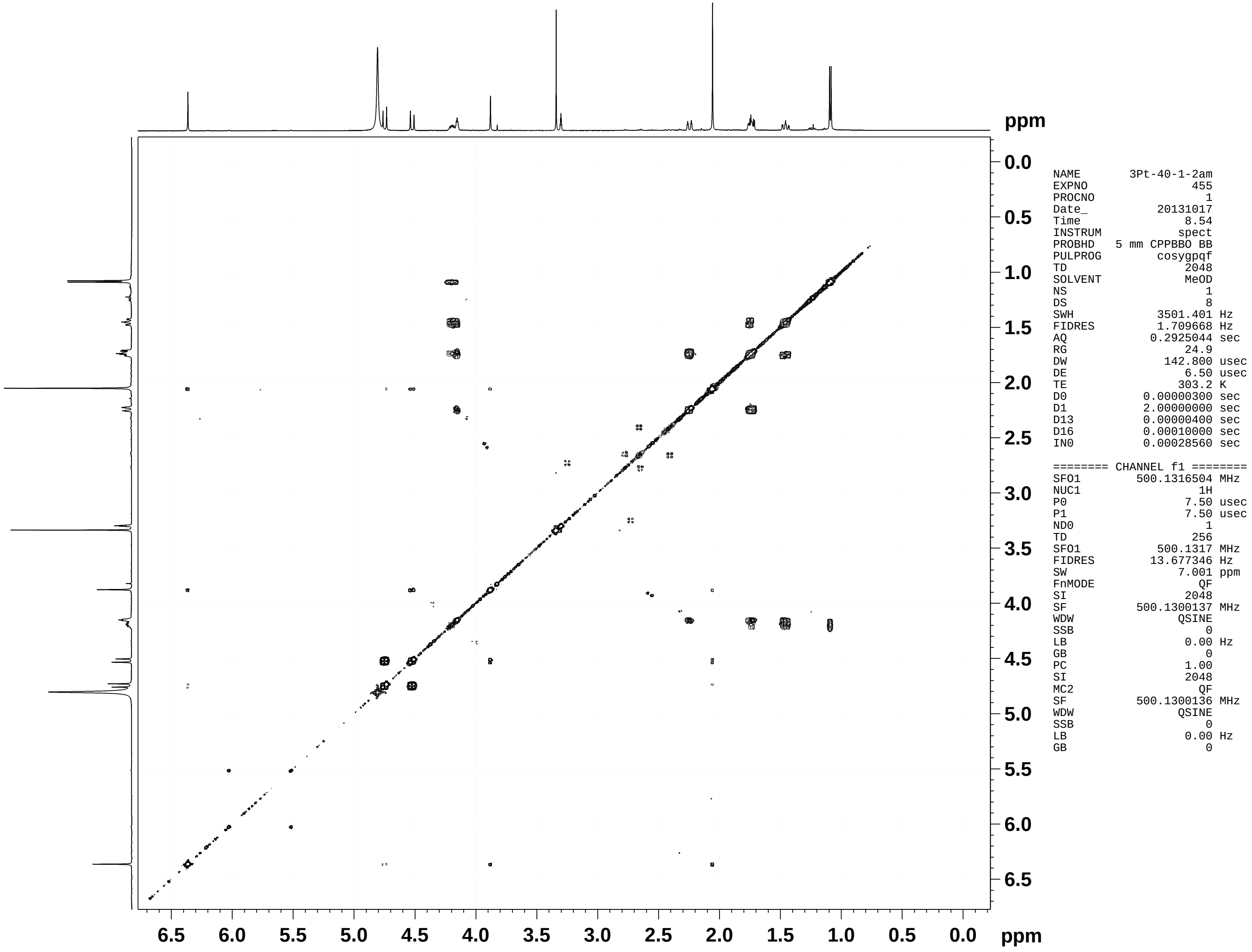

Figure S3. ^{13}C NMR (125 MHz, CD_3OD) spectrum of **1**.

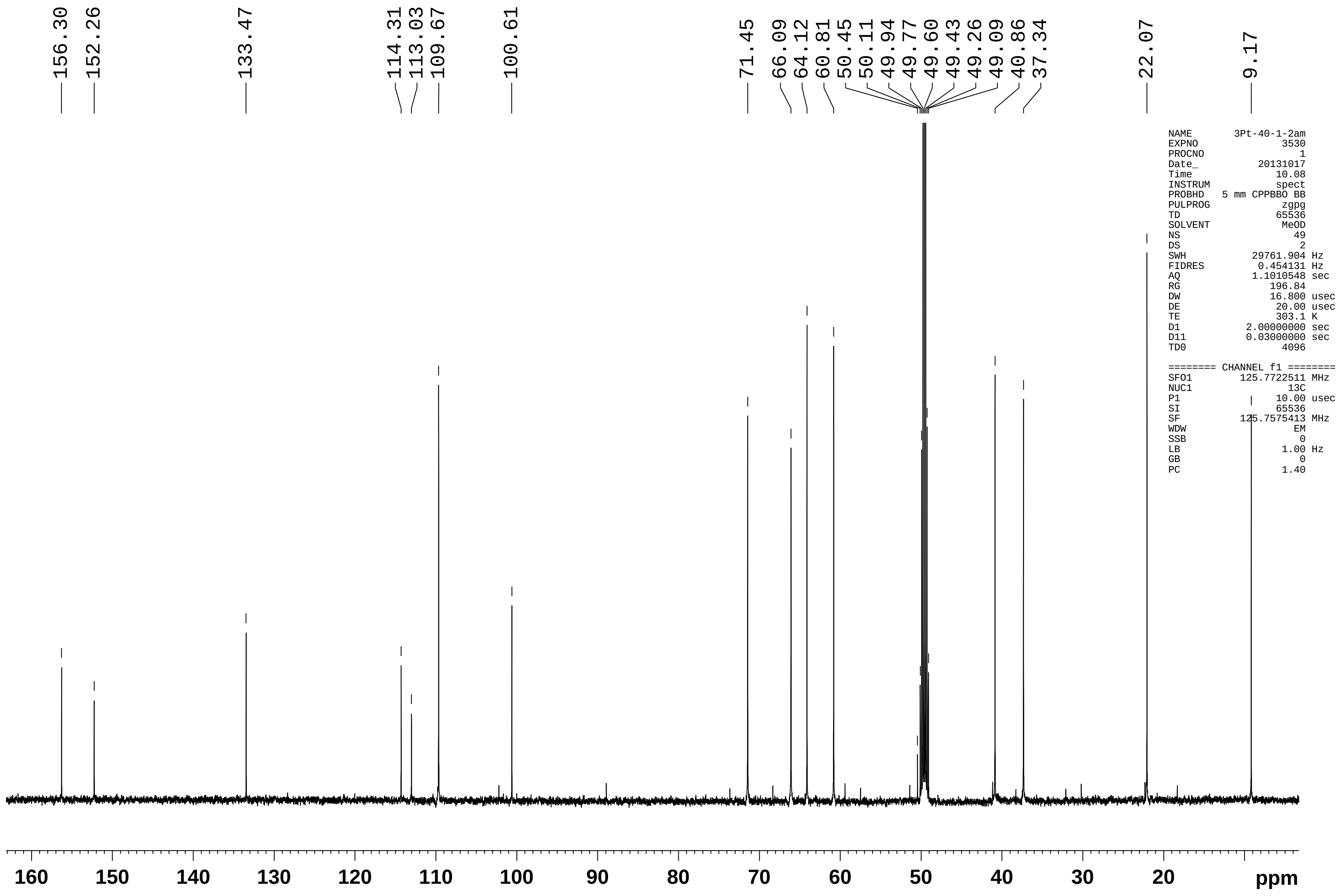

Figure S4. HSQC (500 MHz, CD_3OD) spectrum of **1**.

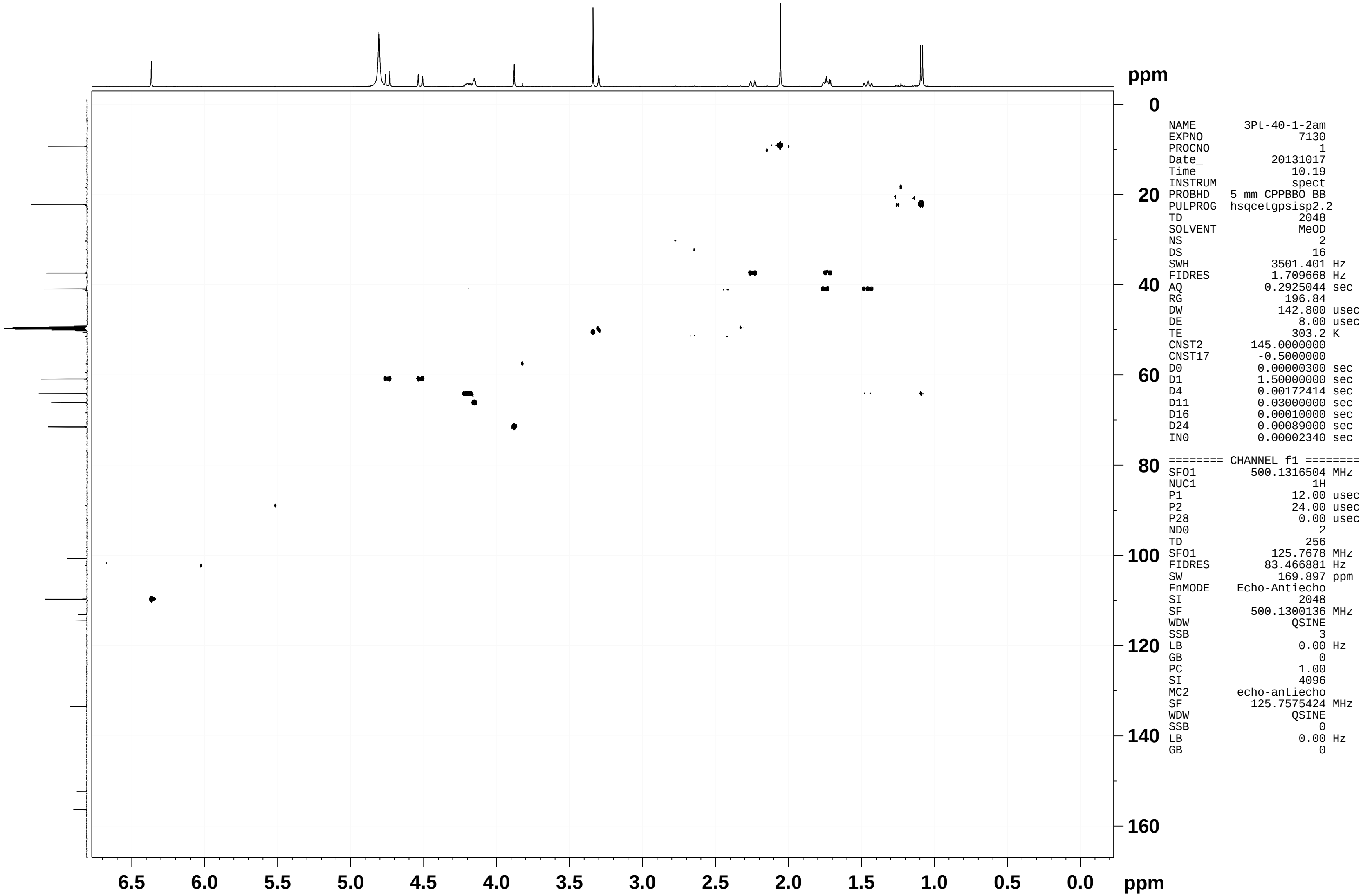

Figure S5. HMBC (500 MHz, CD_3OD) spectrum of **1**.

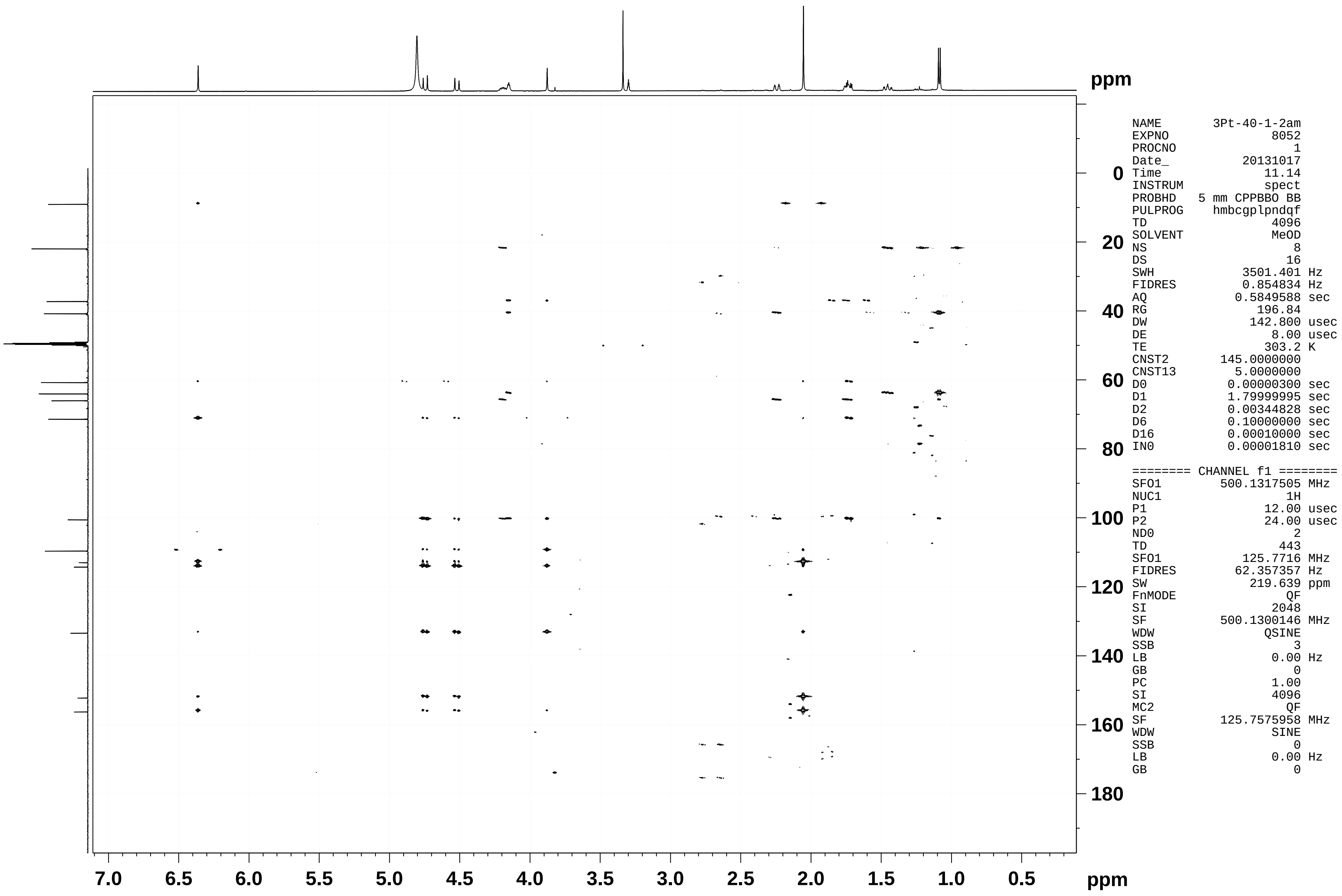

Figure S6. NOESY (500 MHz, CD_3OD) spectrum of **1**.

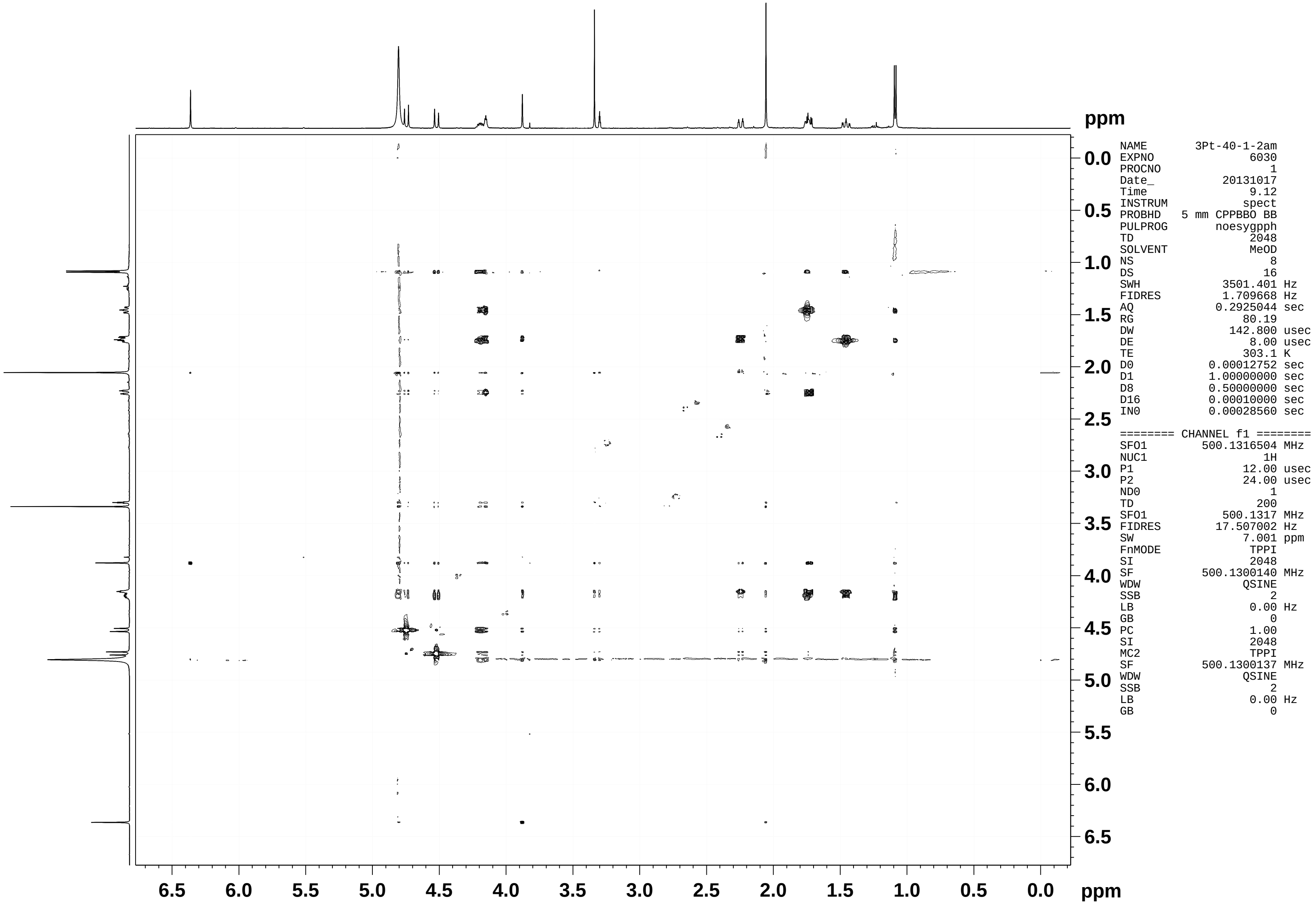

Figure S7. ^{1}H NMR (700 MHz, CD_3OD) spectrum of (*S*)-MTPA ester of **1a**.

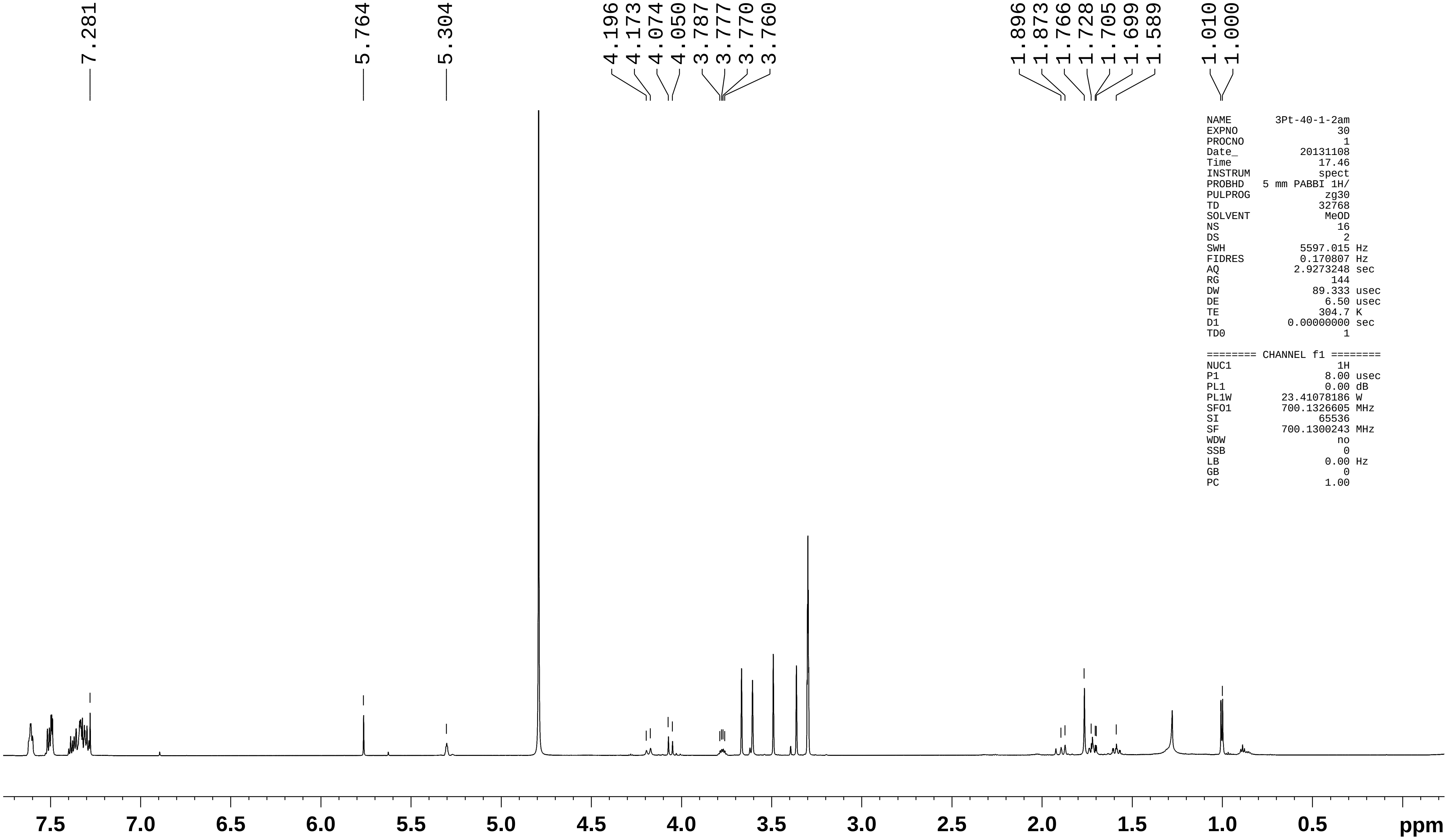

Figure S8. ^{1}H-^{1}H COSY (700 MHz, CD_3OD) spectrum of (*S*)-MTPA ester of **1a**.

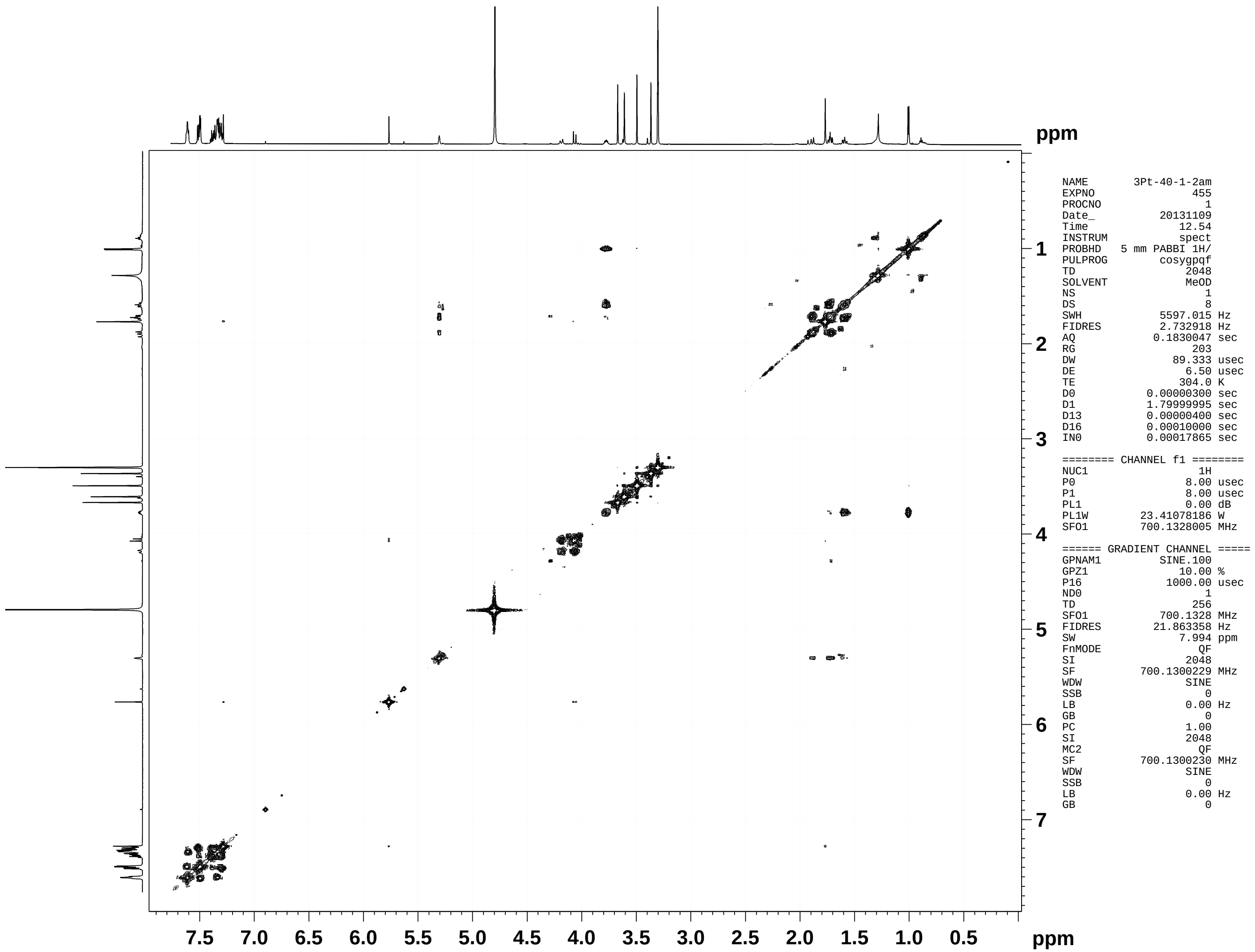

Figure S9. ^{1}H NMR (700 MHz, CD_3OD) spectrum of (*R*)-MTPA ester of **1b**.

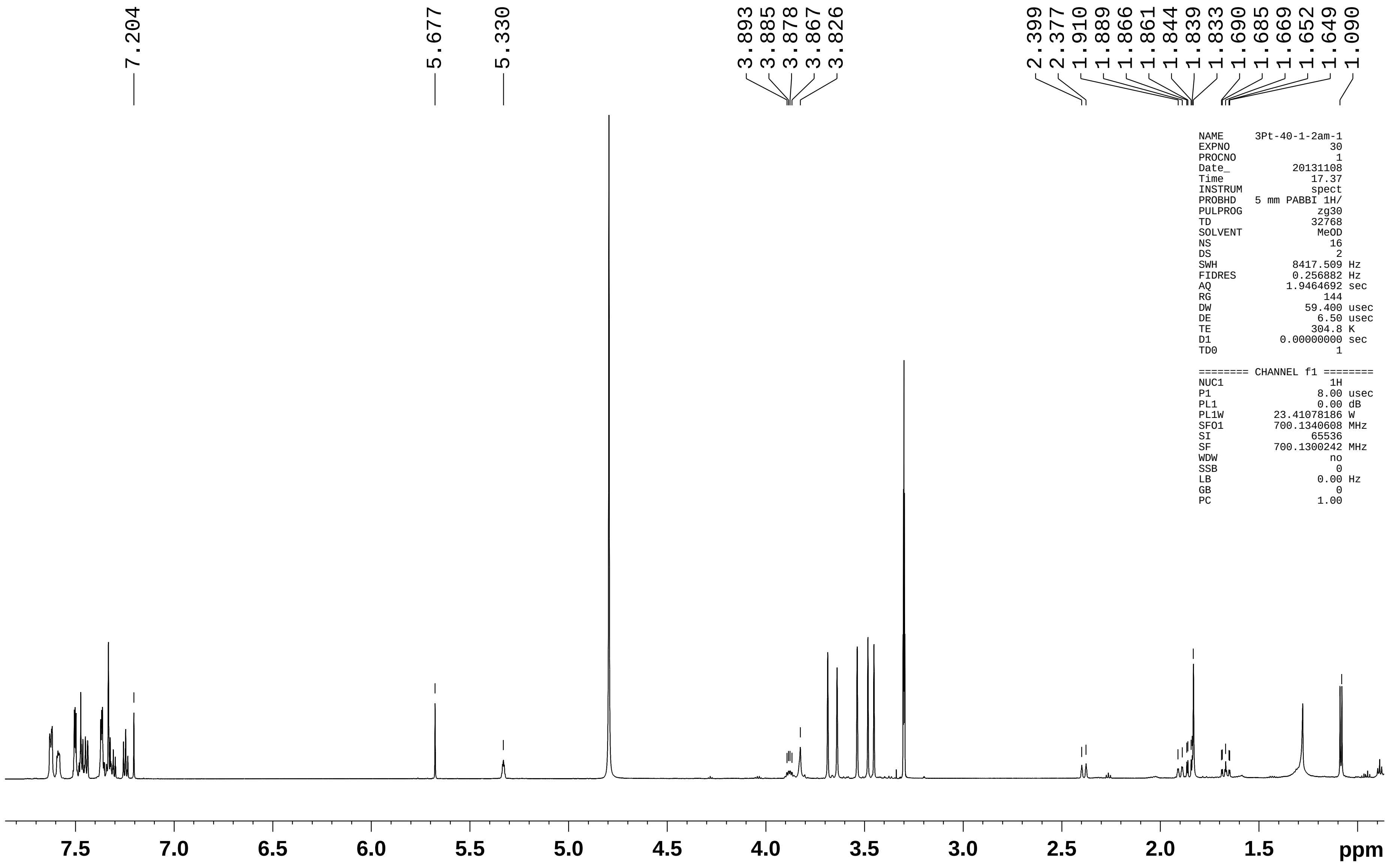

Figure S10. ^{1}H-^{1}H COSY (700 MHz, CD_3OD) spectrum of (*R*)-MTPA ester of **1b**.

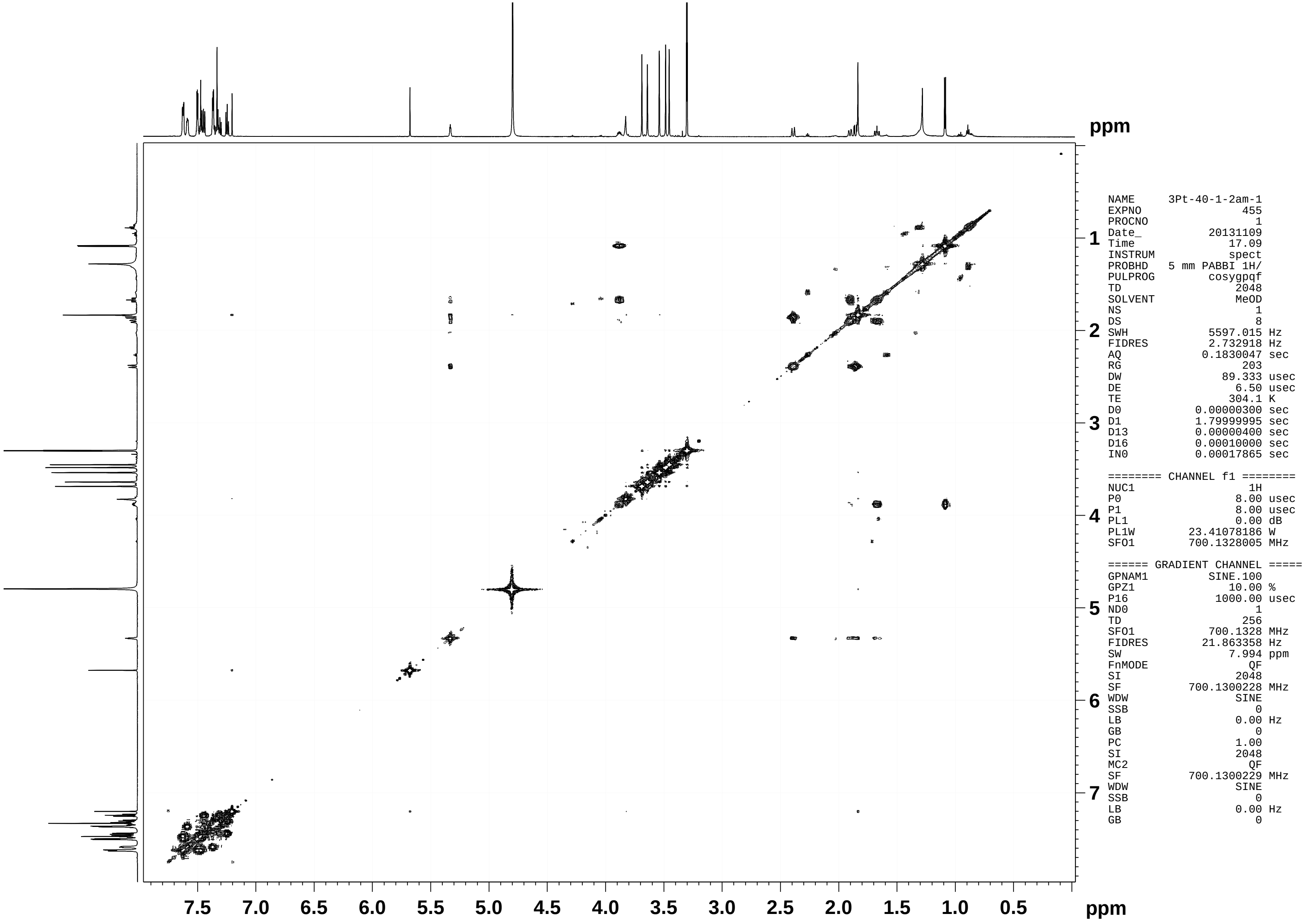

Figure S11. ^{1}H NMR (700 MHz, $CDCl_3$) spectrum of **2**.

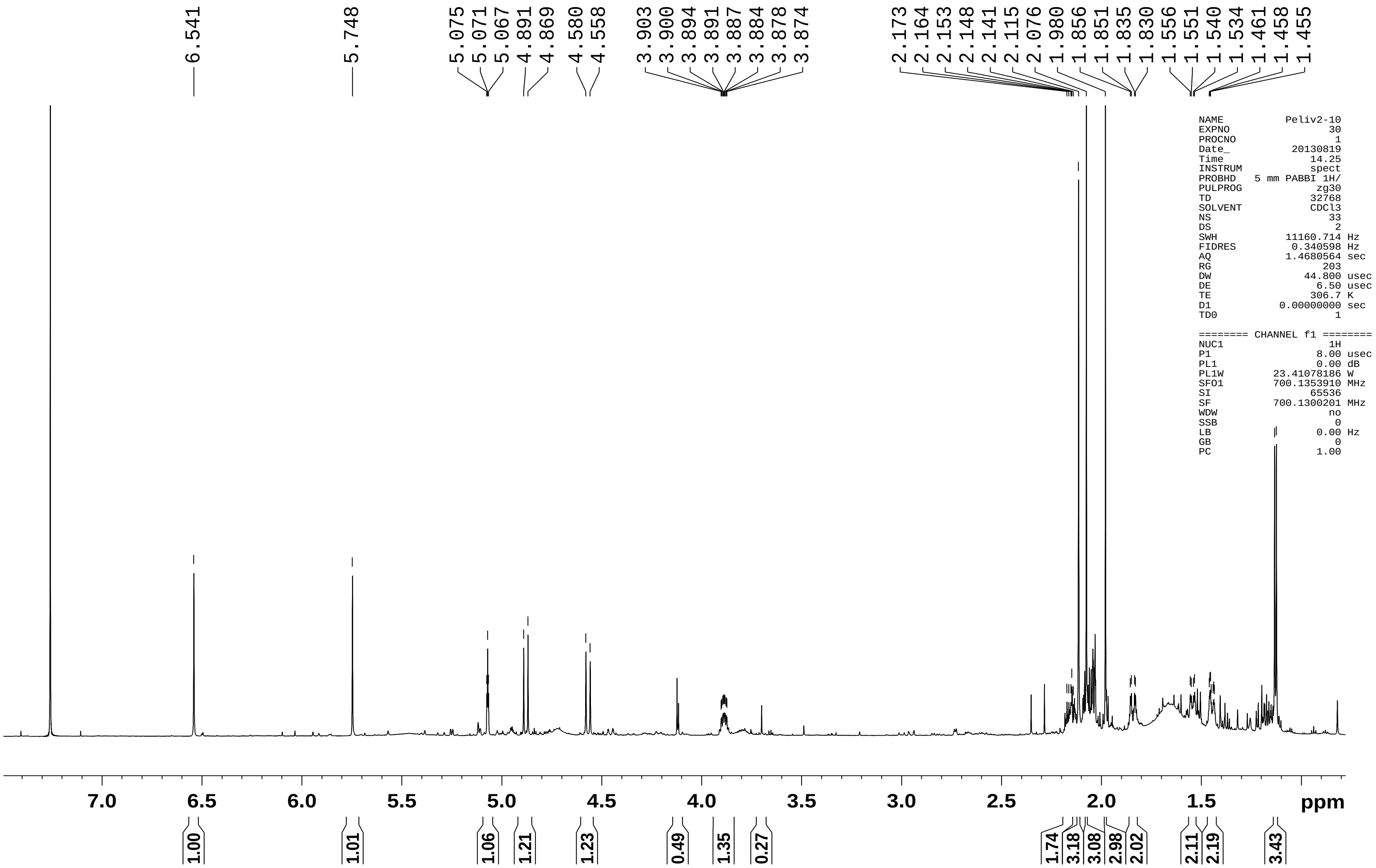

Figure S12. ^{1}H-^{1}H COSY (700 MHz, $CDCl_3$) spectrum of **2**.

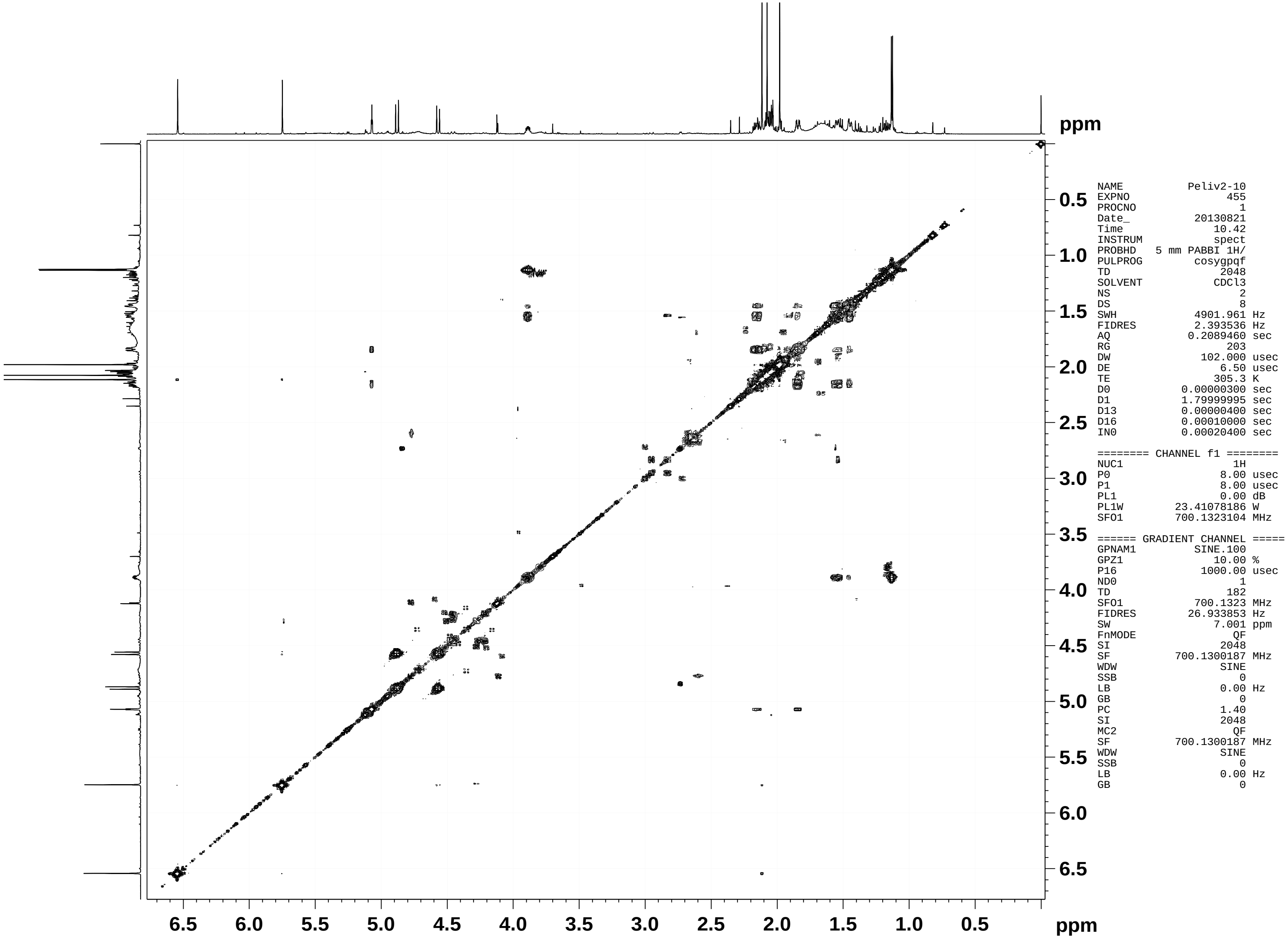

Figure S13. ^{13}C NMR (176 MHz, $CDCl_3$) spectrum of **2**.

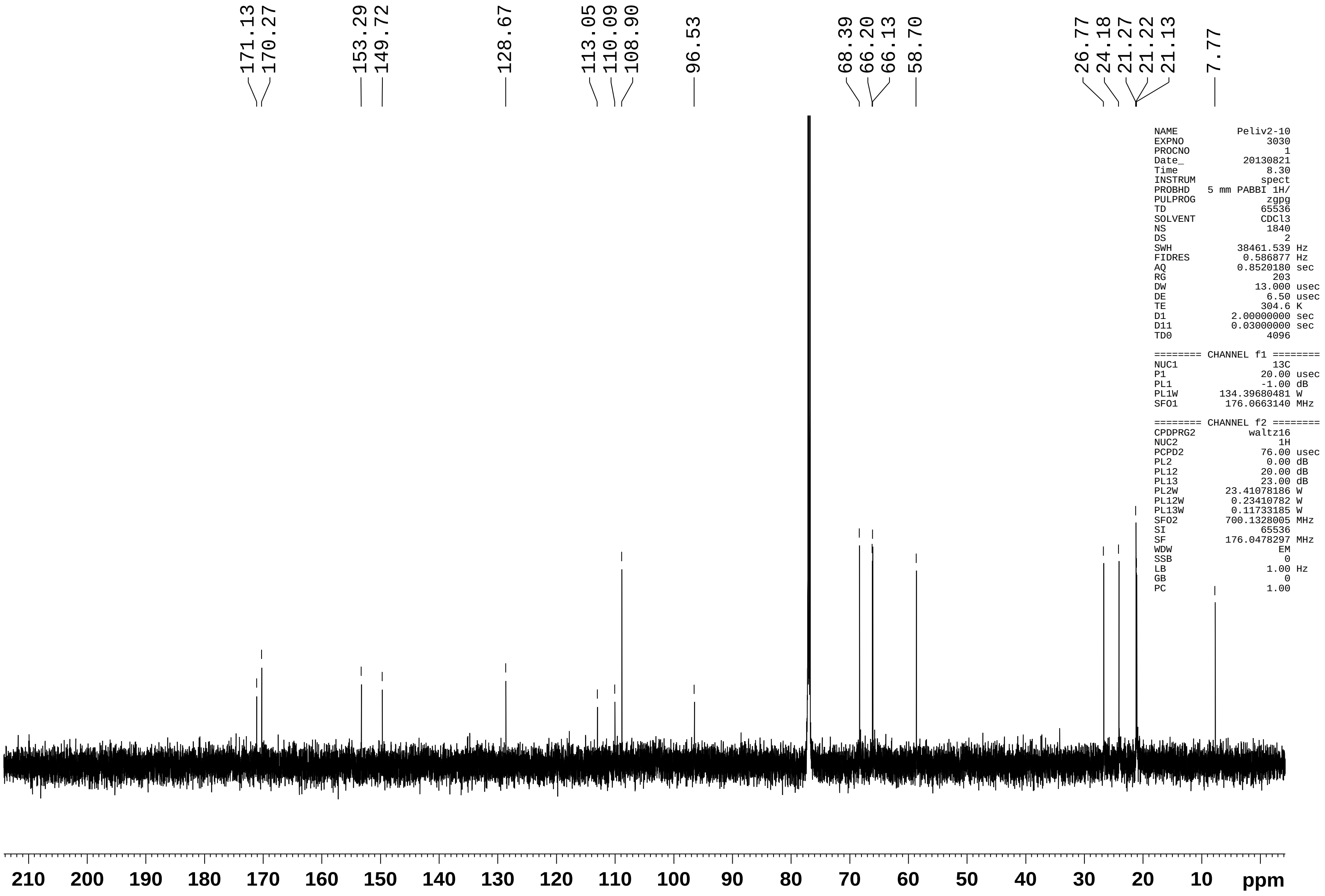

Figure S14. HSQC (700 MHz, $CDCl_3$) spectrum of **2**.

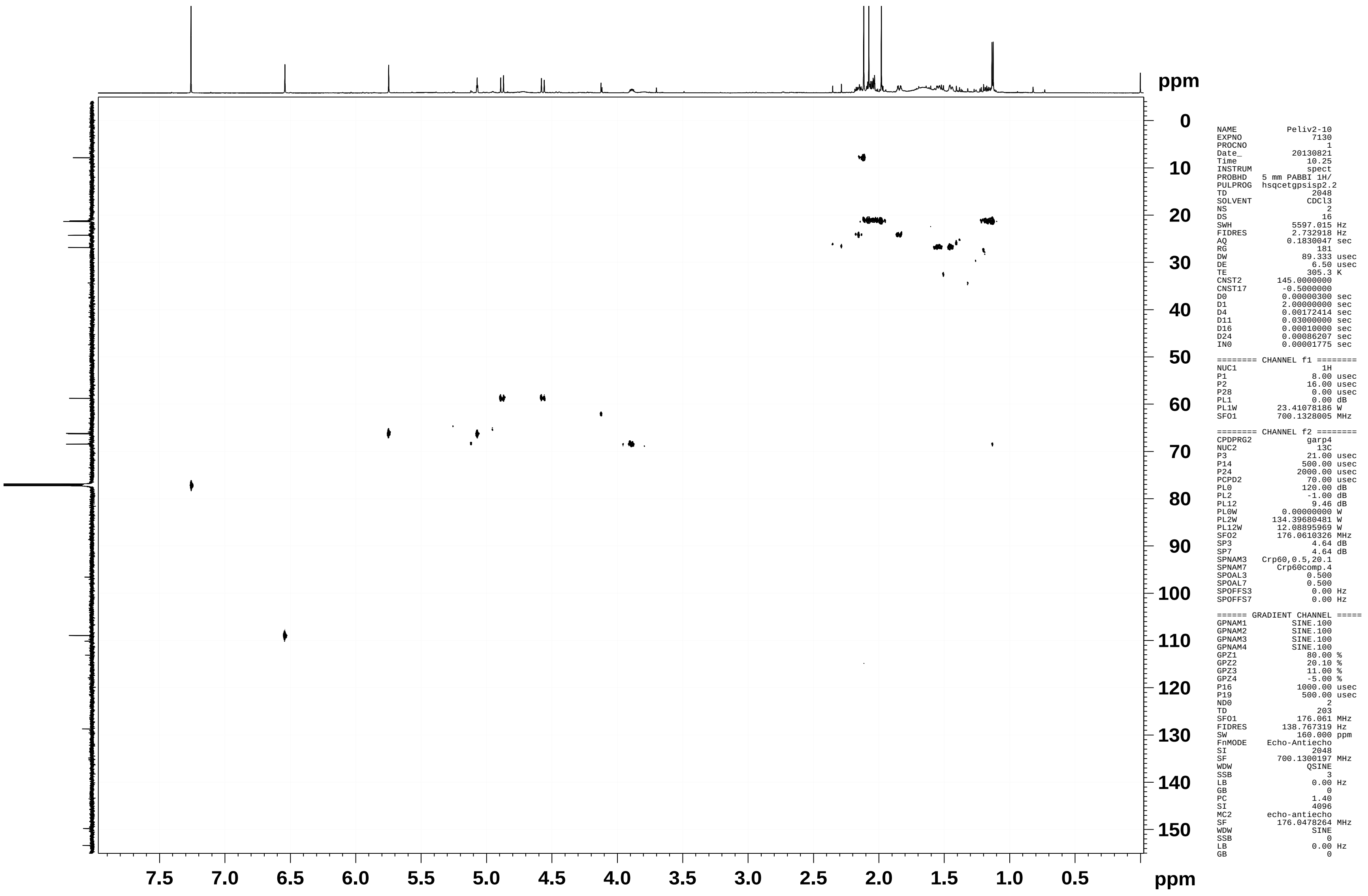

Figure S15. HMBC (700 MHz, $CDCl_3$) spectrum of **2**.

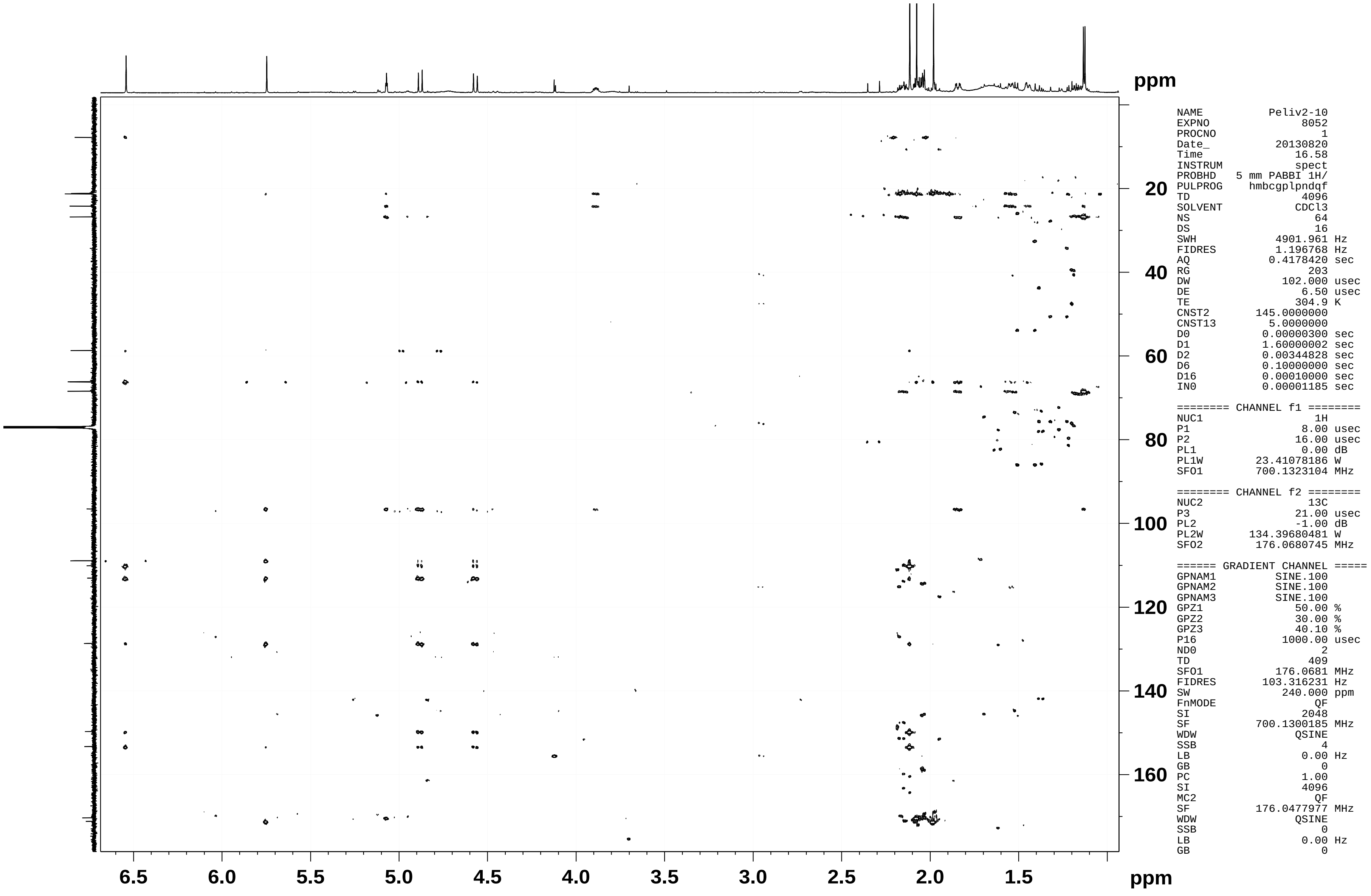

Figure S16. NOESY (700 MHz, $CDCl_3$) spectrum of **2**.

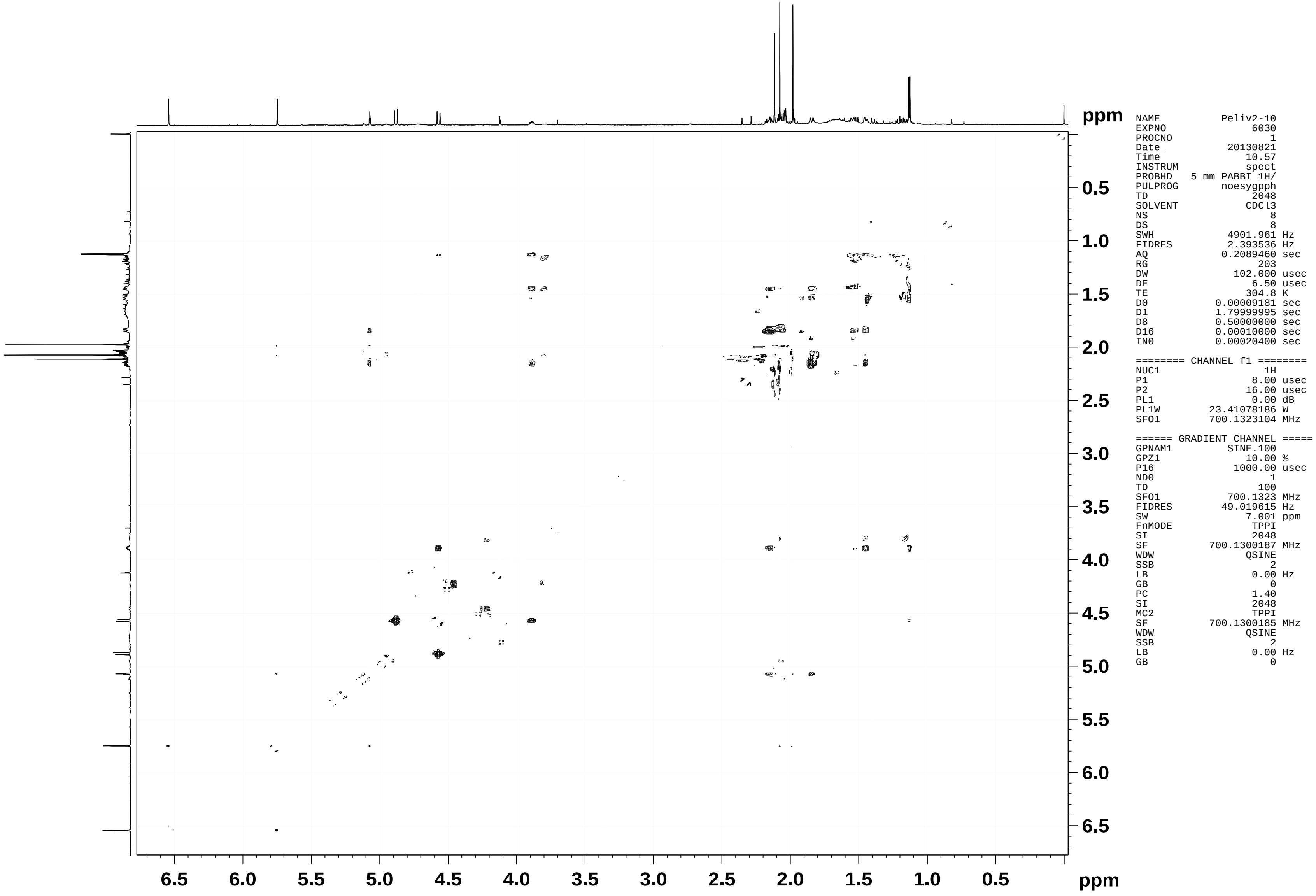

Figure S17. ^{1}H NMR (500 MHz, $CDCl_3$) spectrum of **3**.

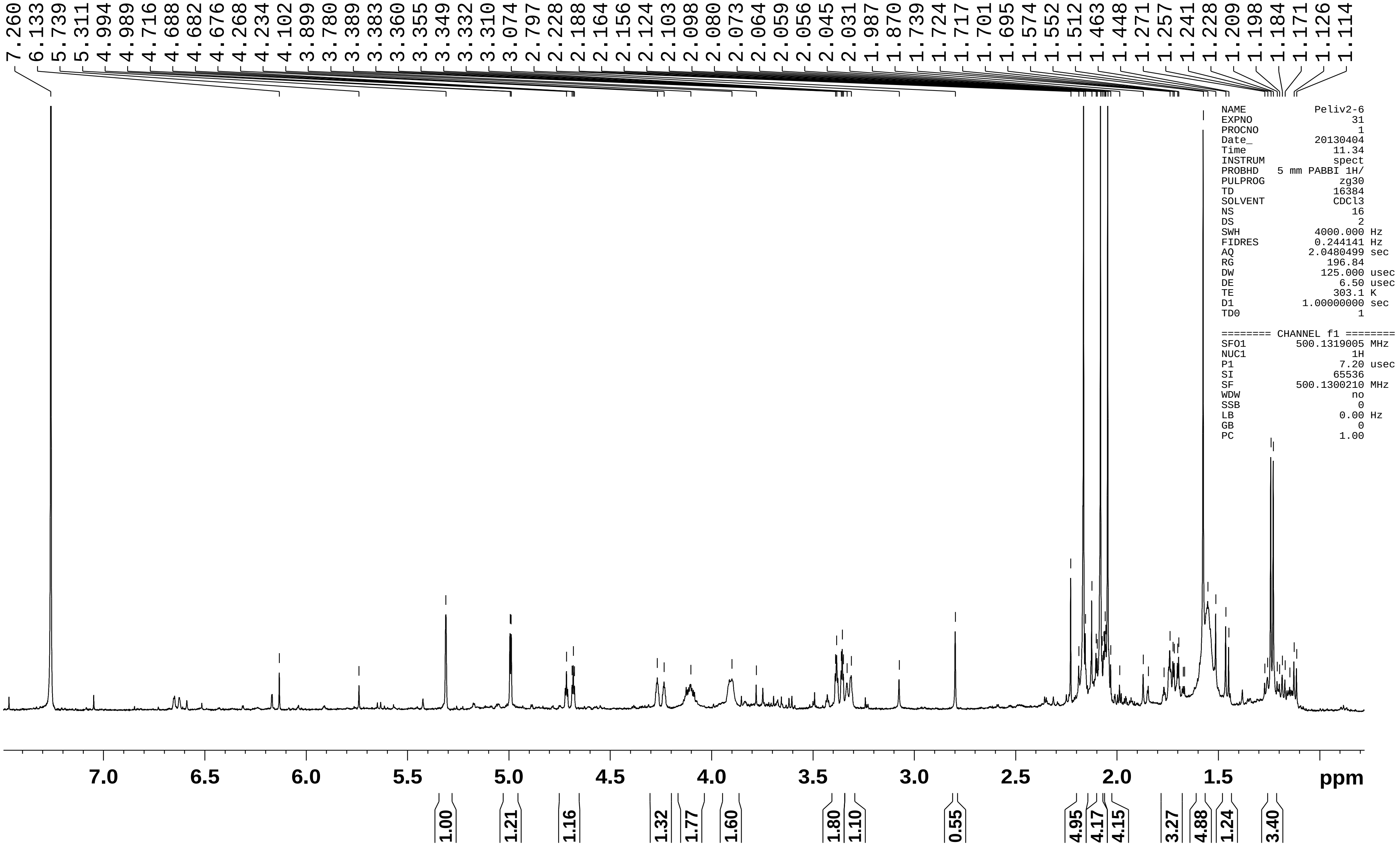

Figure S18. ^{1}H-^{1}H COSY (500 MHz, $CDCl_3$) spectrum of **3**.

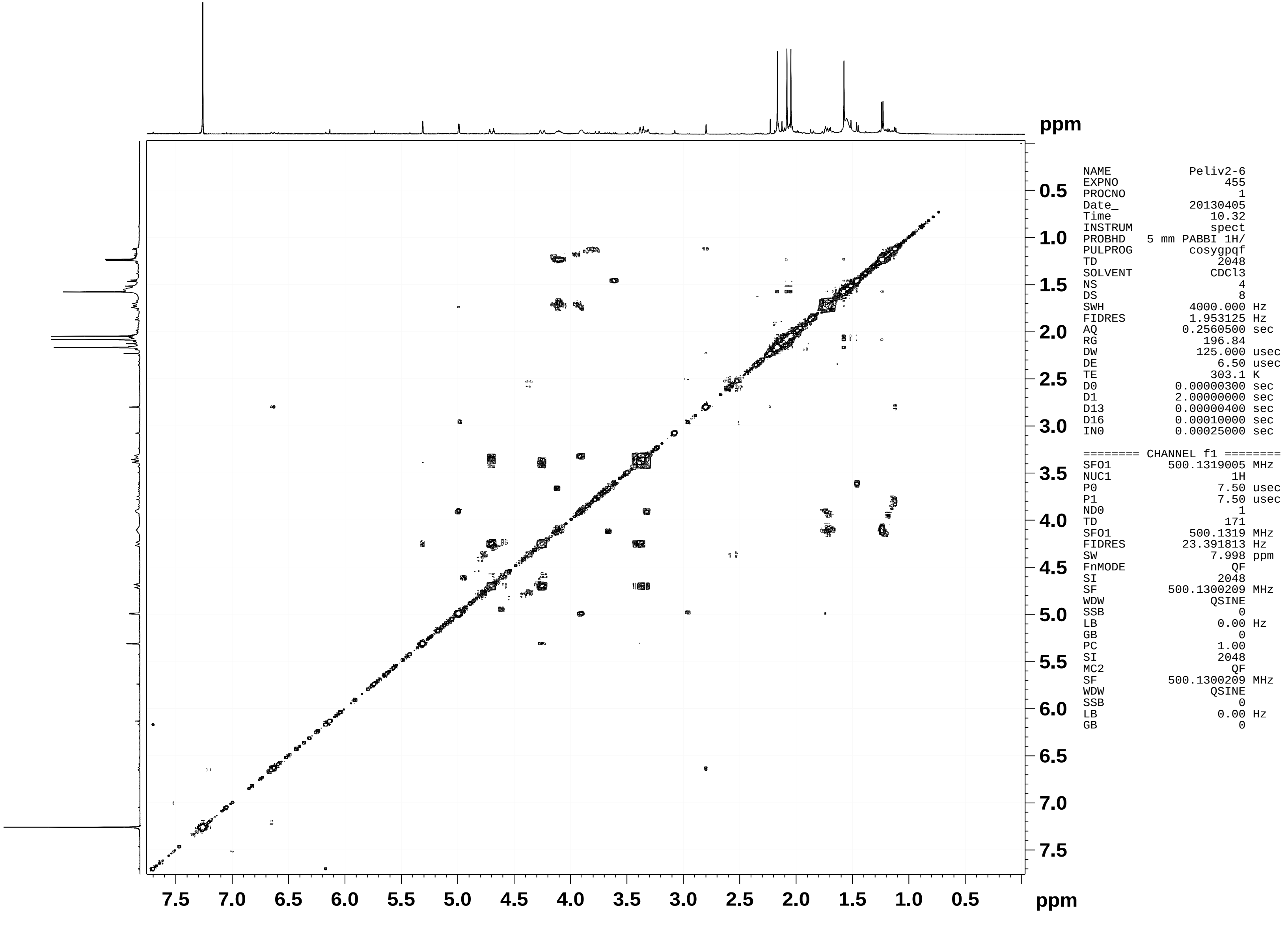

Figure S19. ^{13}C NMR (176 MHz, $CDCl_3$) spectrum of **3**.

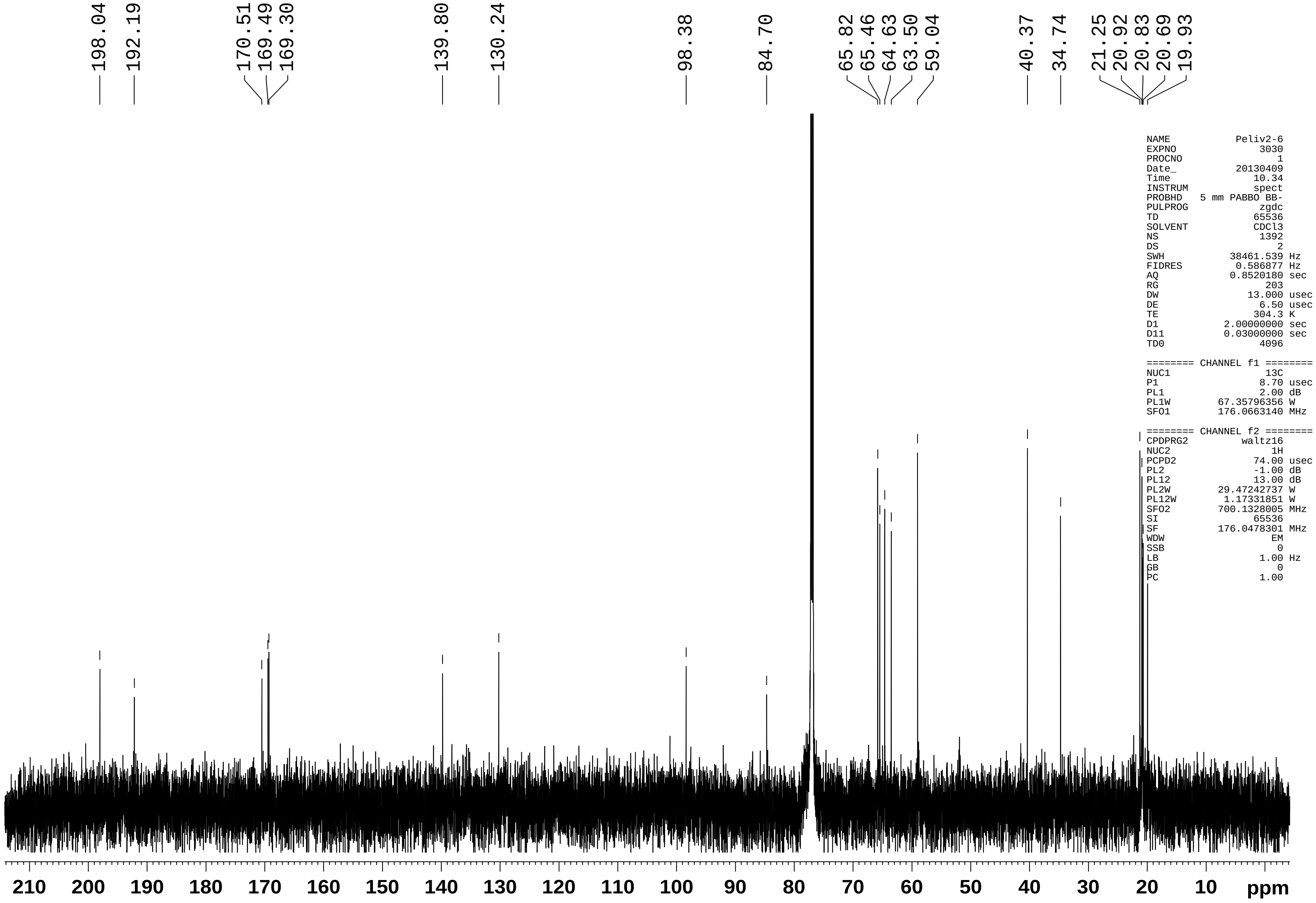

Figure S20. HSQC (500 MHz, $CDCl_3$) spectrum of **3**.

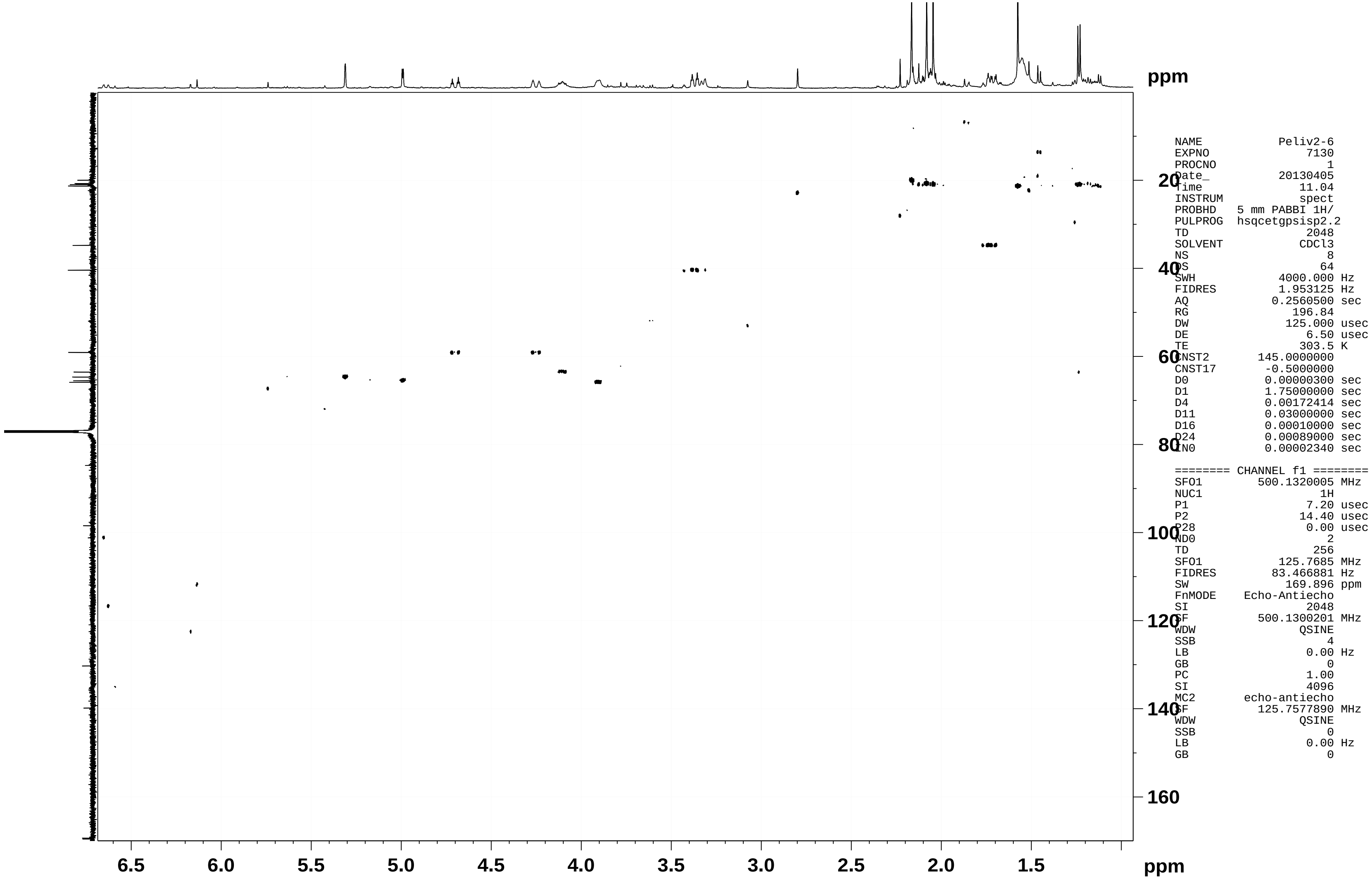

Figure S21. HMBC (500 MHz, $CDCl_3$) spectrum of **3**.

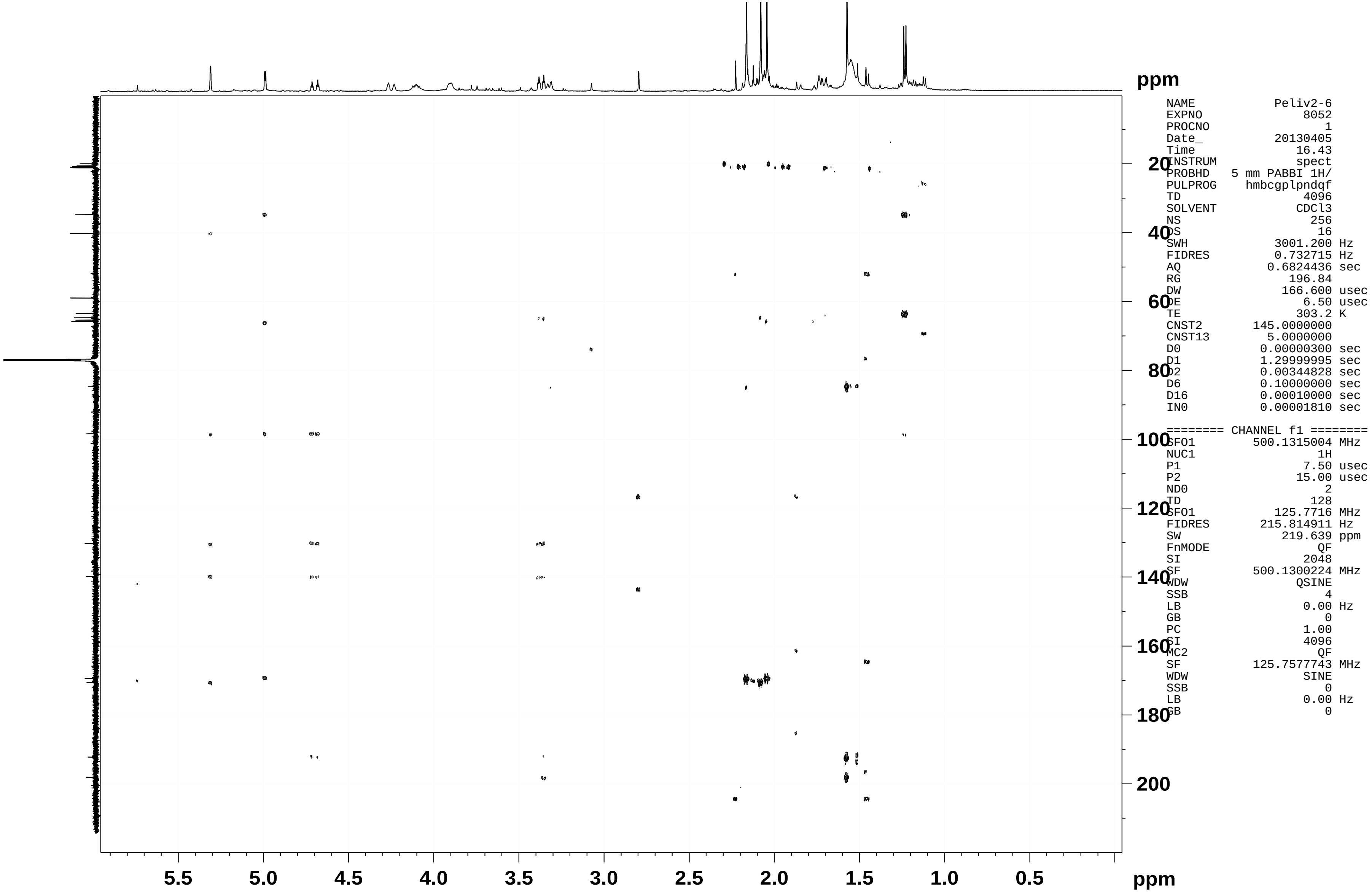

Figure S22. NOESY (700 MHz, $CDCl_3$) spectrum of **3**.

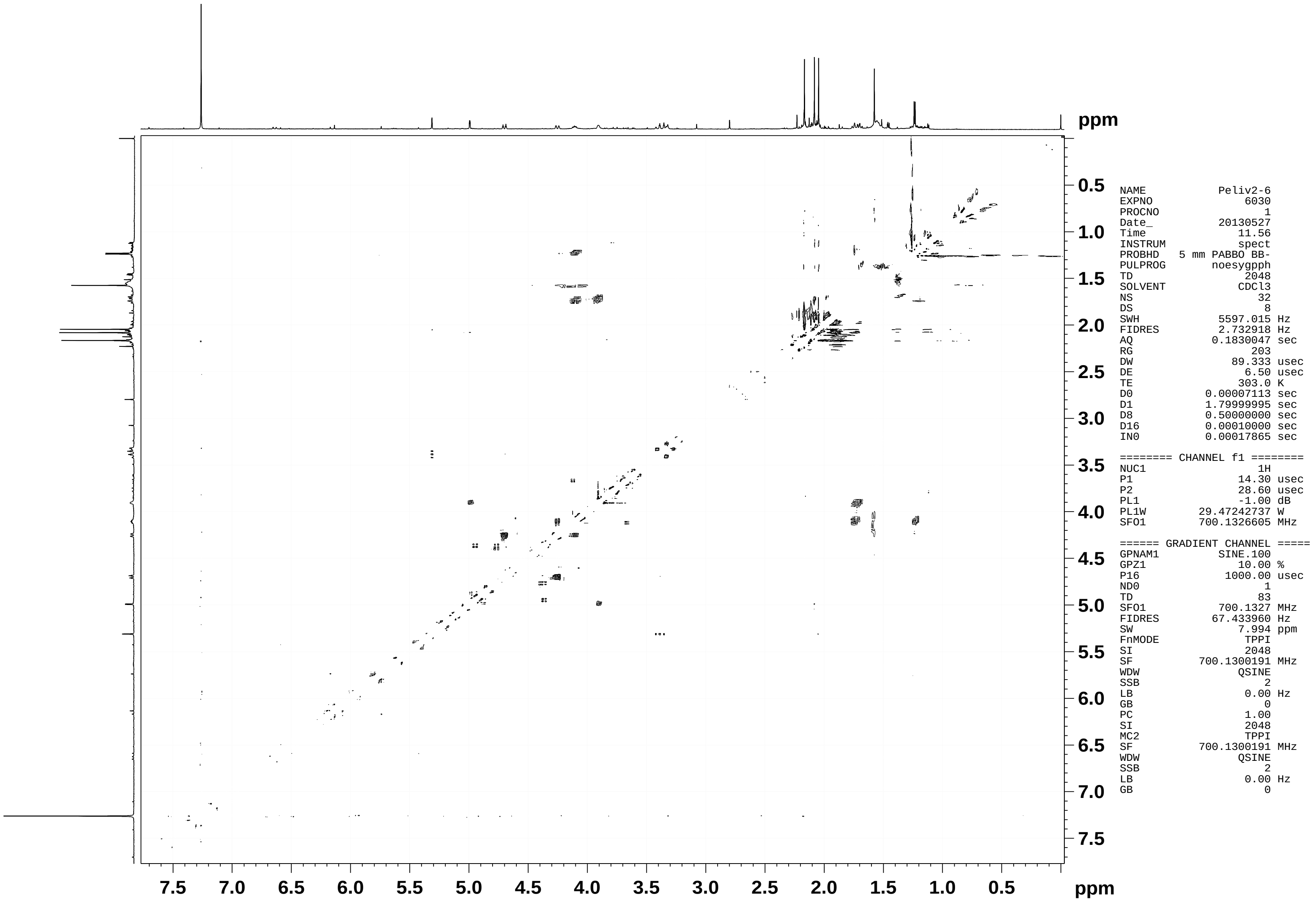

Figure S23. ^{1}H NMR (700 MHz, $CDCl_3$) spectrum of **4**.

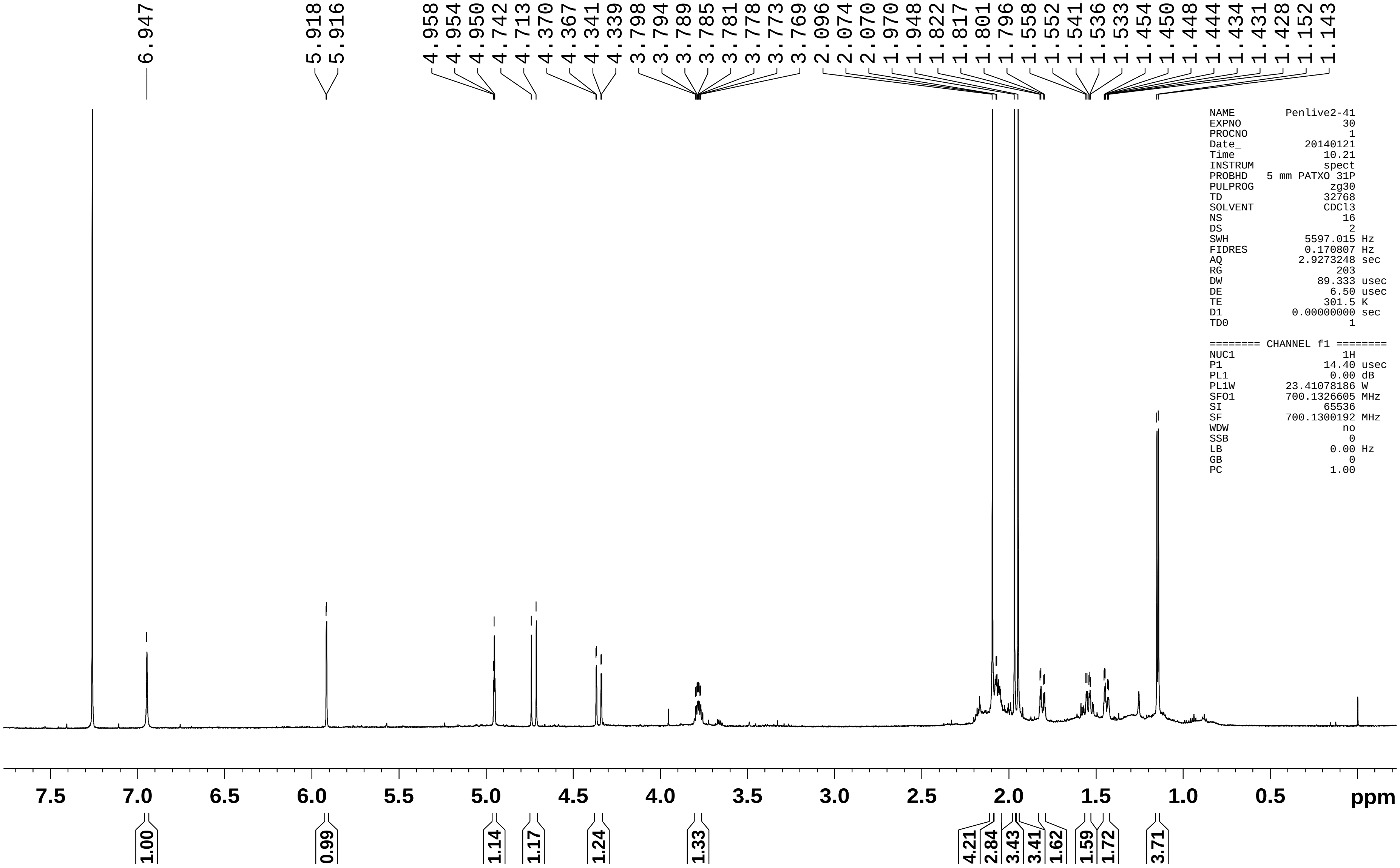

Figure S24. ^{1}H-^{1}H COSY (700 MHz, $CDCl_3$ spectrum of **4**.

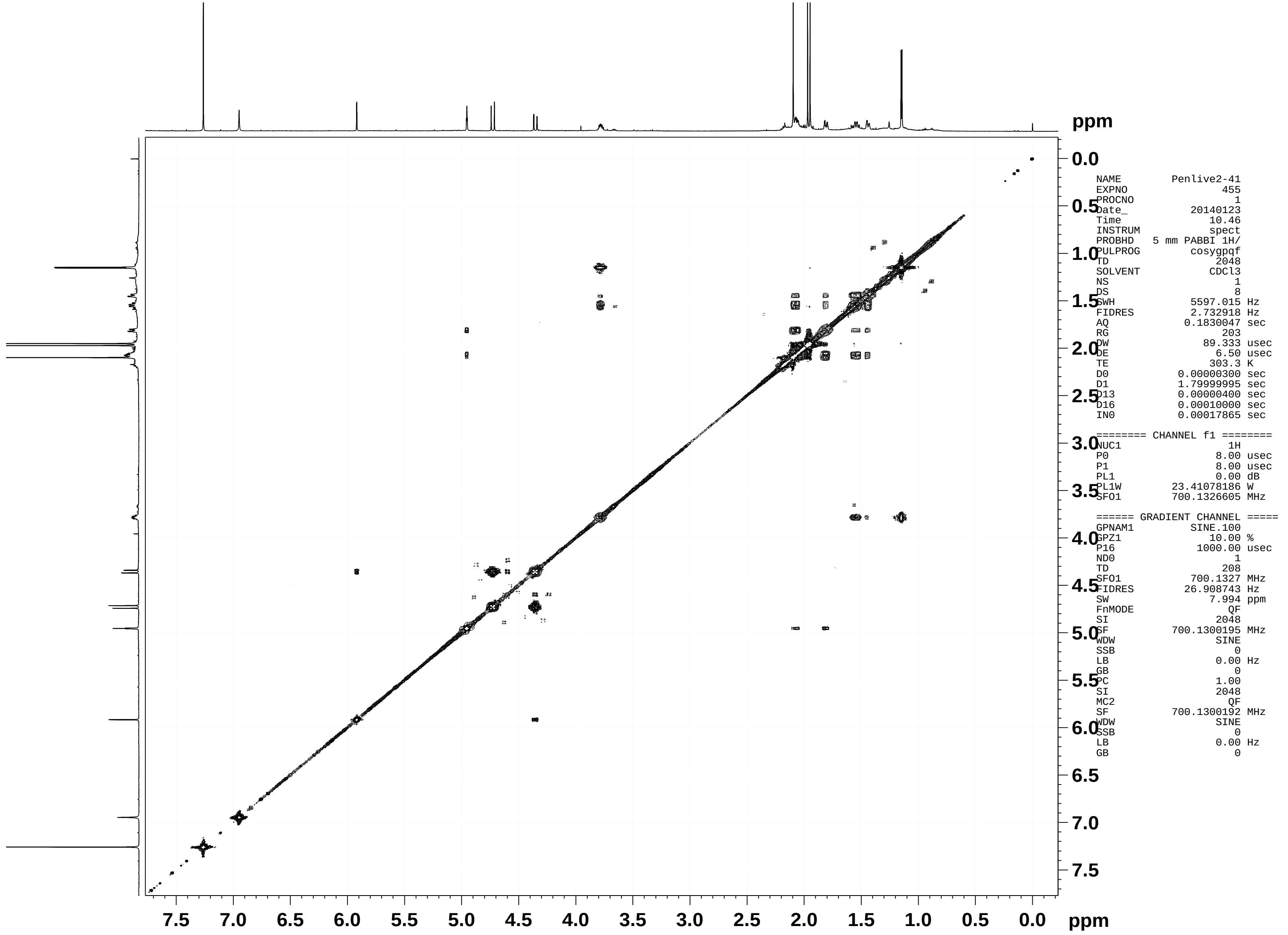

Figure S25. ^{13}C NMR (176 MHz, $CDCl_3$) spectra of **4**.

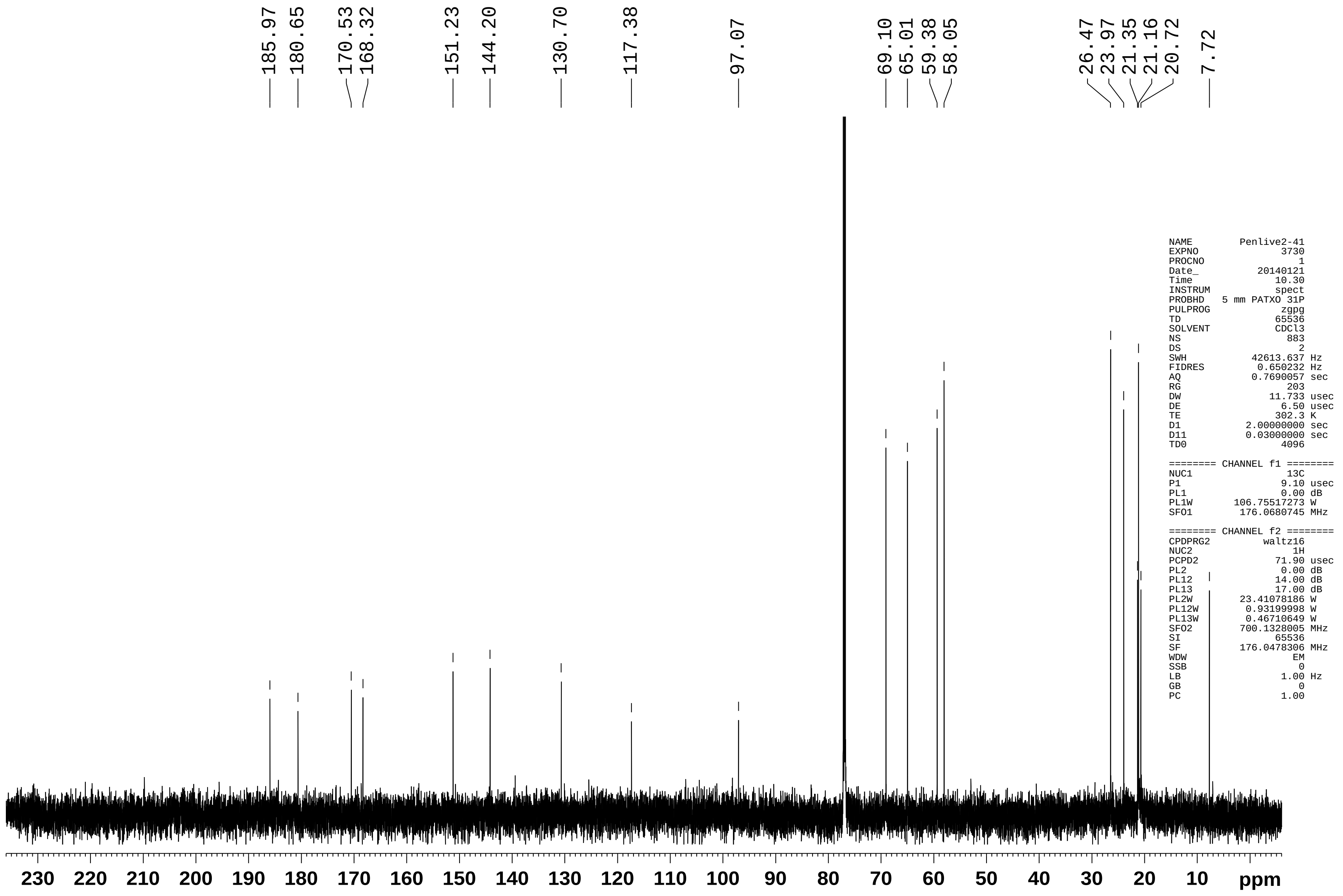

Figure S26. HSQC (700 MHz, $CDCl_3$) spectrum of **4**.

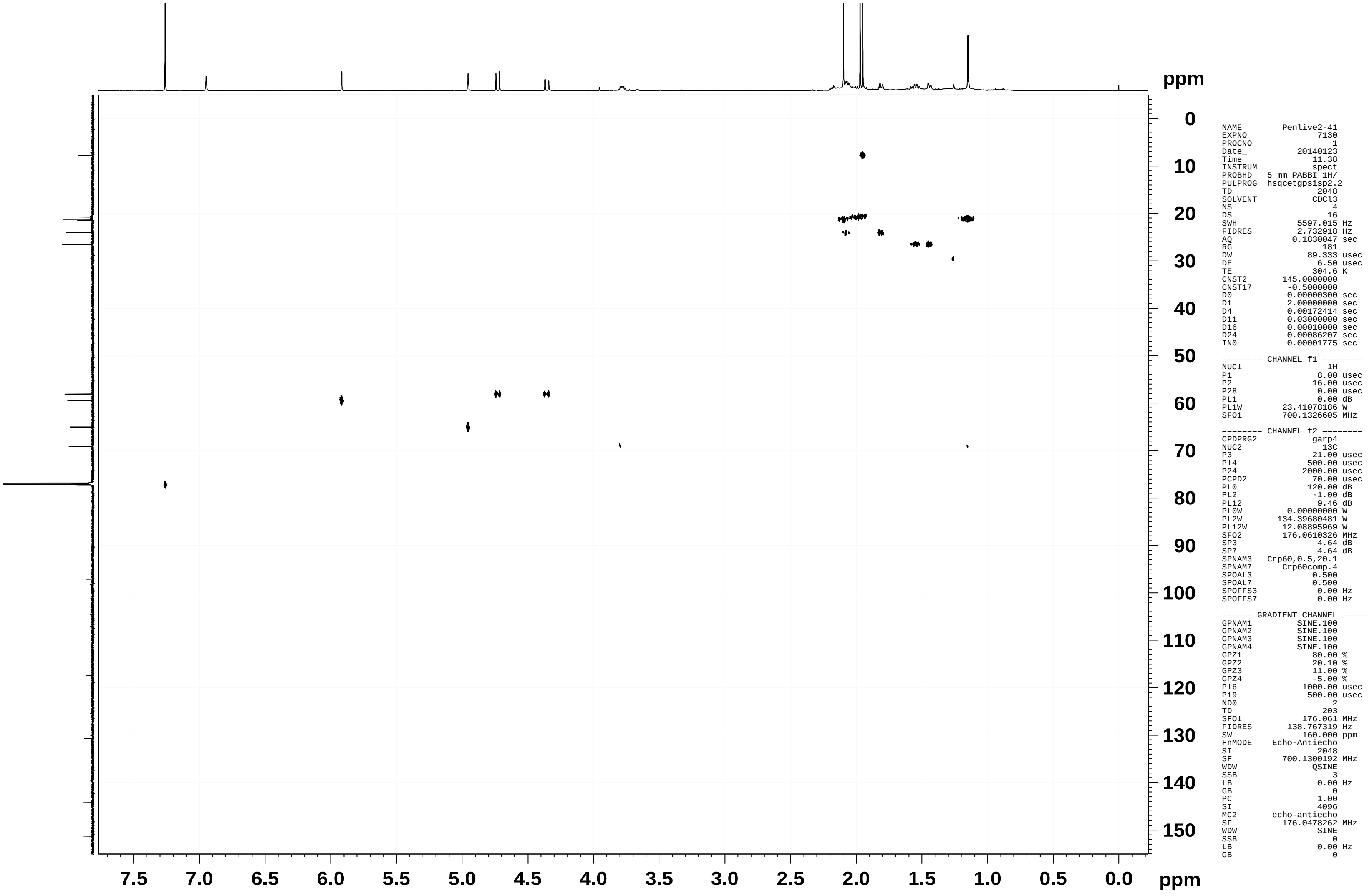

Figure S27. HMBC (700 MHz, $CDCl_3$) spectrum of **4**.

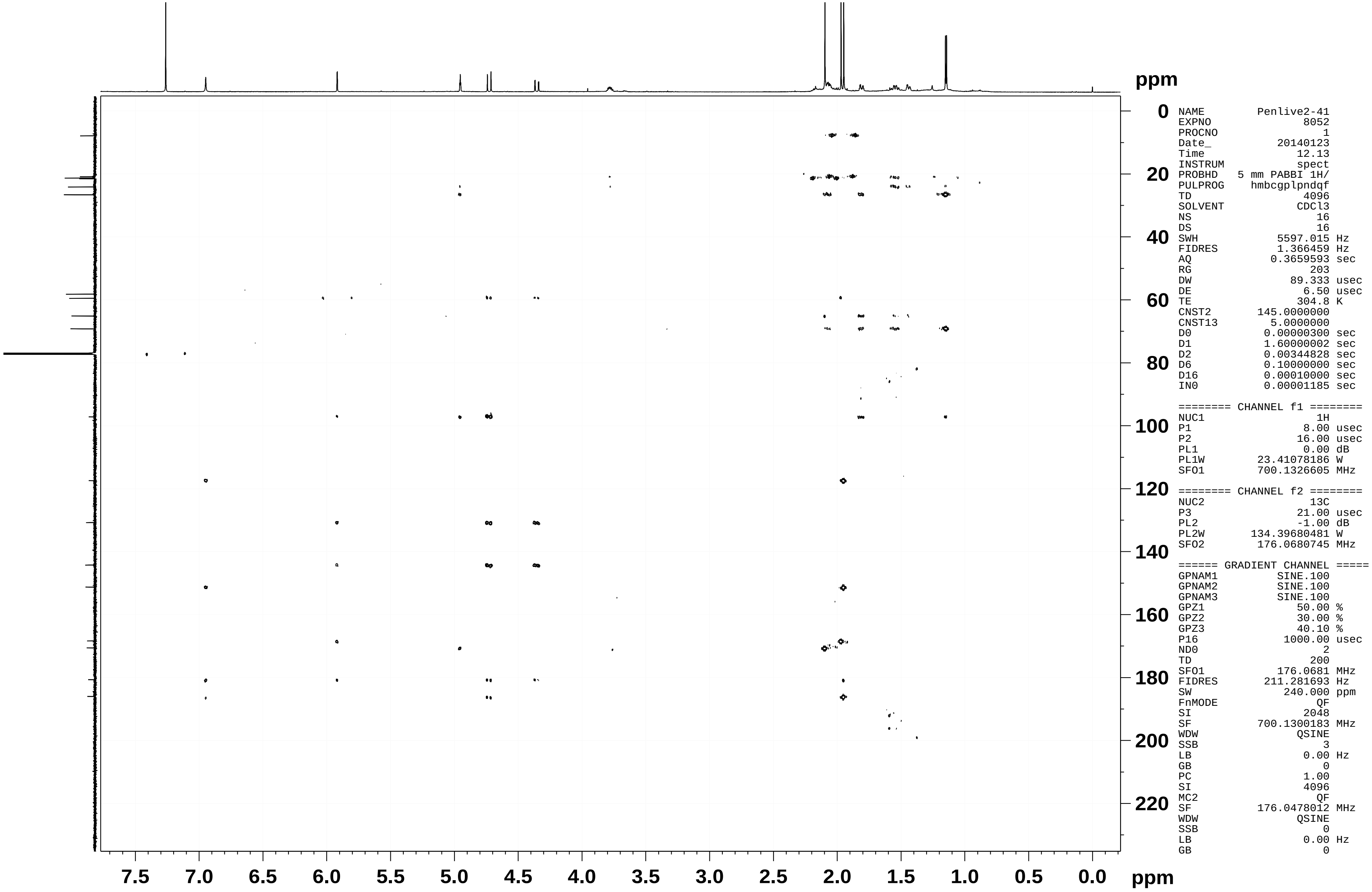

Figure S28. NOESY (700 MHz, $CDCl_3$) spectrum of **4**.

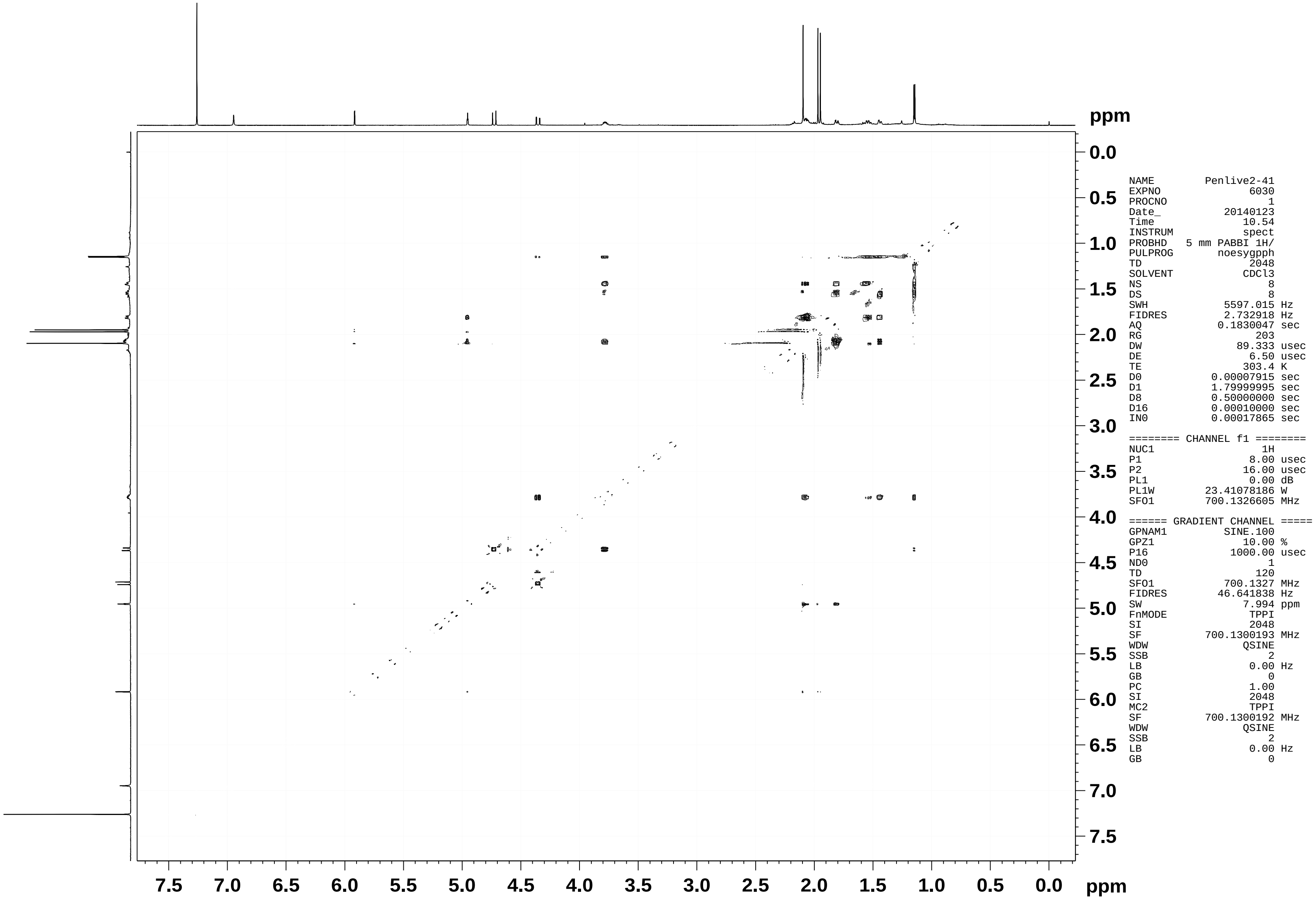

Figure S29. ^{1}H NMR (700 MHz, $CDCl_3$) spectrum of **5**.

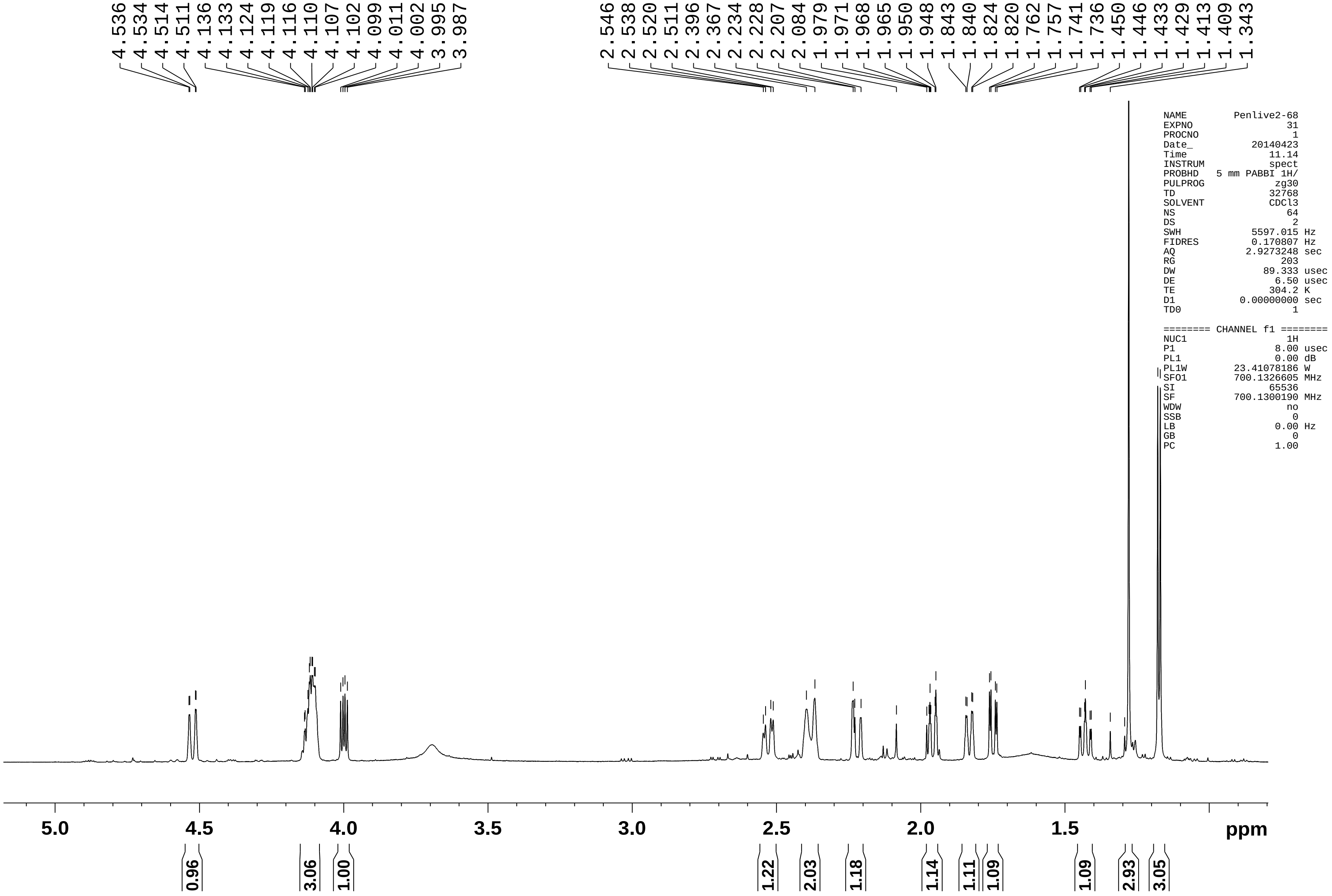

Figure S30. ^{1}H-^{1}H COSY (700 MHz, $CDCl_3$ spectrum of **5**.

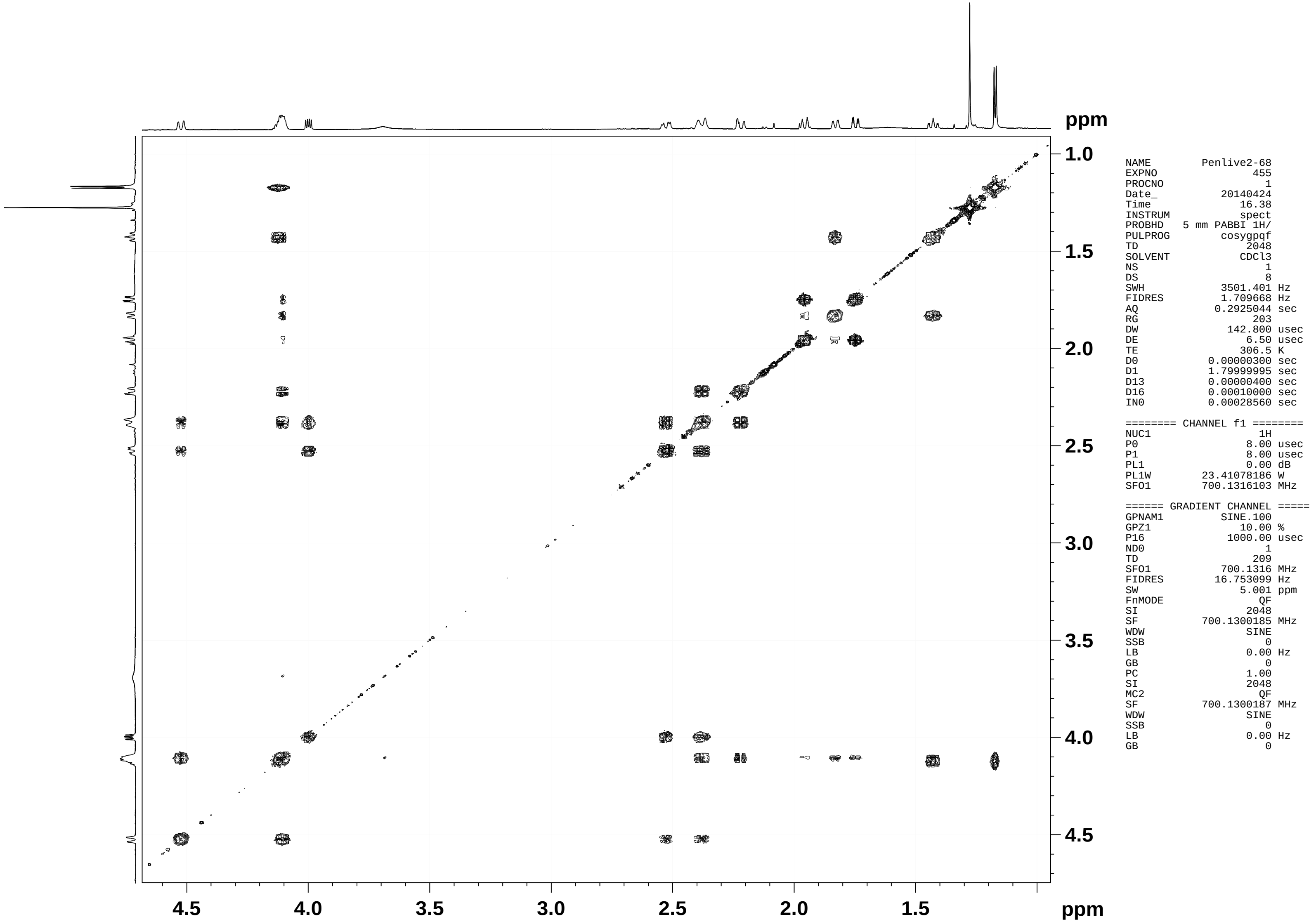

Figure S31. ^{13}C NMR (176 MHz, $CDCl_3$) spectra of **5**.

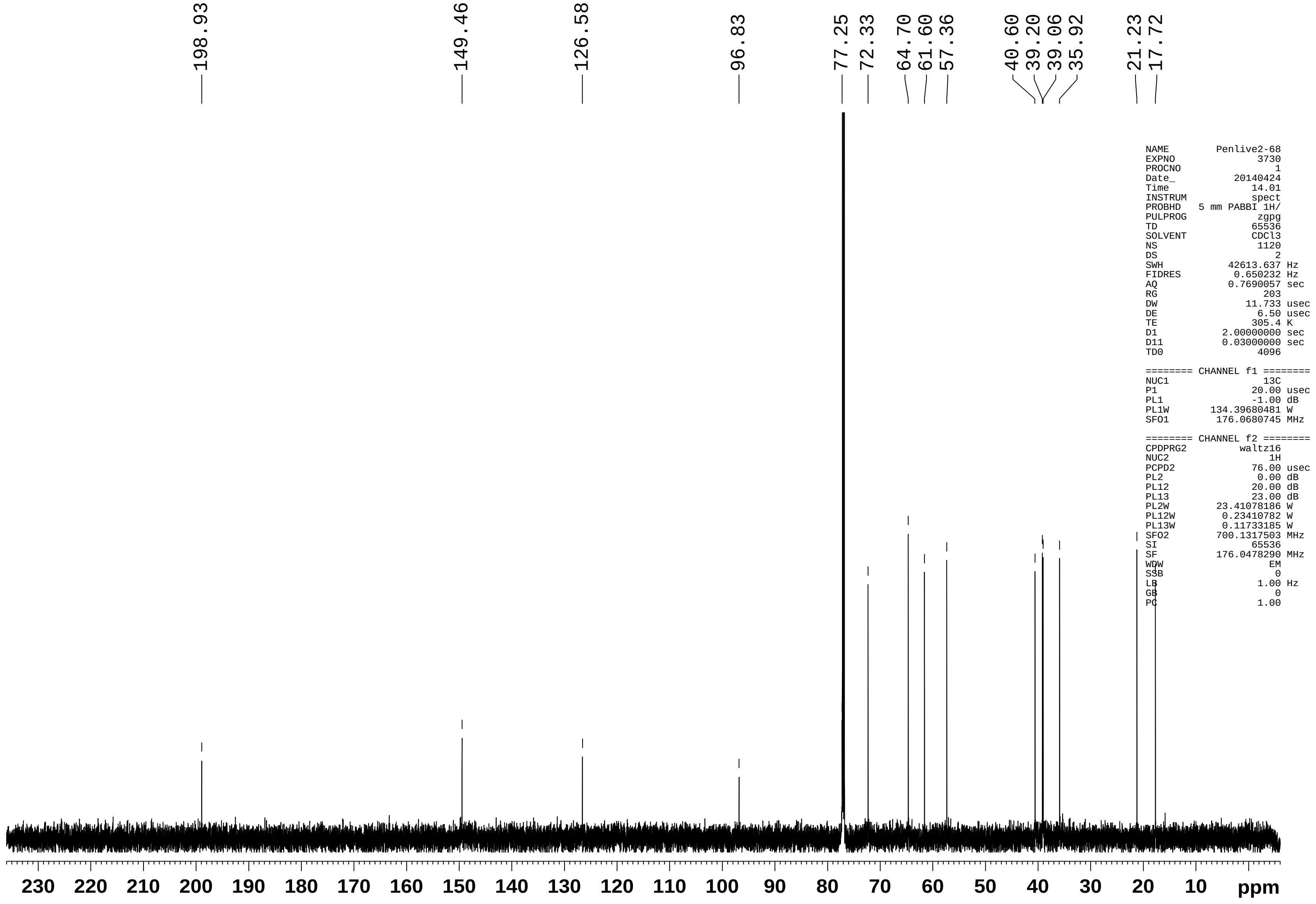

Figure S32. HSQC (700 MHz, $CDCl_3$) spectrum of **5**.

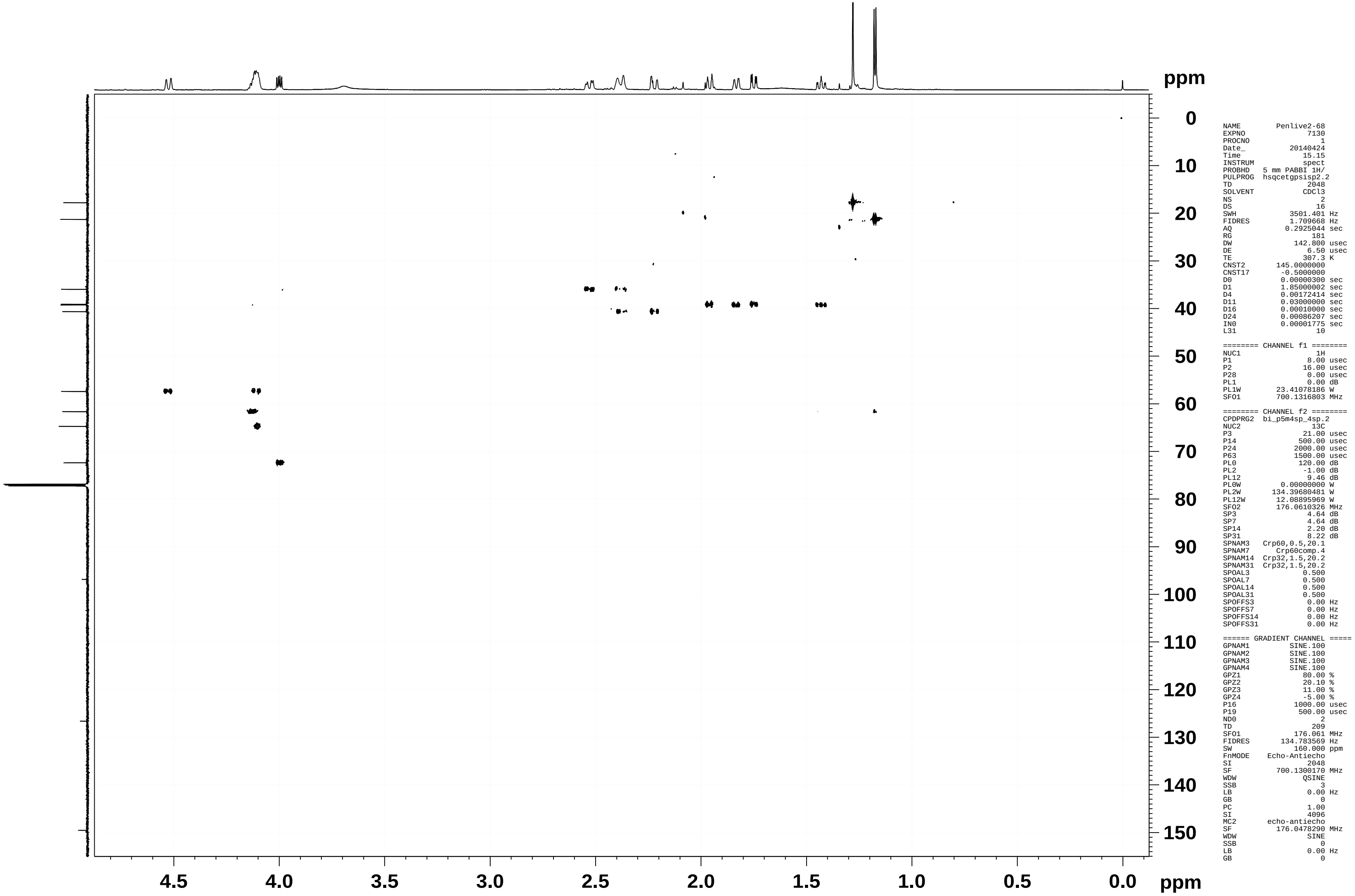

Figure S33. HMBC (700 MHz, $CDCl_3$) spectrum of **5**.

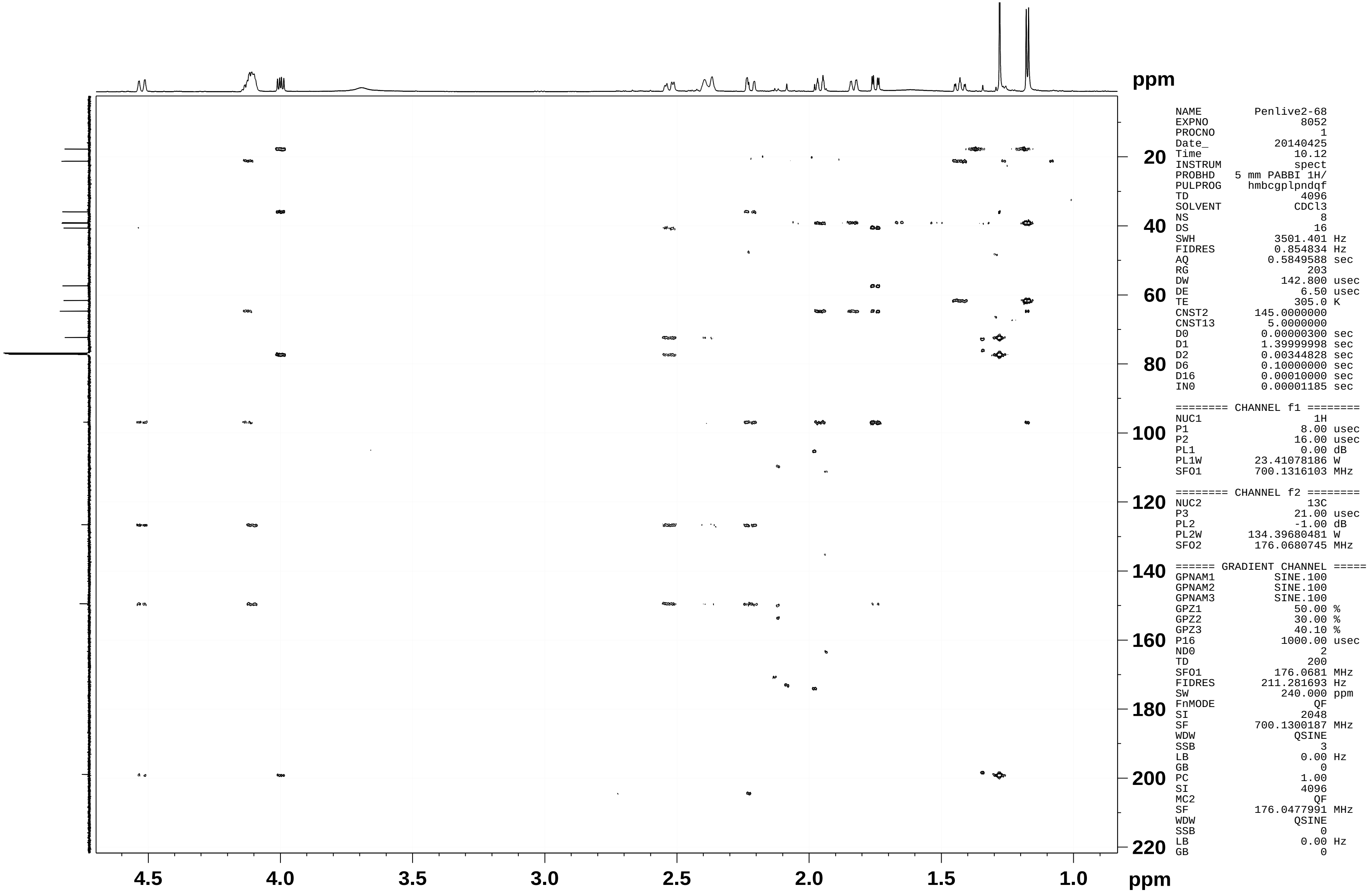

Figure S34. NOESY (700 MHz, $CDCl_3$) spectrum of **5**.

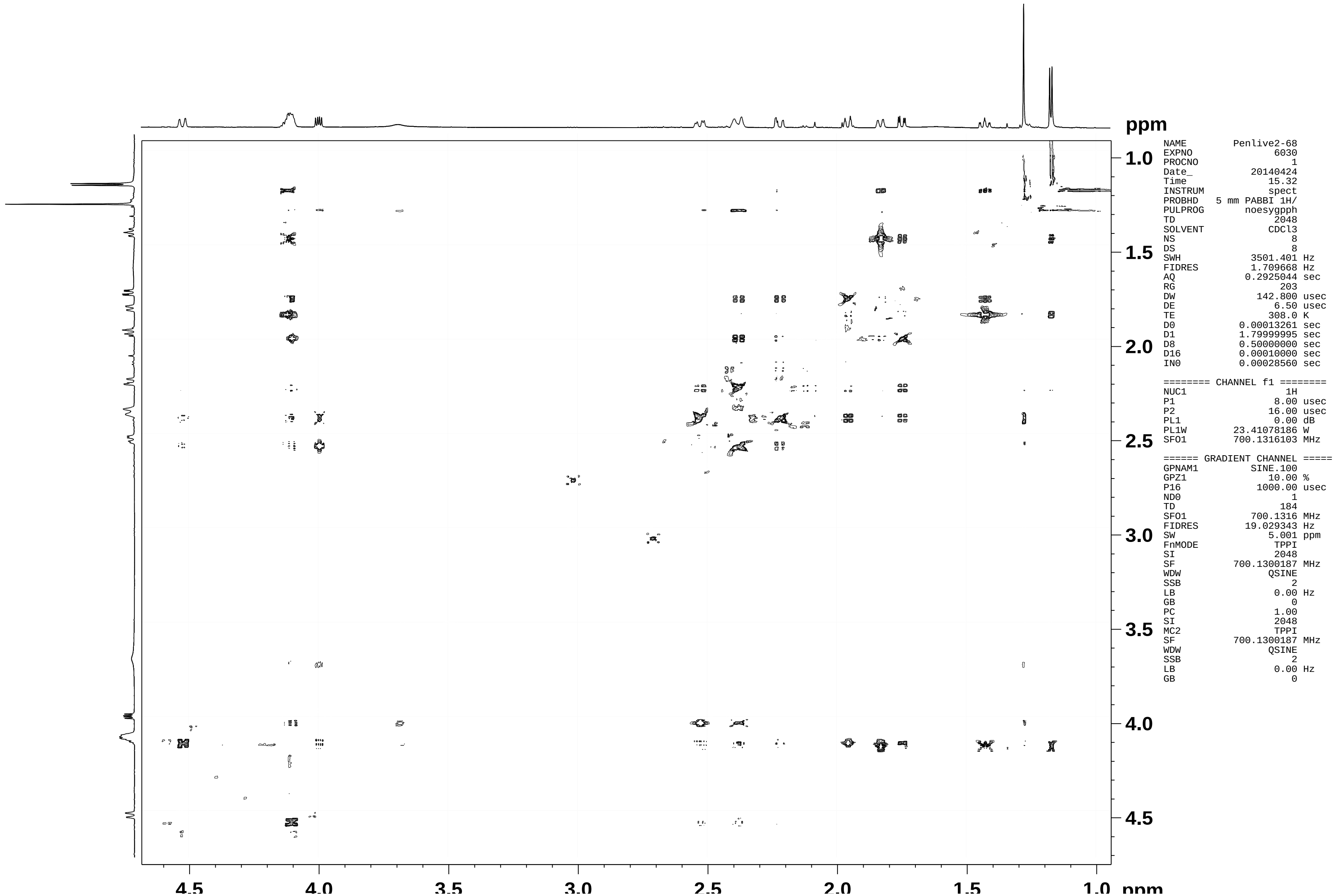

Figure S35. ^{1}H NMR (500 MHz, $CDCl_3$) spectrum of (*S*)-MTPA ester of **5a**.

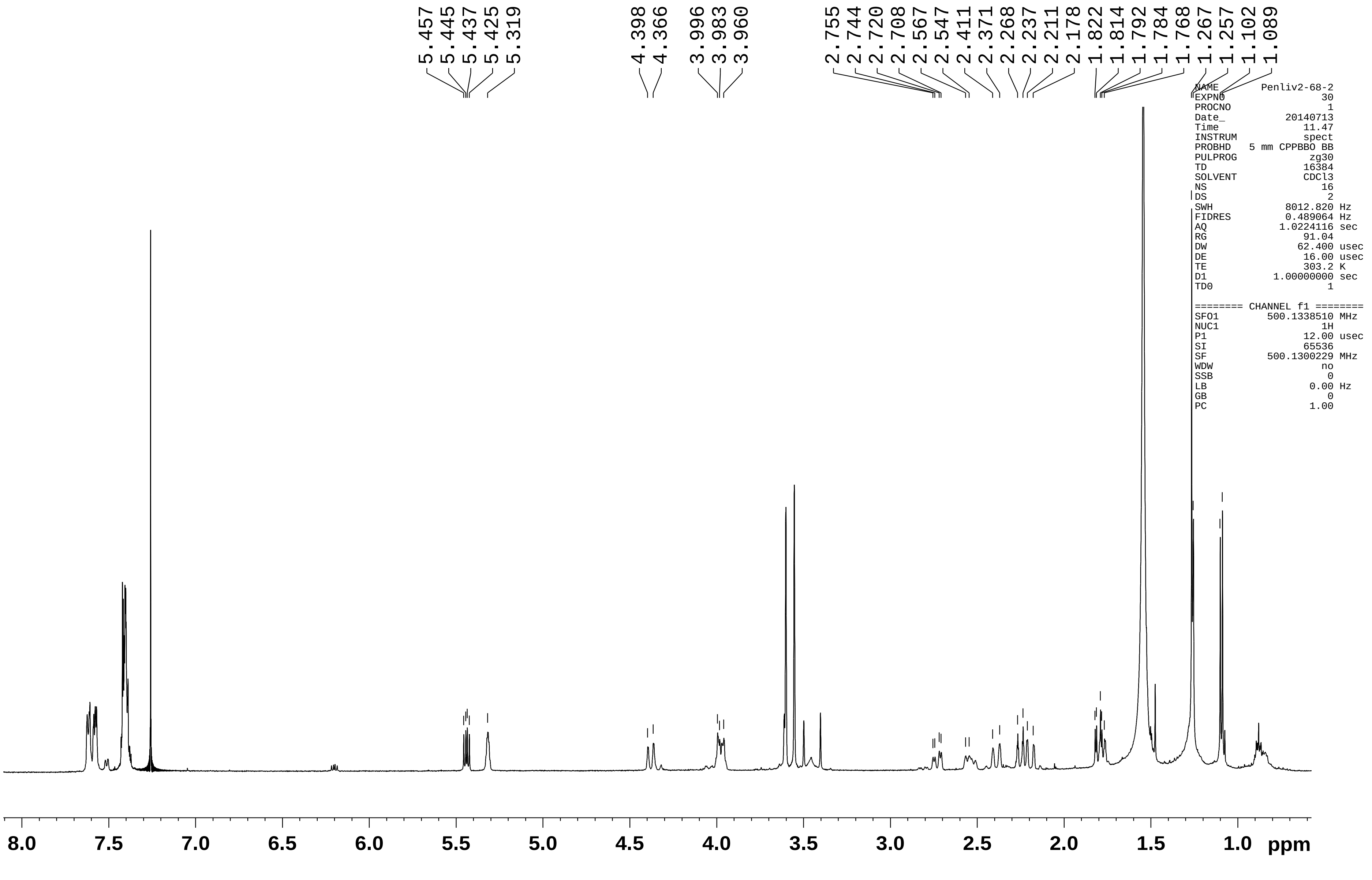

Figure S36. ^{1}H-^{1}H COSY (500 MHz, $CDCl_3$) spectrum of (*S*)-MTPA ester of **5a**.

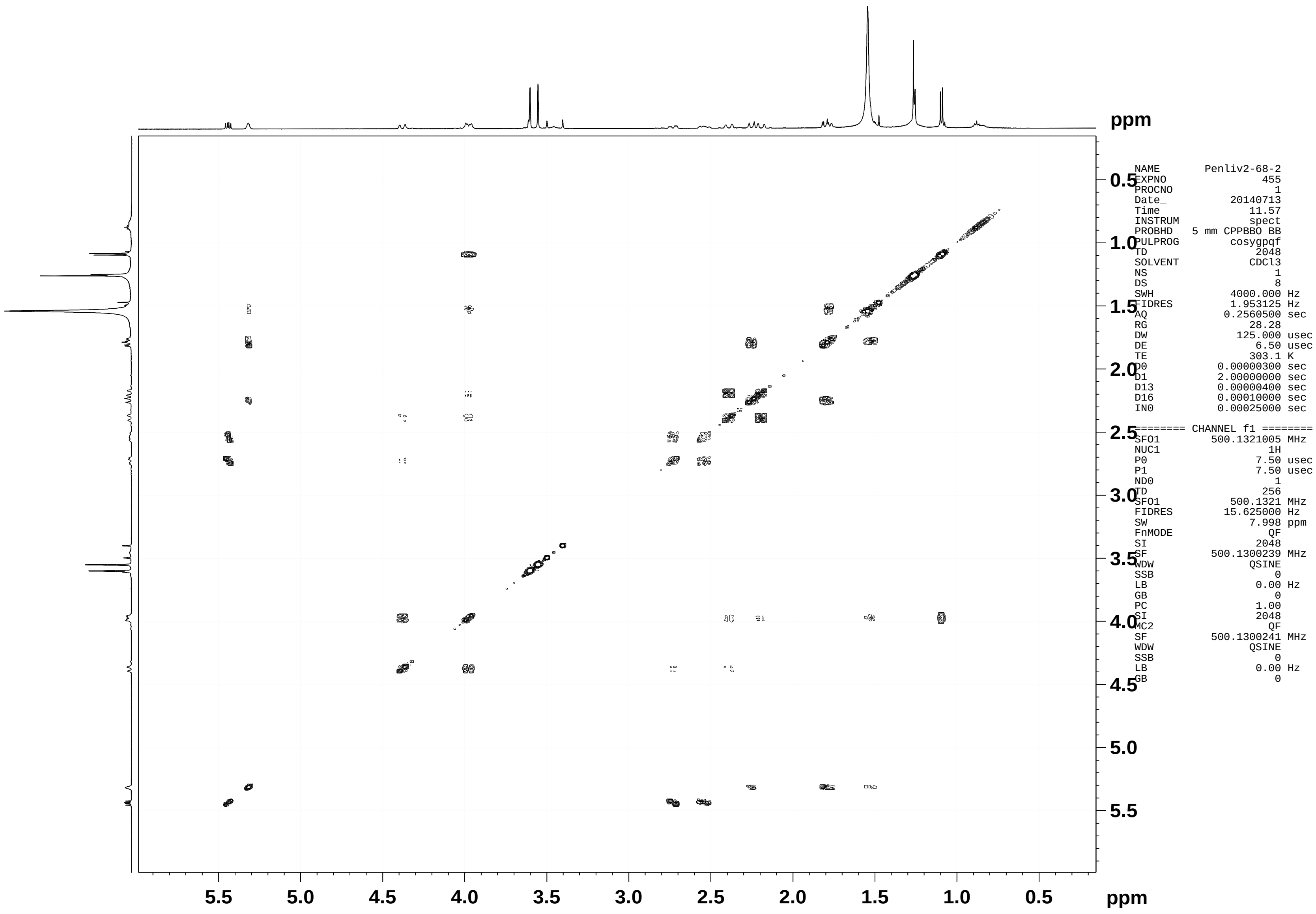

Figure S37. ^{1}H NMR (500 MHz, $CDCl_3$) spectrum of (*R*)-MTPA ester of **5b**.

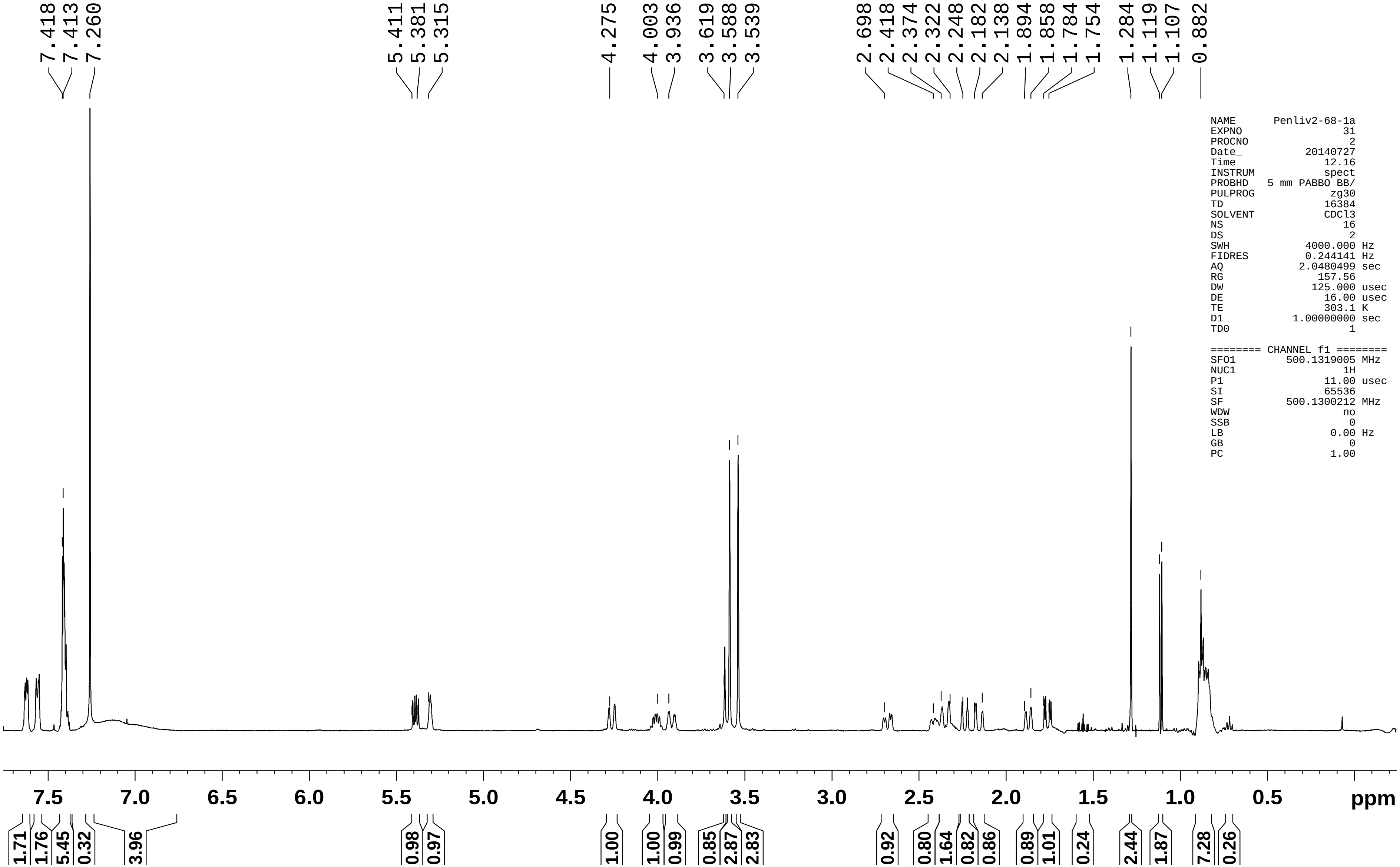

Figure S38. ^{1}H-^{1}H COSY (500 MHz, $CDCl_3$) spectrum of (*R*)-MTPA ester of **5b**.

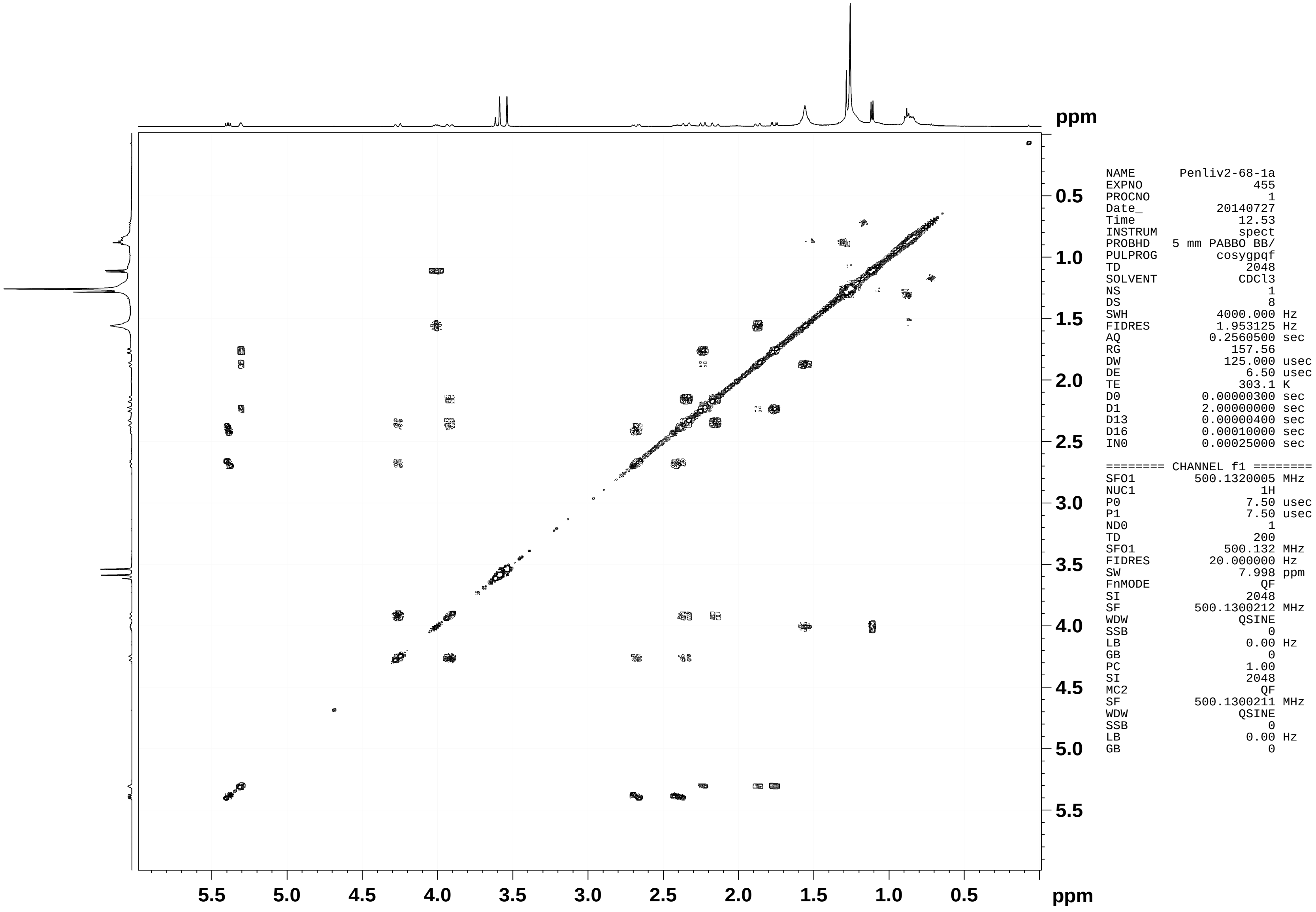

Figure S39. ^{1}H NMR (700 MHz, DMSO) spectrum of **5**.

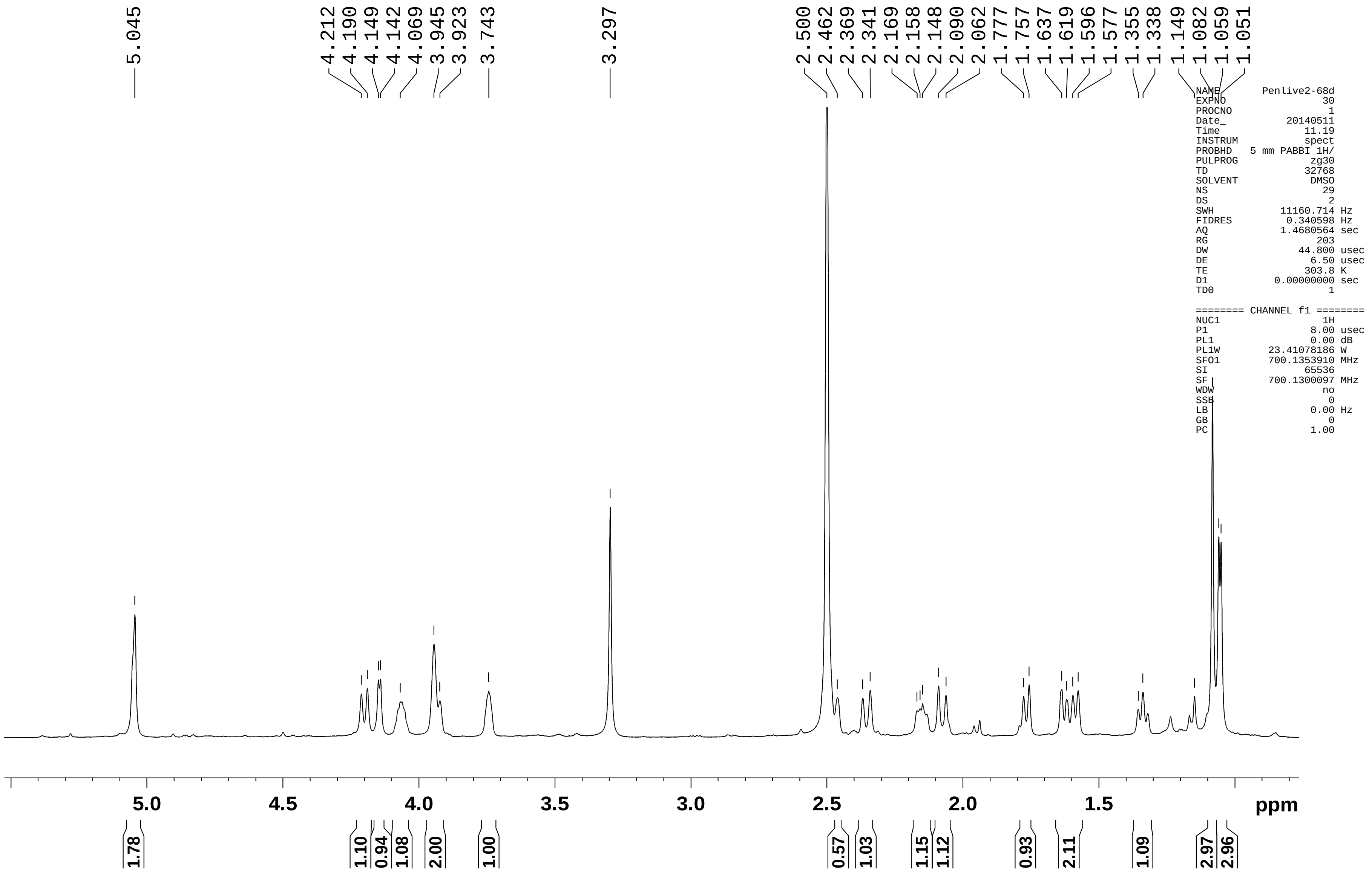

Figure S40. ^{1}H-^{1}H COSY (700 MHz, DMSO) spectrum of **5**.

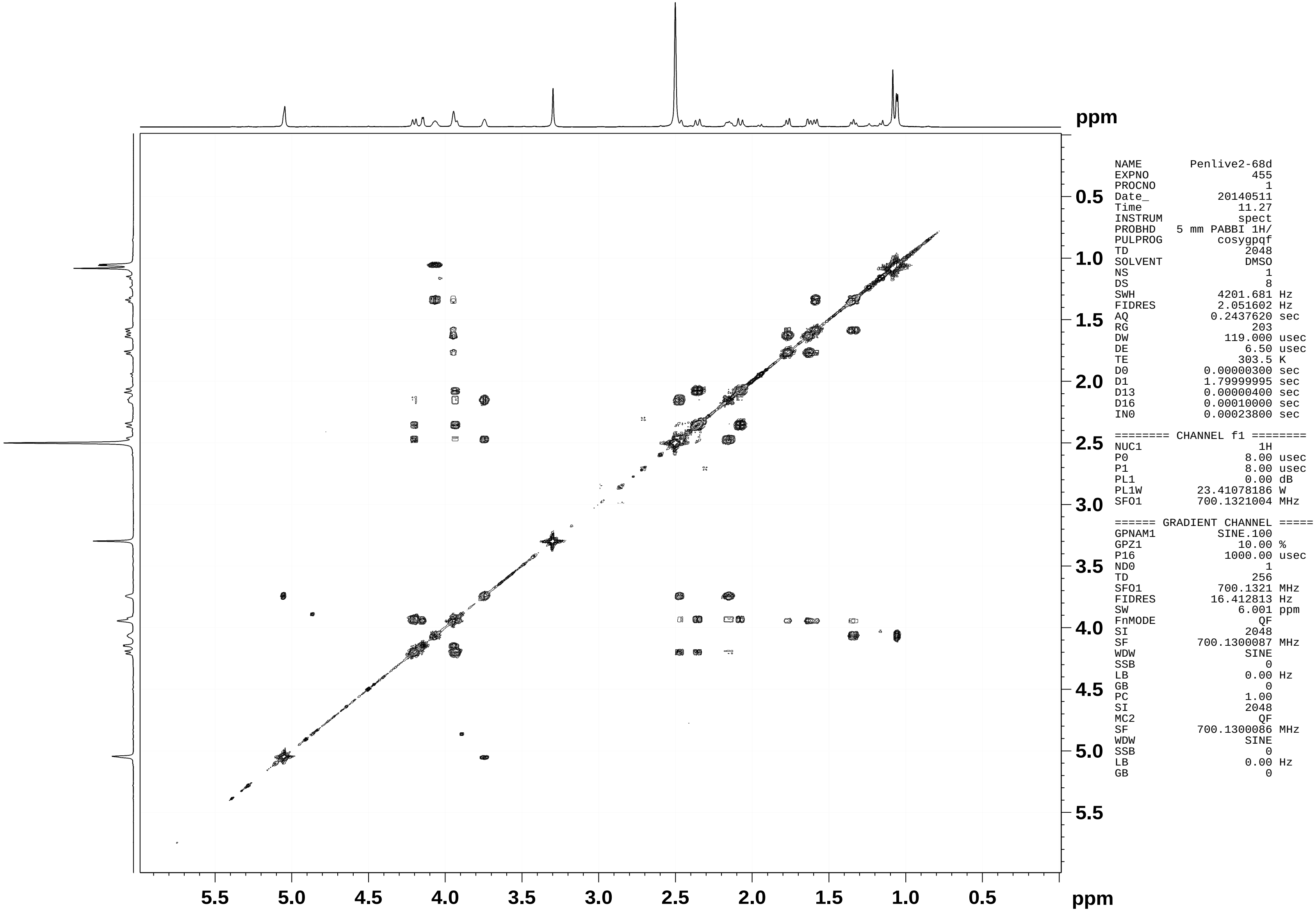

Figure S41. ^{13}C NMR (176 MHz, DMSO) spectrum of **5**.

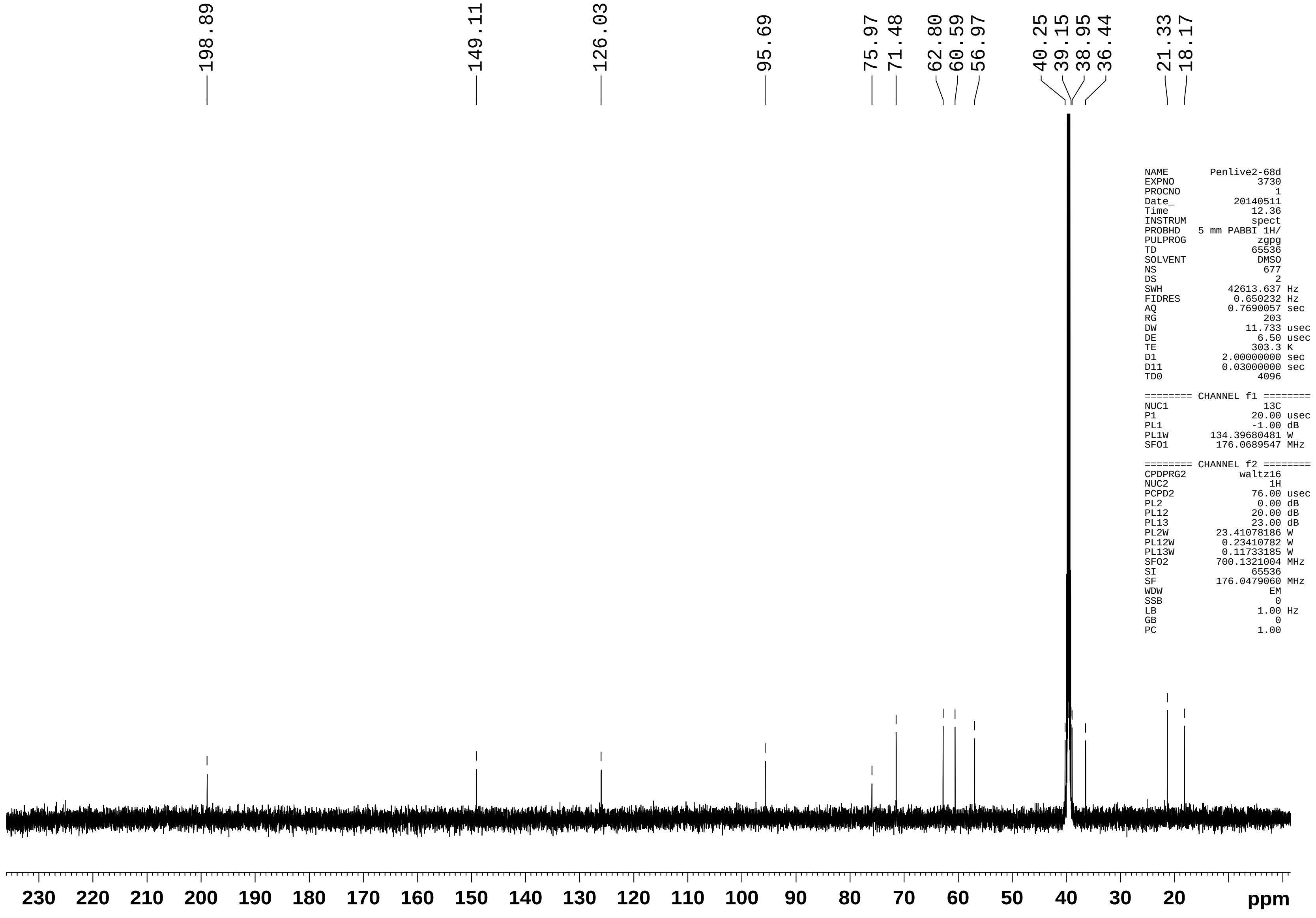

Figure S42. HSQC (700 MHz, DMSO) spectrum of **5**.

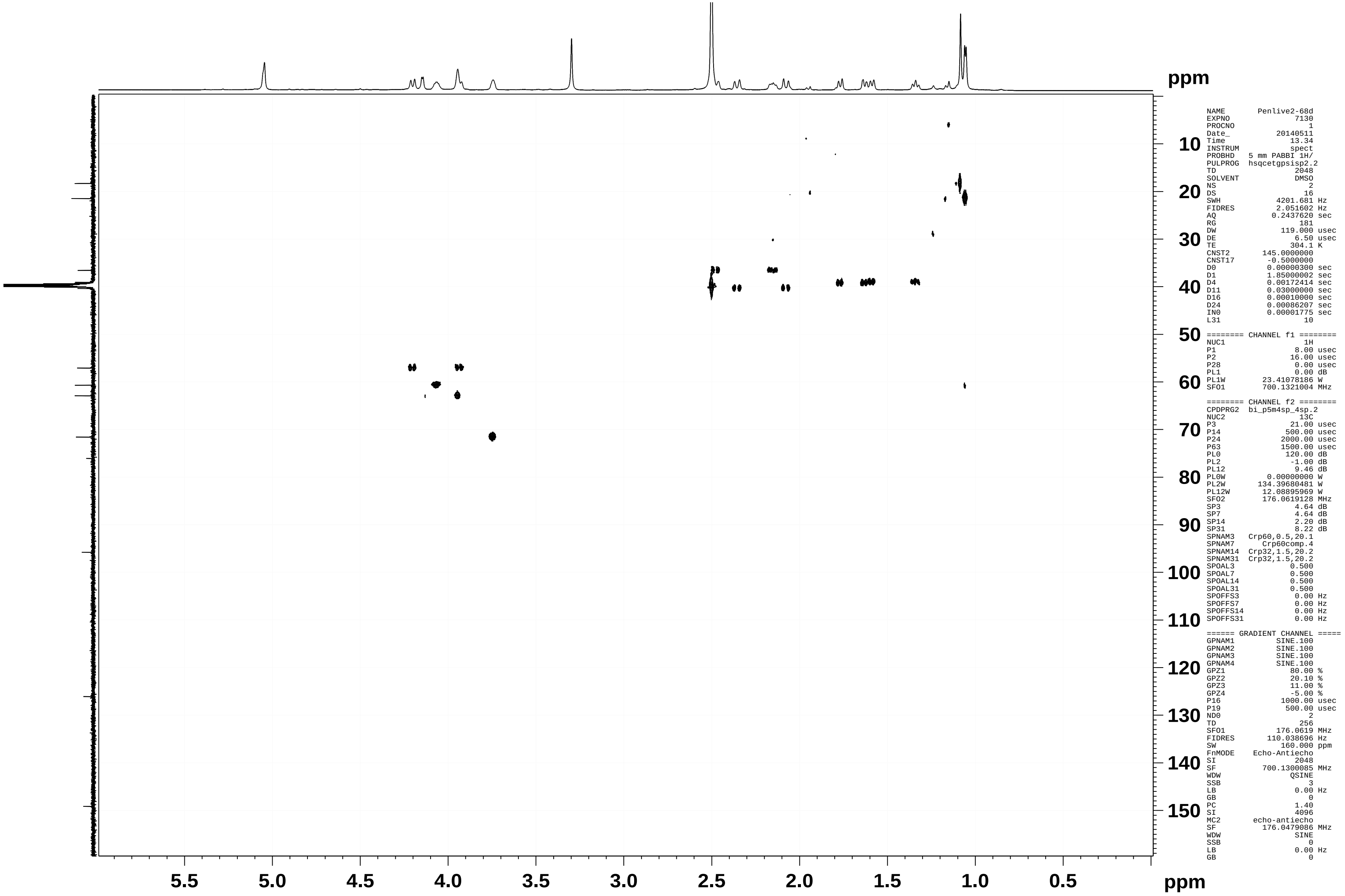

Figure S43. HMBC (700 MHz, DMSO) spectrum of **5**.

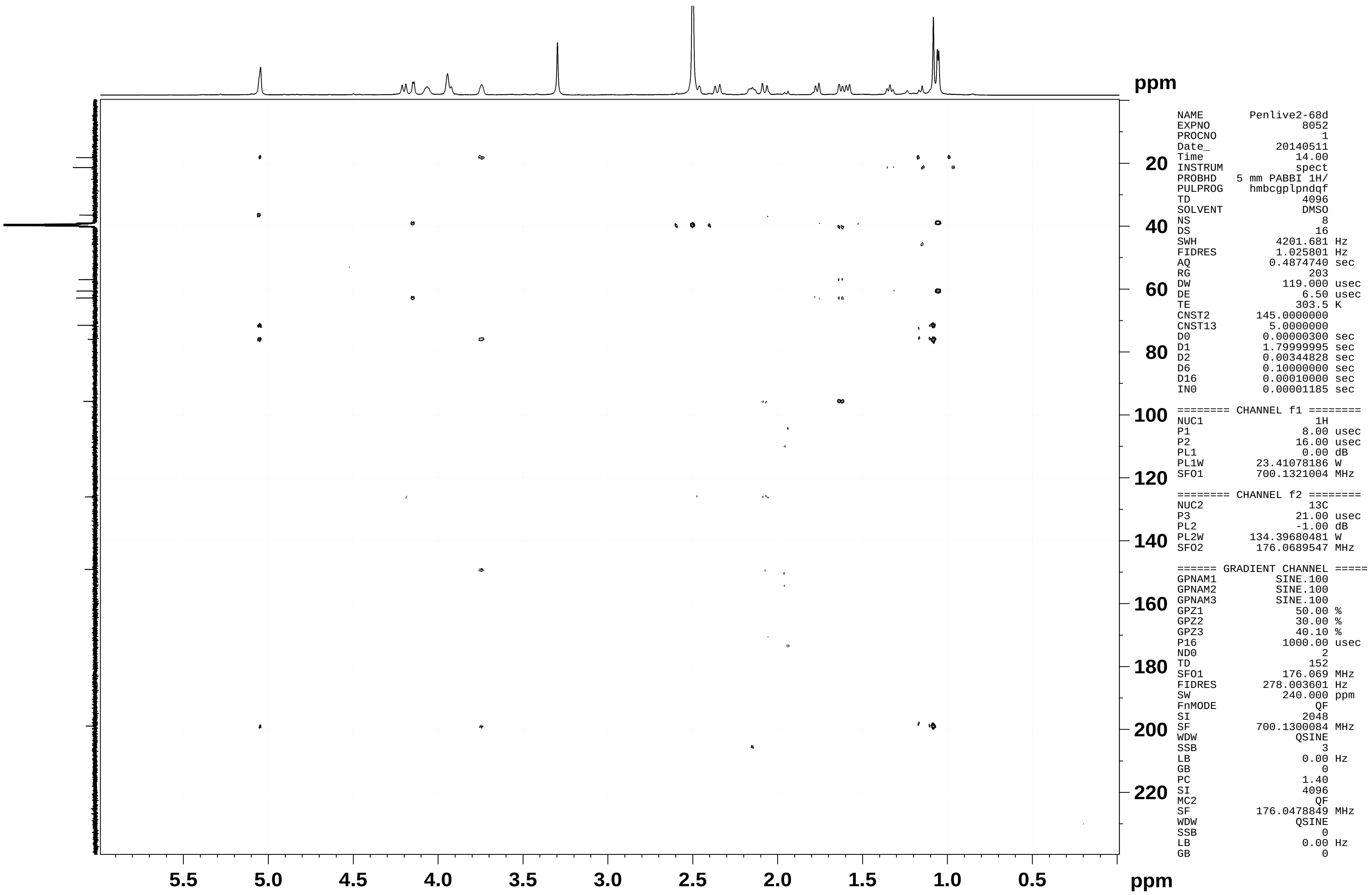

Figure S44. NOESY (700 MHz, DMSO) spectrum of **5**.

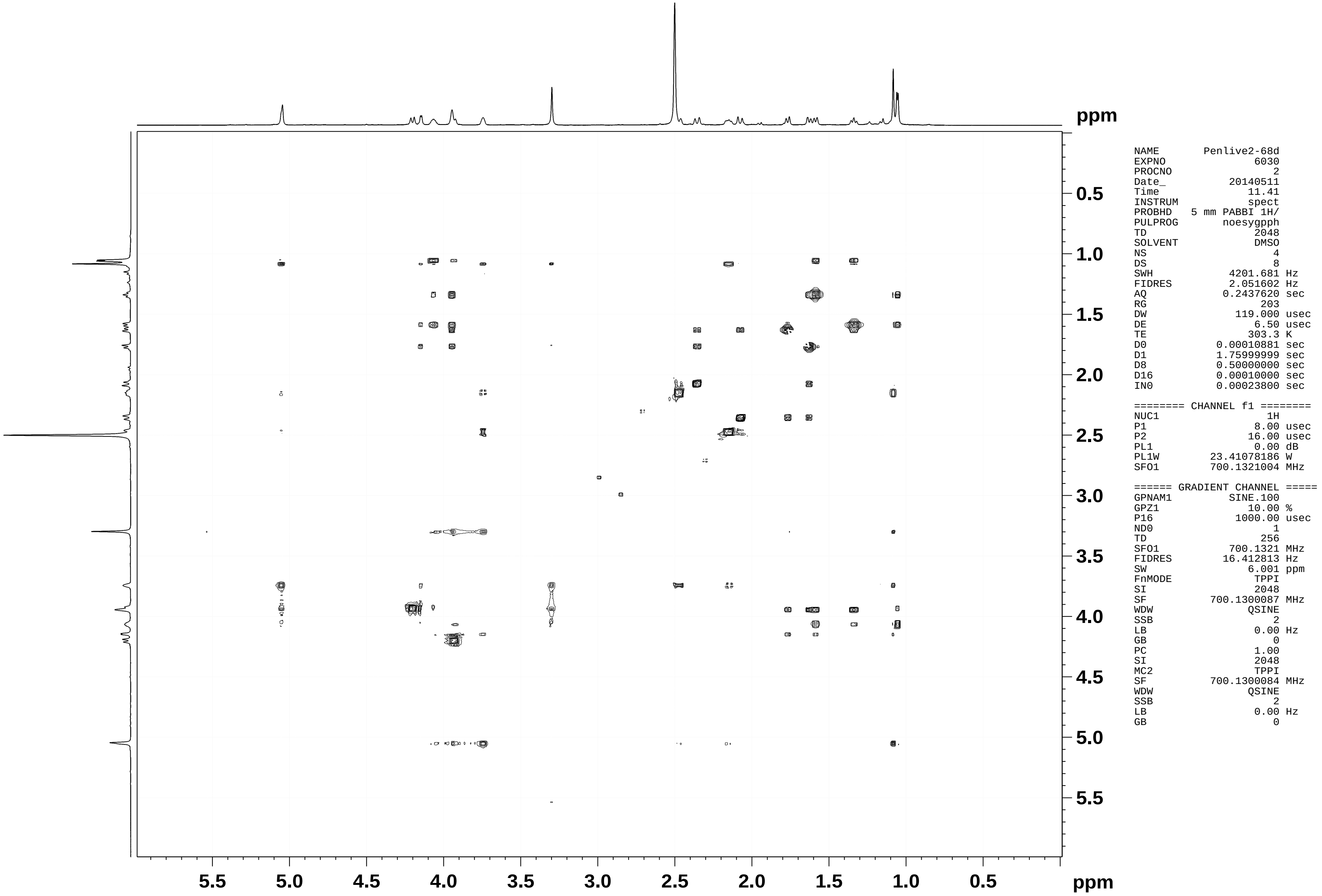

Figure S45. ^{1}H NMR (500 MHz, $CDCl_3$) spectrum of **6**.

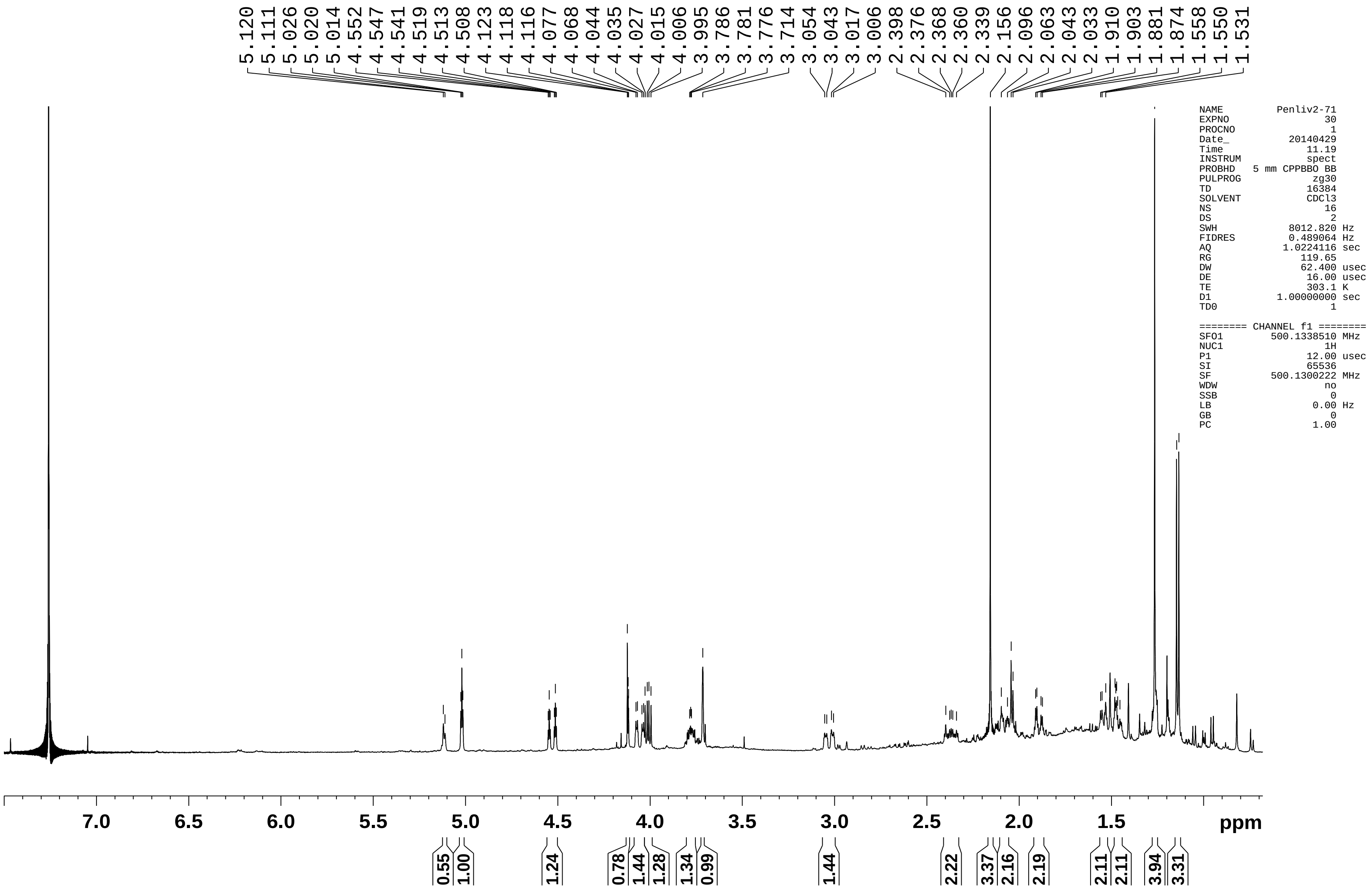

Figure S46. ^{1}H-^{1}H COSY (500 MHz, $CDCl_3$) spectrum of **6**.

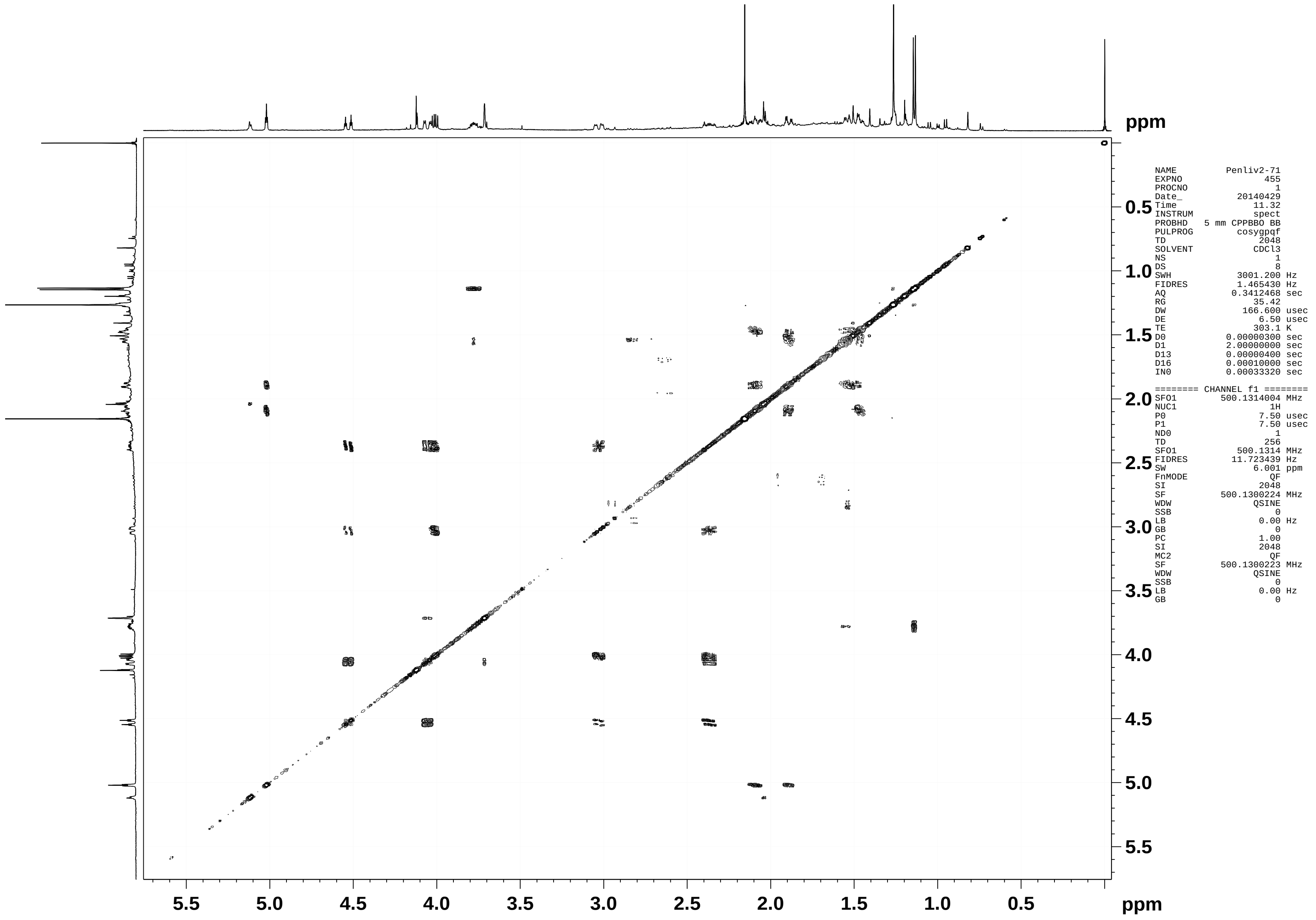

Figure S47. ^{13}C NMR (125 MHz, $CDCl_3$) spectrum of **6**.

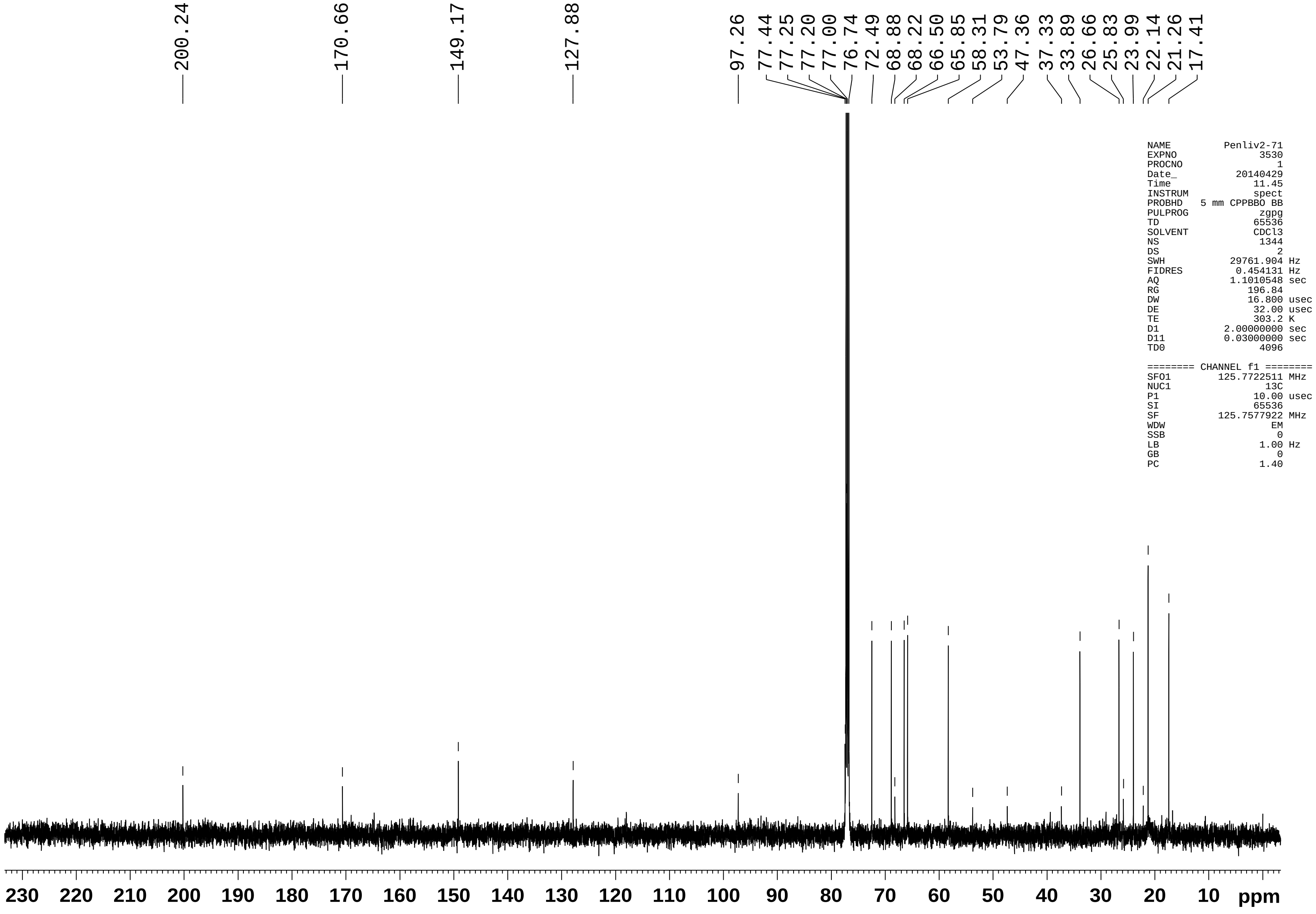

Figure S48. HSQC (500 MHz, $CDCl_3$) spectrum of **6**.

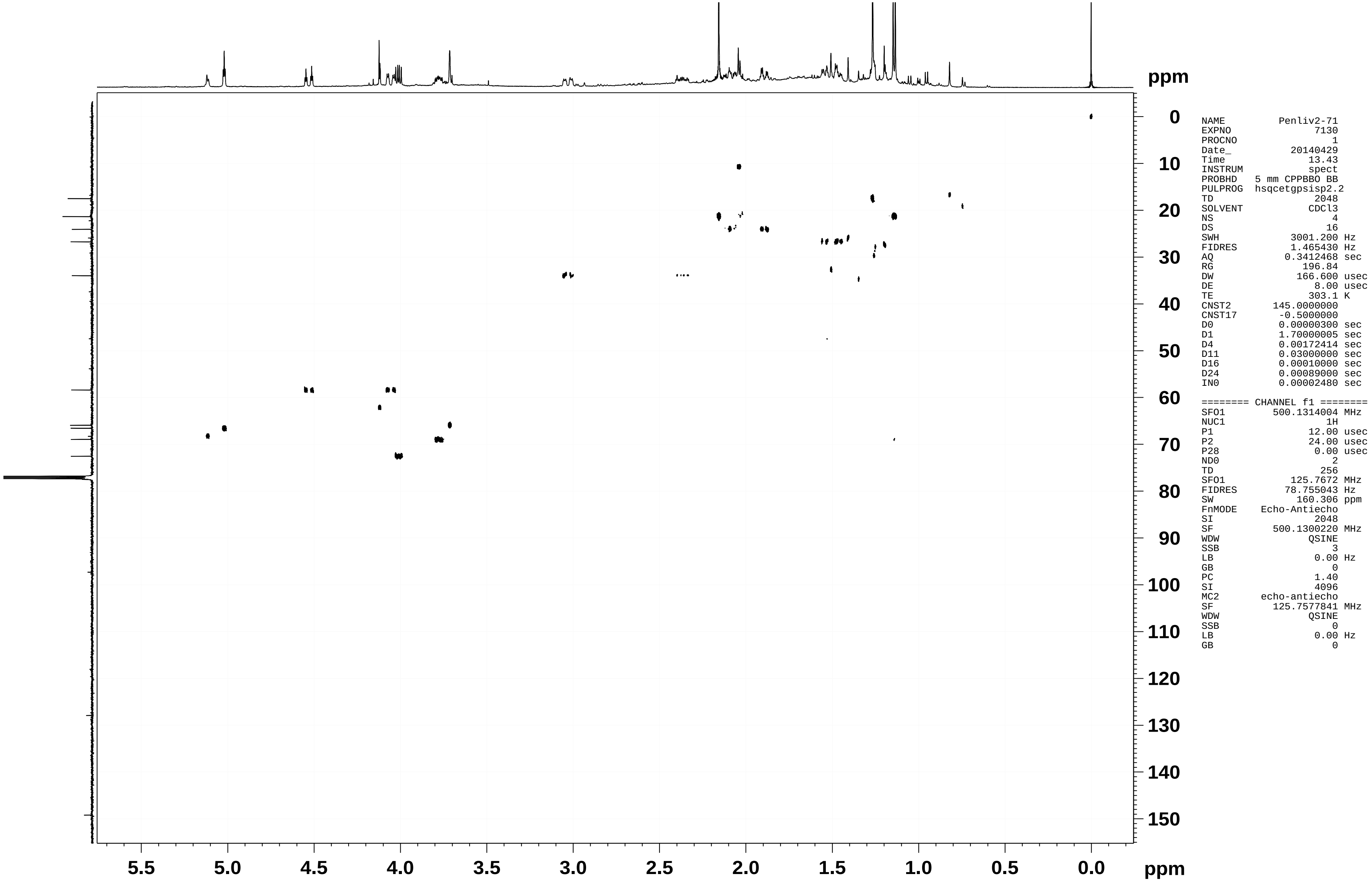

Figure S49. HMBC (500 MHz, $CDCl_3$) spectrum of **6**.

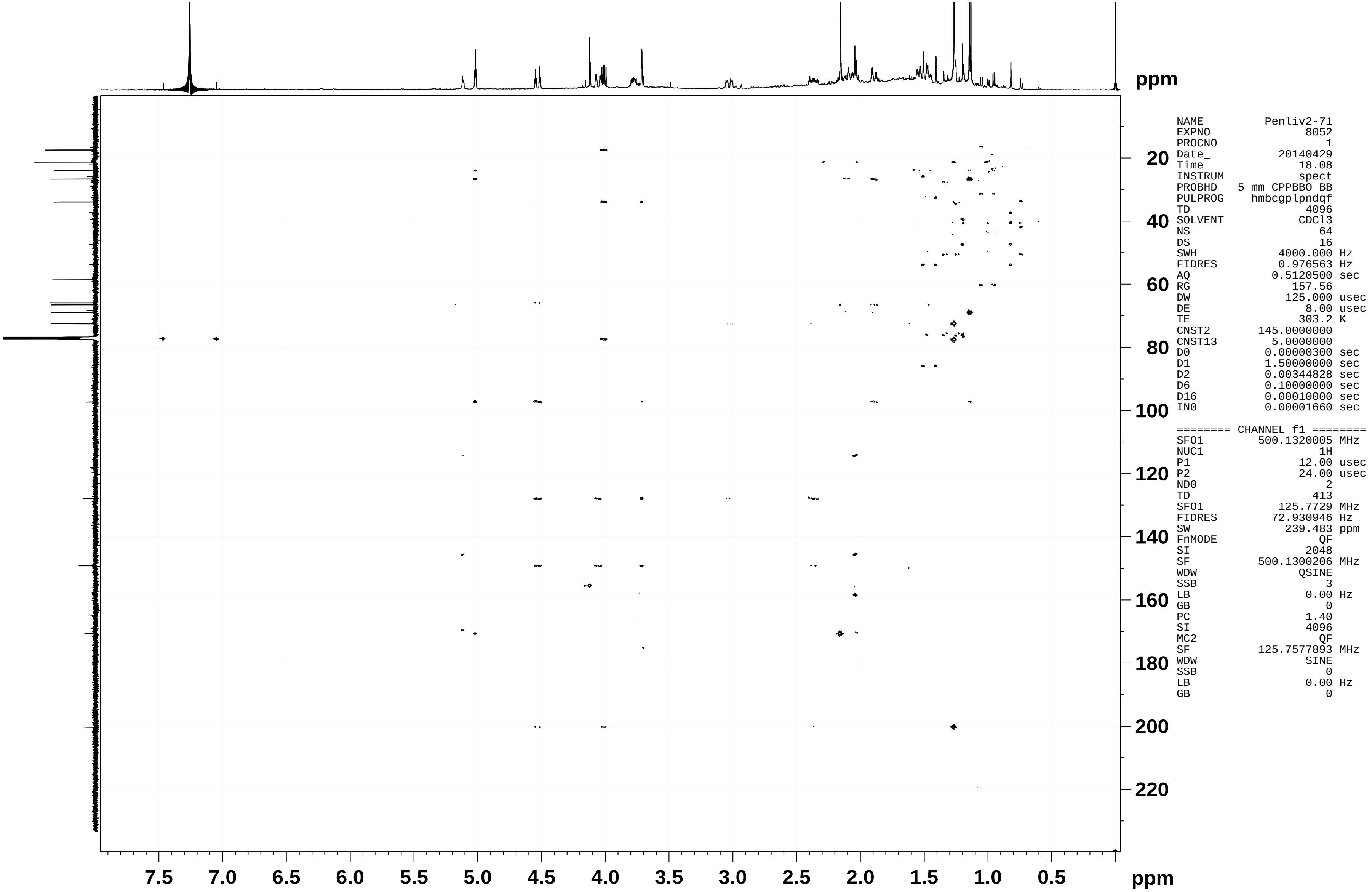

Figure S50. NOESY (500 MHz, $CDCl_3$) spectrum of **6**.

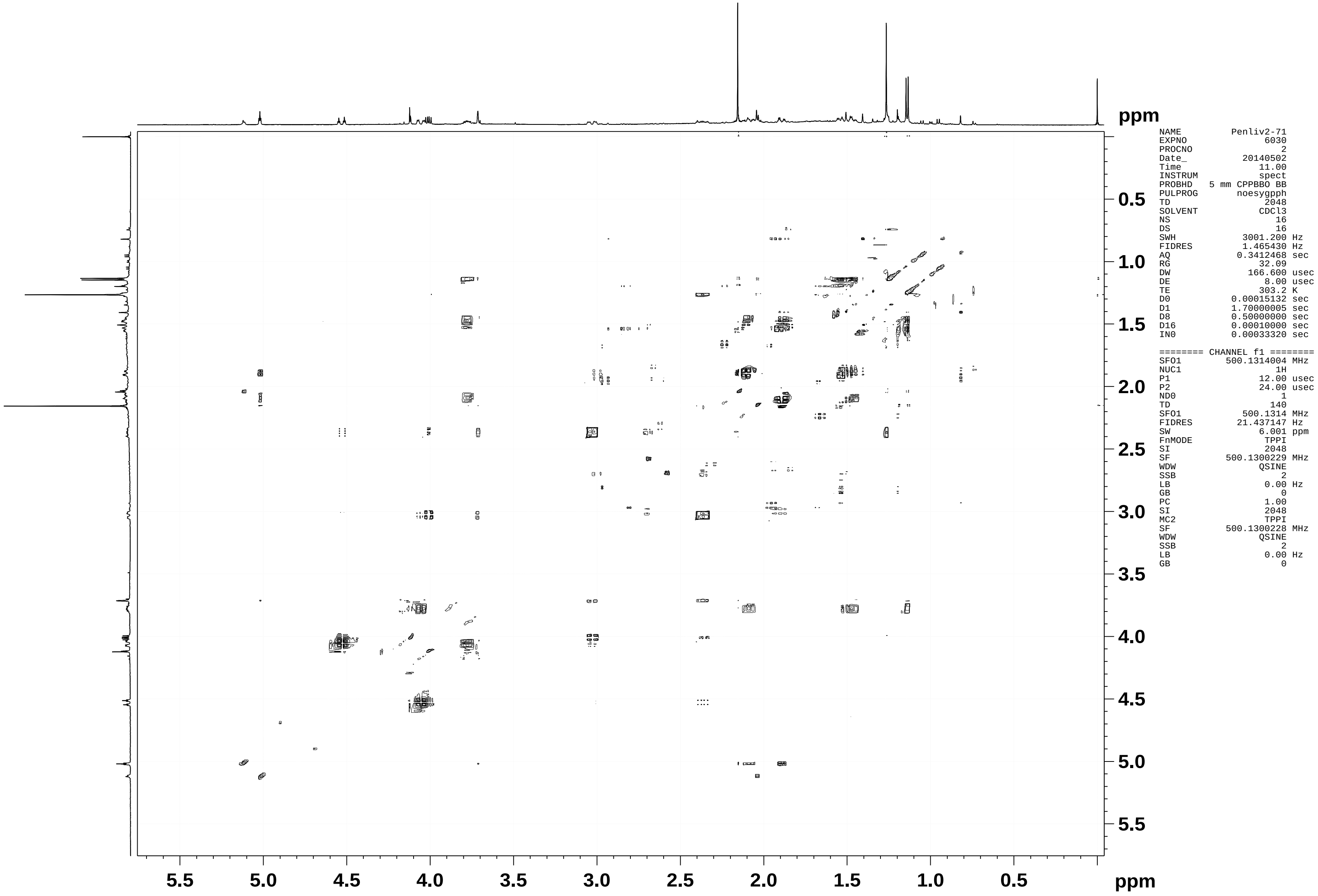

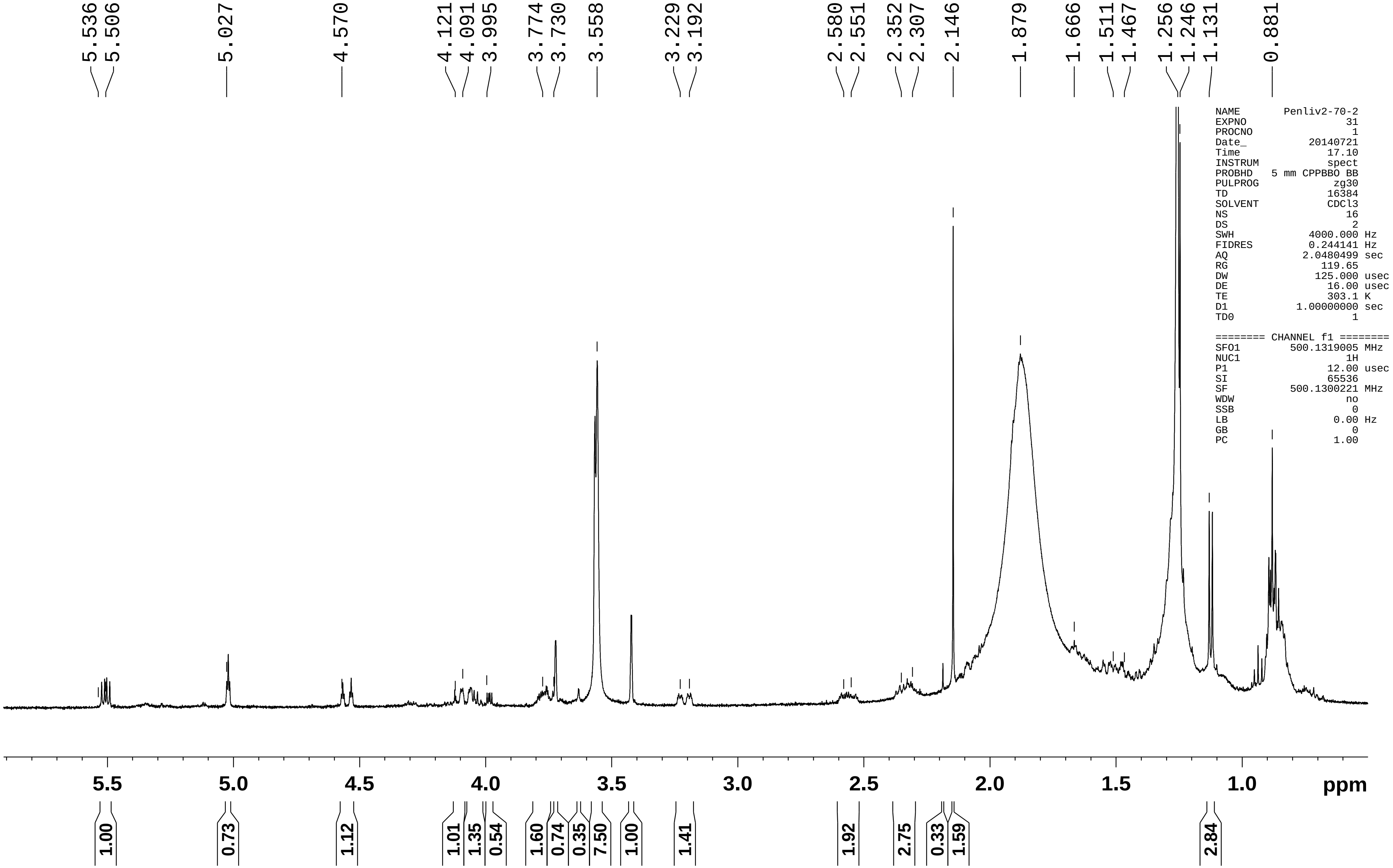

Figure S51. ^{1}H NMR (500 MHz, $CDCl_3$) spectrum of (*S*)-MTPA ester of **6a**.

Figure S52. ^{1}H-^{1}H COSY (500 MHz, $CDCl_3$) spectrum of (*S*)-MTPA ester of **6a**.

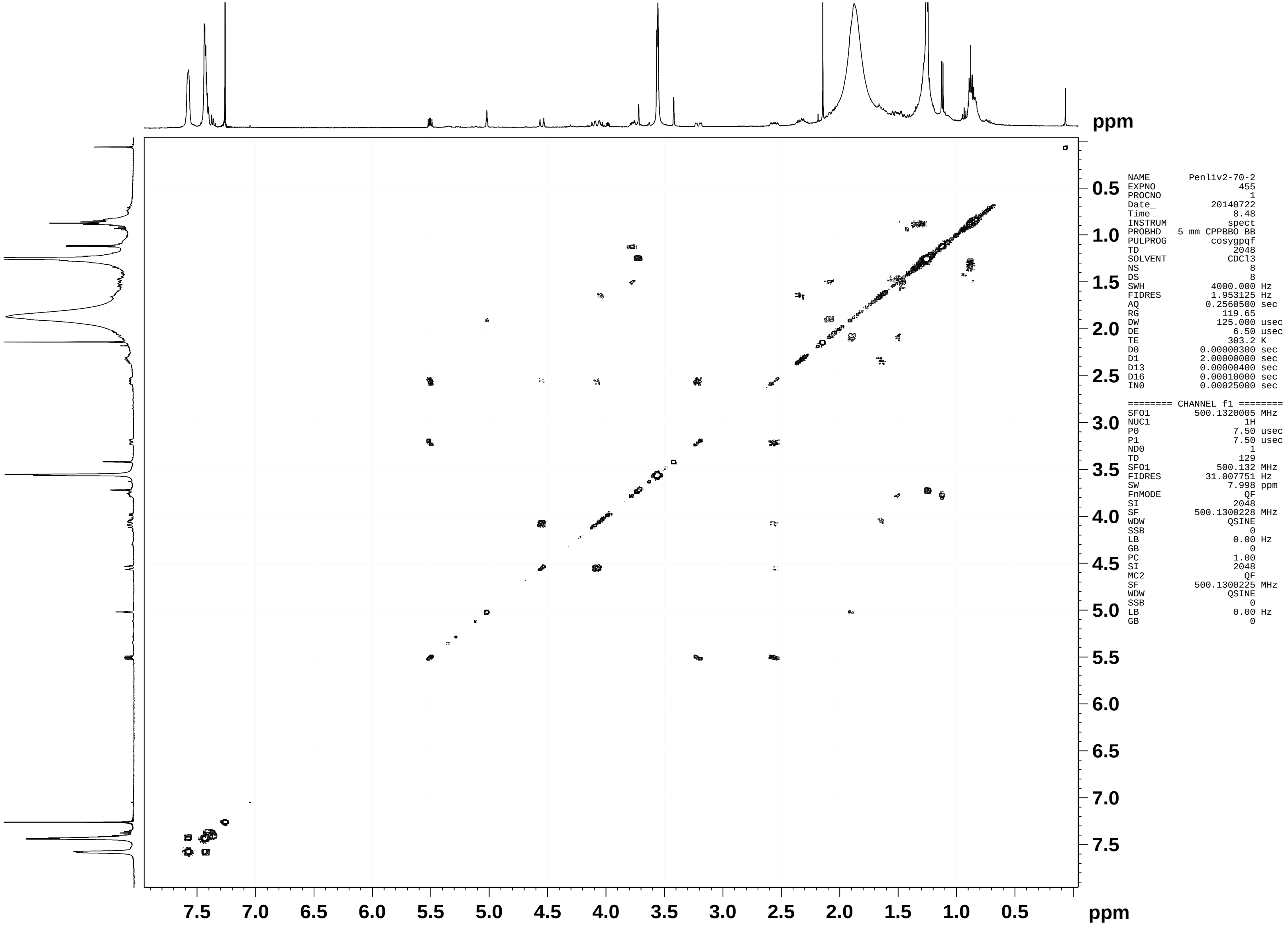

Figure S53. ^{1}H NMR (500 MHz, $CDCl_3$) spectrum of (*R*)-MTPA ester of **6b**.

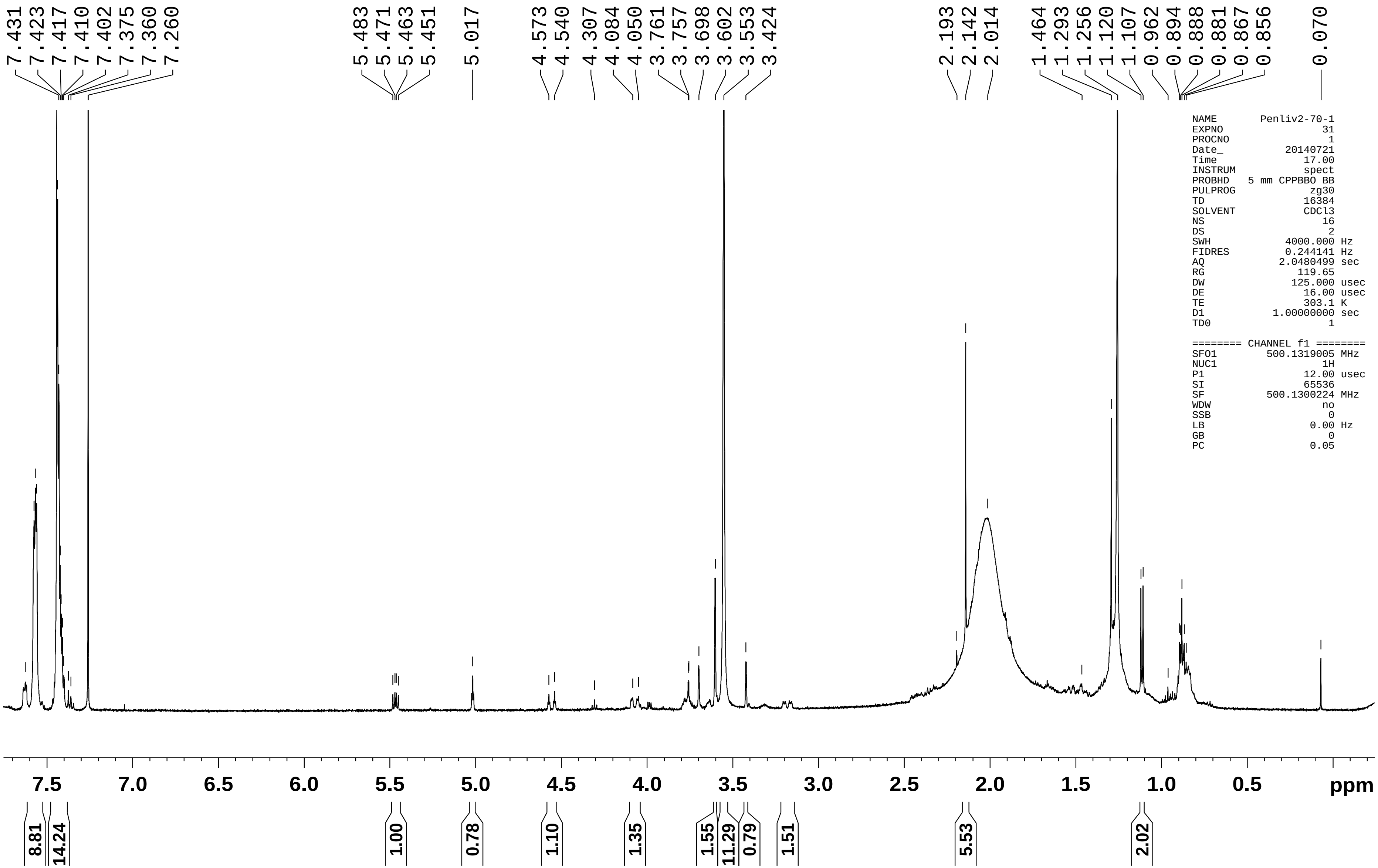

Figure S54. ^{1}H-^{1}H COSY (500 MHz, $CDCl_3$) spectrum of (*R*)-MTPA ester of **6b**.

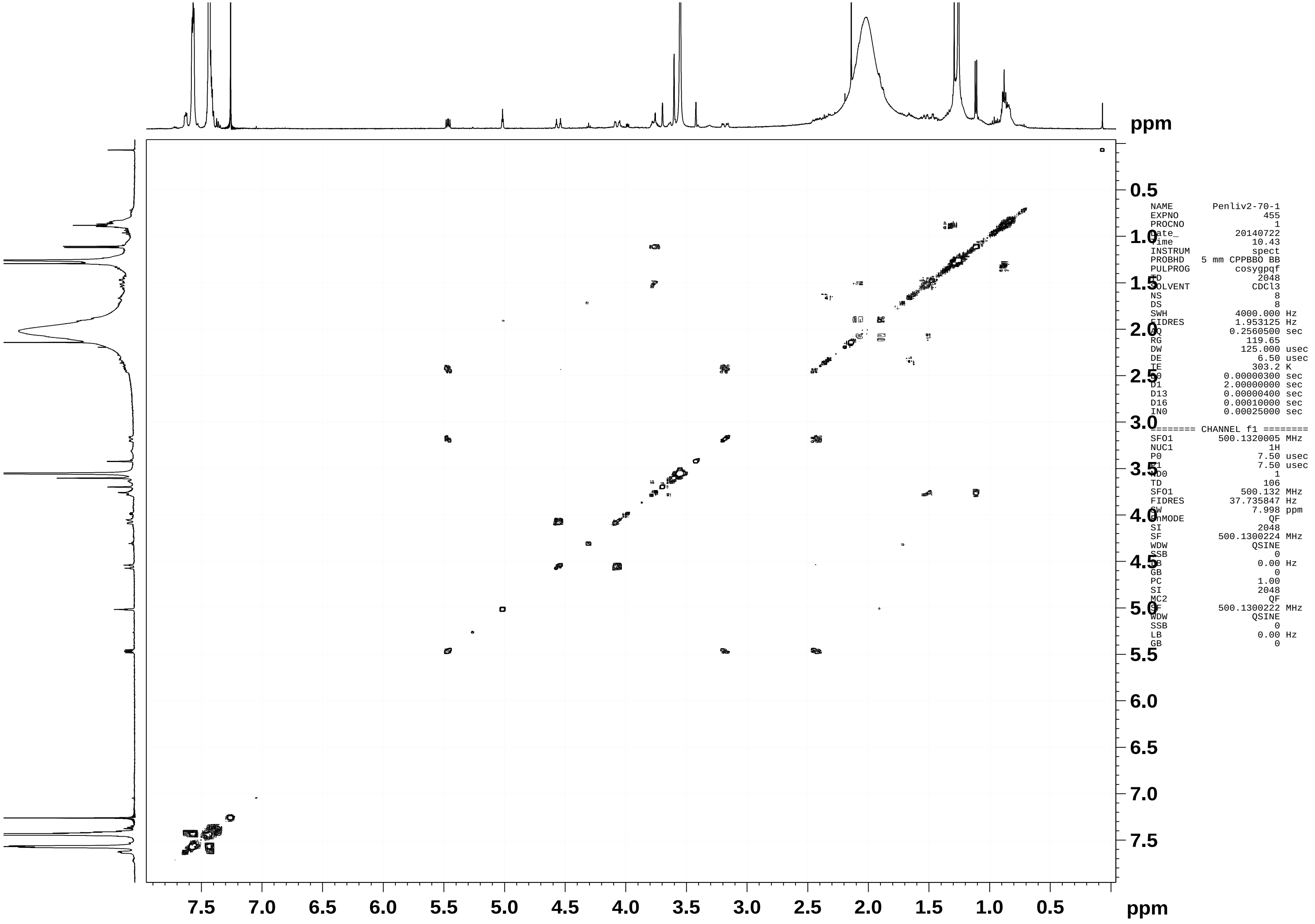

Figure S55. ^{1}H NMR (700 MHz, $CDCl_3$) spectrum of **7**.

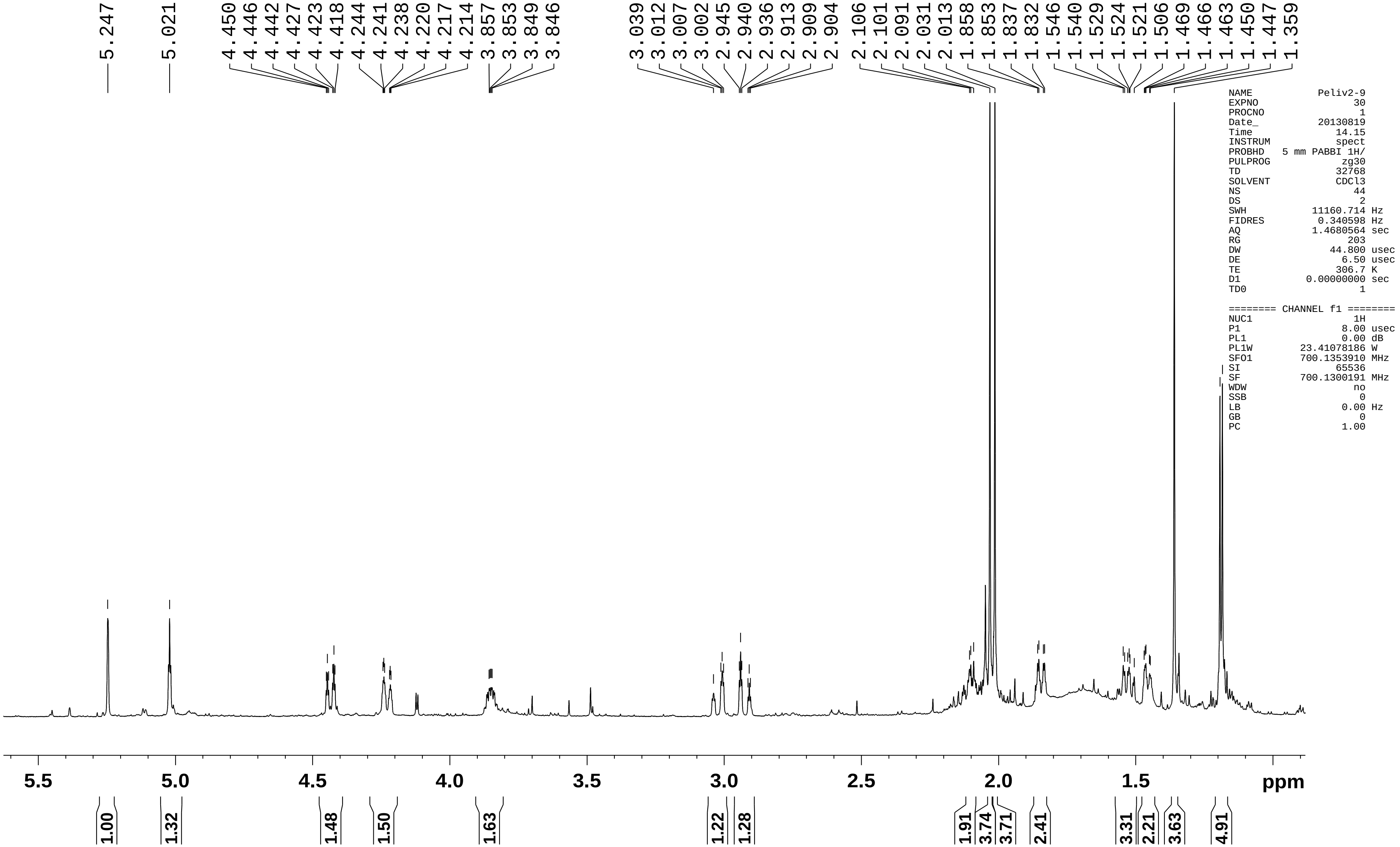

Figure S56. ^{1}H-^{1}H COSY (700 MHz, $CDCl_3$) spectrum of **7**.

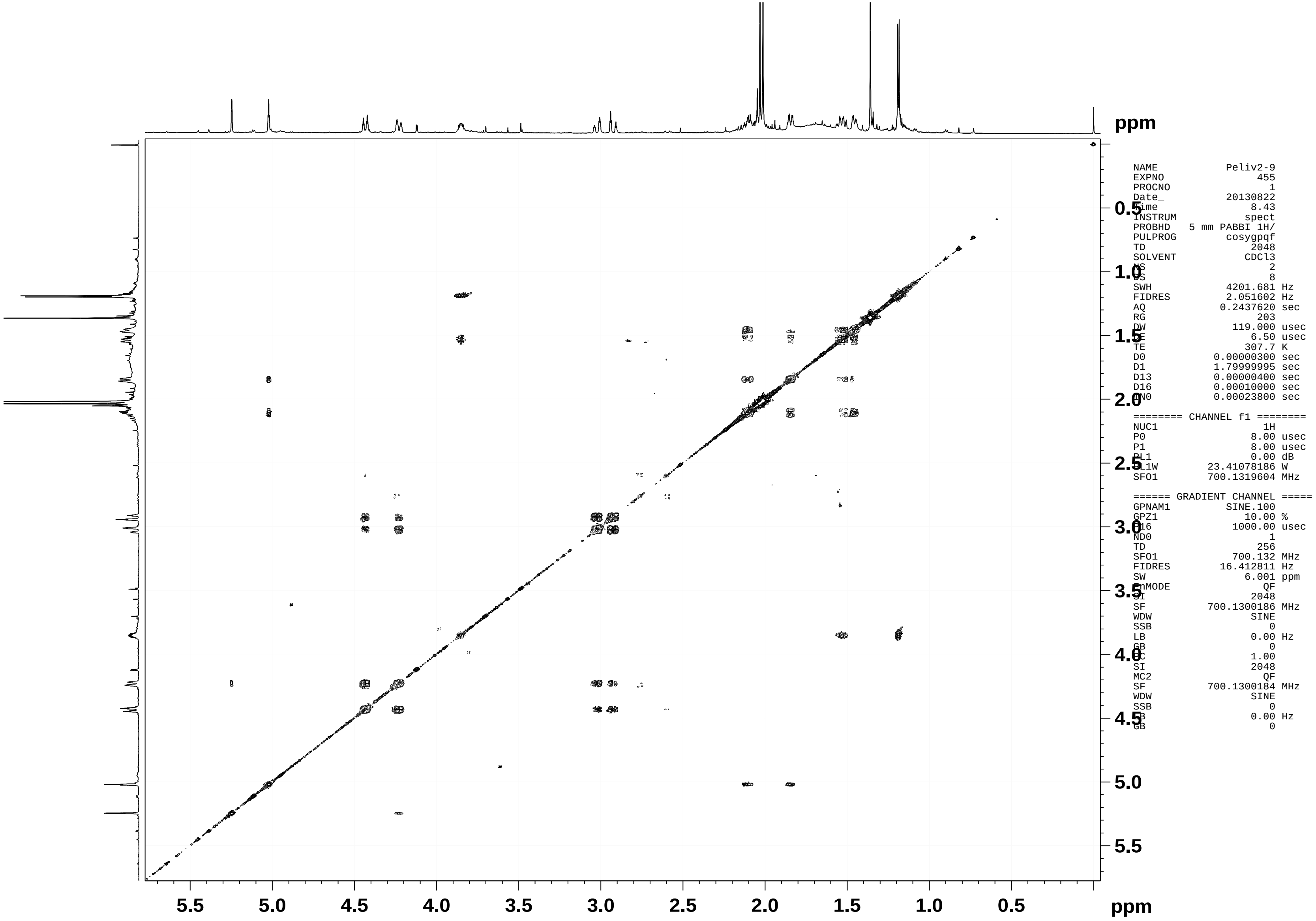

Figure S57. ^{13}C NMR (176 MHz, $CDCl_3$) spectrum of **7**.

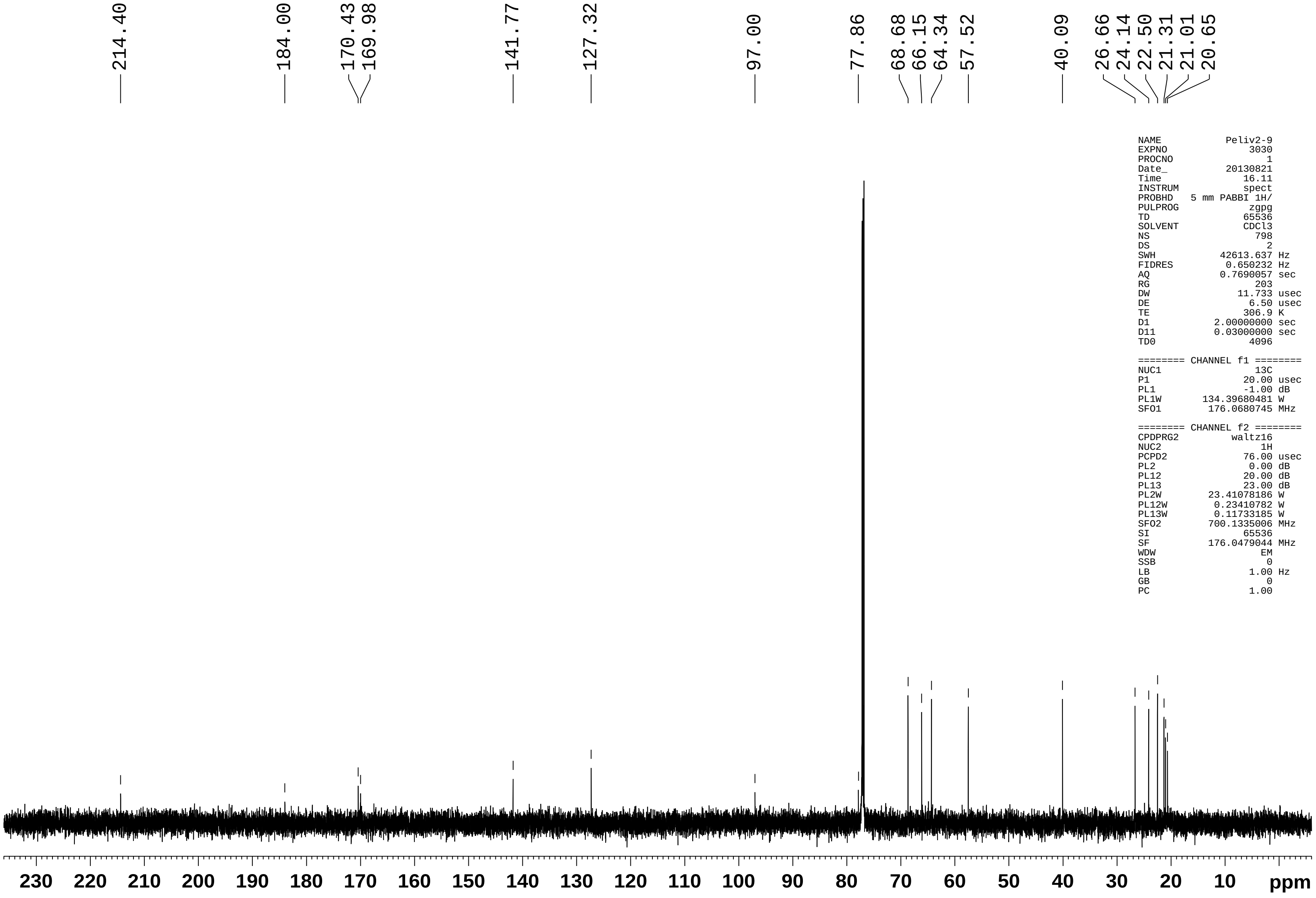

Figure S58. HSQC (700 MHz, $CDCl_3$) spectrum of **7**.

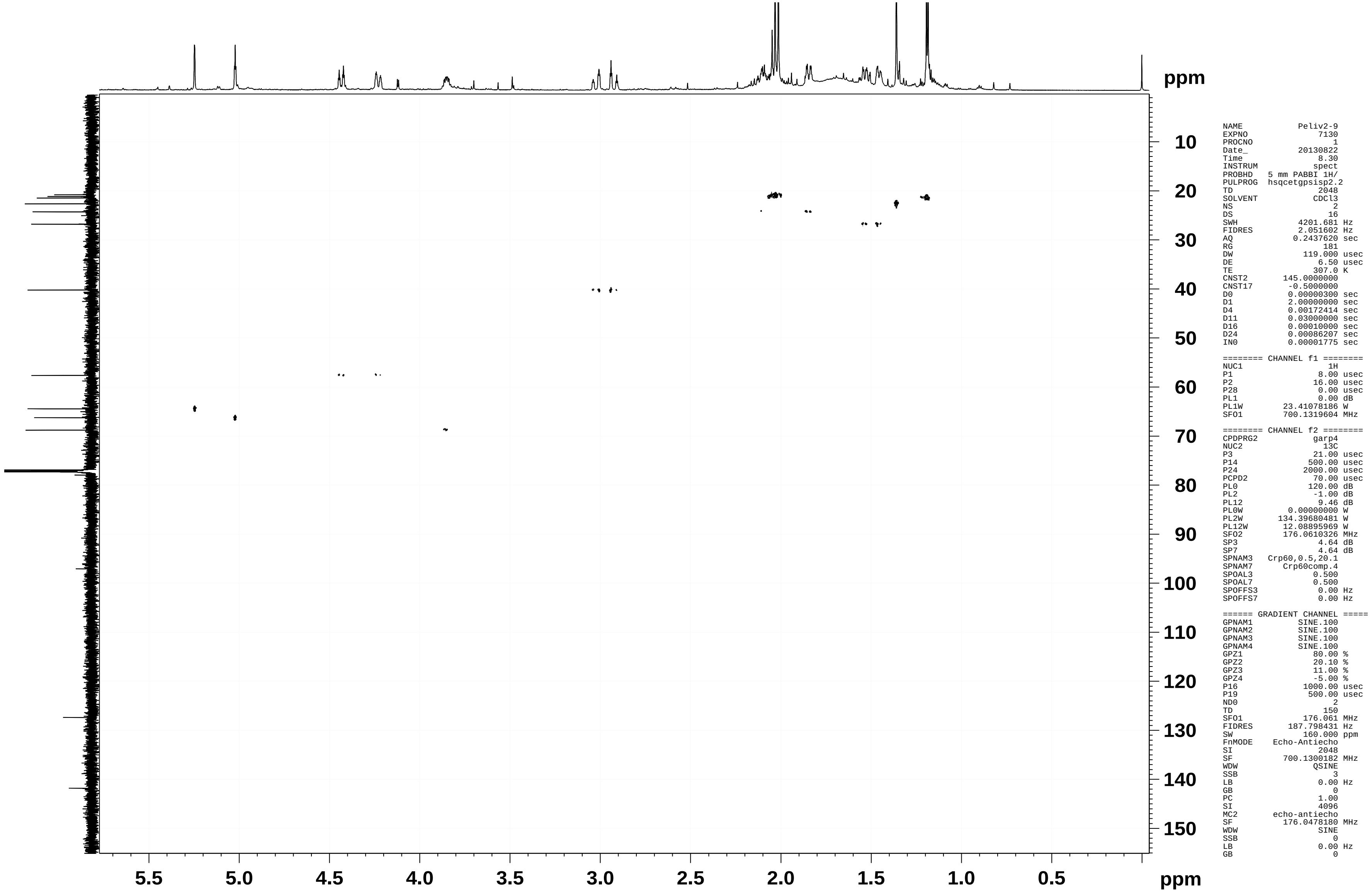

Figure S59. HMBC (700 MHz, $CDCl_3$) spectrum of **7**.

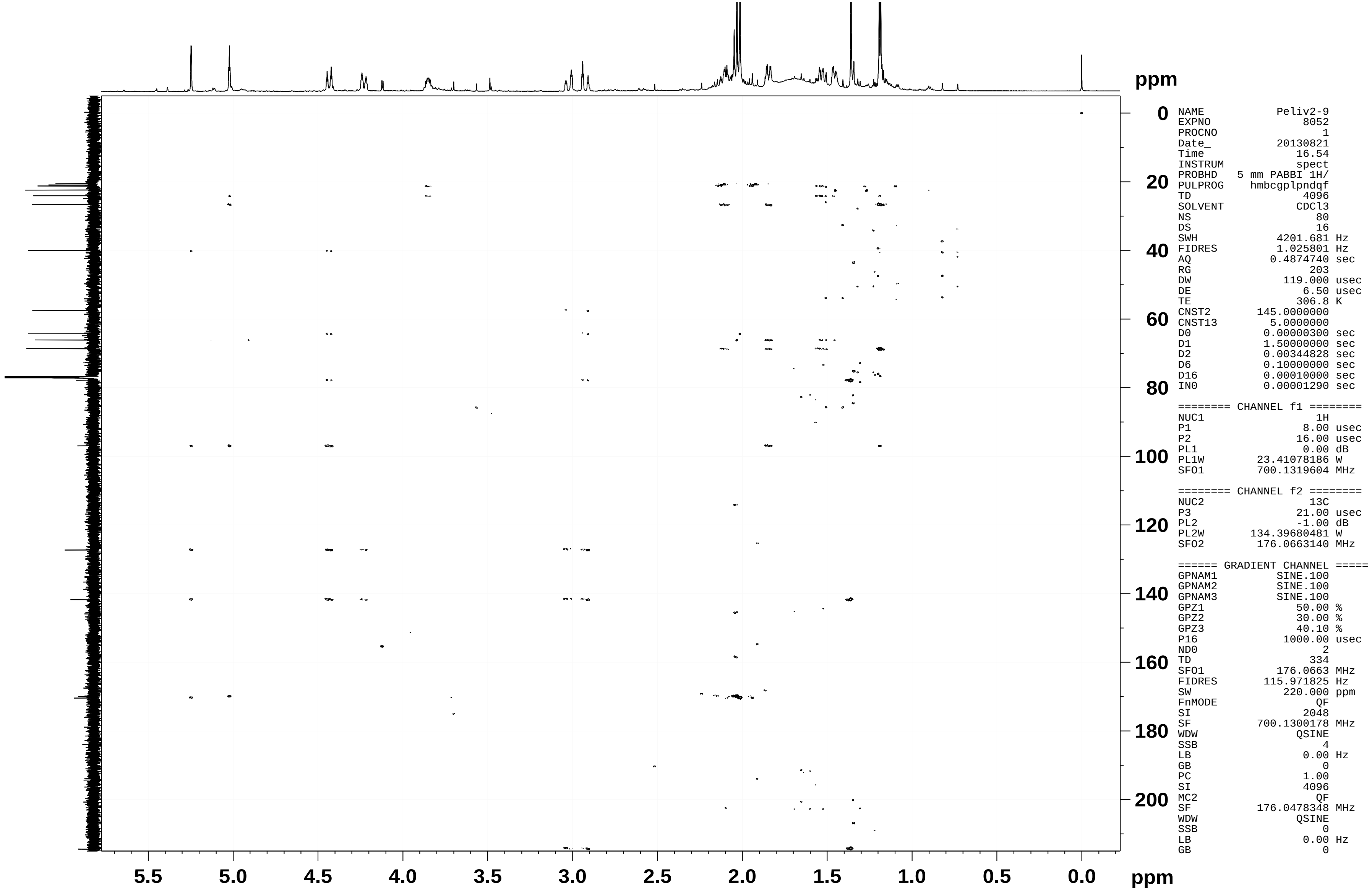

Figure S60. NOESY (700 MHz, $CDCl_3$) spectrum of **7**.

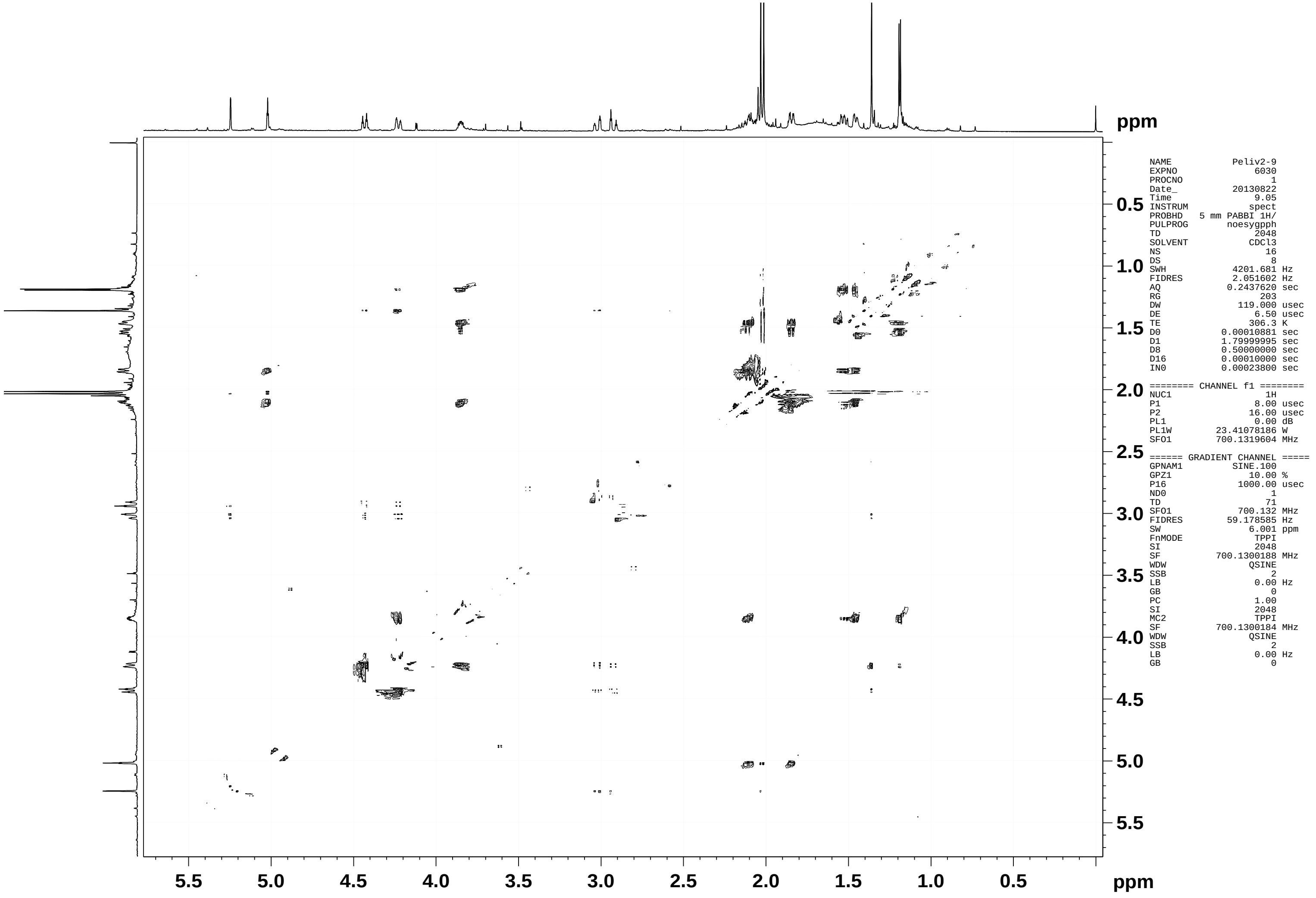

© 2014 by the authors; licensee MDPI, Basel, Switzerland. This article is an open access article distributed under the terms and conditions of the Creative Commons Attribution license (http://creativecommons.org/licenses/by/4.0/).
